# Design, Synthesis, and Biological Evaluation of Pyrazolyl Thioureas (PTUs) as SIRT1 and SIRT2 Inhibitors

**DOI:** 10.1002/cmdc.70489

**Published:** 2026-09-23

**Authors:** Matteo Lusardi, Cecilia Astigiano, Deianira Bellitto, Matteo Raineri, Giorgia Guccione, Annalisa Salis, Elena Cichero, Chiara Brullo, Marco Ponassi, Santina Bruzzone, Andrea Spallarossa

**Affiliations:** ^1^ Department of Pharmacy Università degli Studi di Genova Genova Italy; ^2^ Department of Experimental Medicine Università degli Studi di Genova Genova Italy; ^3^ AOM ‐ IRCCS Ospedale Policlinico San Martino Genova Italy

**Keywords:** antiproliferative activity, molecular docking, pyrazolyl thioureas, SIRT2 inhibitors, structure–activity relationship

## Abstract

A series of pyrazolyl‐thioureas was designed and synthesized as novel sirtuin 1 (SIRT1) and sirtuin 2 (SIRT2) inhibitors. Compounds were prepared through a multistep, divergent procedure starting from amino pyrazole intermediates. Biological evaluation revealed that several derivatives exhibited significant SIRT2 inhibitory activity, with compounds **6a** and **7f** emerging as the most promising inhibitors, showing IC_50_ values of 2.2 and 13.6 µM, respectively. Selected compounds displayed selective antiproliferative activity against HepG2 hepatocellular carcinoma cells, while showing limited cytotoxicity toward normal fibroblasts. The biological properties of **6a** and **7f** were further investigated by analyzing SIRT1 activity and correlating antiproliferative activity with tubulin acetylation. Docking simulations identified the molecular basis of SIRT1 and SIRT2 inhibition. Overall, these results identify pyrazolyl thioureas as a promising scaffold for novel SIRT1 and SIRT2 inhibitors with potential anticancer applications.

## Graphical abstract

Pyrazolyl thioureas were designed and synthesized as sirtuin 1 (SIRT1) and sirtuin 2 (SIRT2) inhibitors. **6a** (IC_50_ (SIRT1) = 5.3 µM; IC_50_ (SIRT2) = 2.2 µM) and **7f** (IC_50_ (SIRT1) = 12.4 µM; IC_50_ (SIRT2) = 13.6 µM) showed micromolar inhibition on target enzymes and HepG2‐based assays confirmed a SIRT2‐mediated effect on tubulin acetylation. Docking studies and metabolite prediction further characterize this novel class of sirtuin inhibitors.

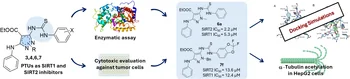

## 1 Introduction

Sirtuins (SIRTs) are Nicotinamide Adenine Dinucleotide (NAD^+^)‐dependent histone deacetylases involved in the regulation of fundamental functions [1], including glucose [2, 3, 4] and lipid metabolism [5, 6], mitochondrial biogenesis [7], DNA repair [8], oxidative stress [9], apoptosis [10, 11], and inflammation [12, 13]. The seven different SIRT isoforms (namely, SIRT1–7) so far identified in mammals share a common central catalytic domain but differ in N‐ and C‐terminal domains that account for their distinct localization and functions [14, 15]. In particular, SIRT1, SIRT6, and SIRT7 are mainly nuclear, whereas SIRT2 is predominantly cytoplasmatic and SIRT3–5 are primarily found in mitochondria [16]. As for their catalytic activity, SIRT1–3 mainly act as deacetylases [17], while SIRT4 and SIRT6 possess dual deacetylase and mono‐ADP‐ribosyltransferase activity [18, 19]. Although they were originally characterized as histone deacetylases, it is now well established that sirtuins possess broader deacylase activities and can remove several lysine acyl modifications (e.g., acetyl, succinyl, malonyl, glutaryl, long‐chain fatty acyl, and other acyl groups) in an isoform‐dependent manner [20]. More specifically, it is well established that SIRT5 exhibits robust desuccinylase, demalonylase, and deglutarylase activities, whereas SIRT6 preferentially removes long‐chain fatty acyl groups and can also regulate lysine deacetylation [17]. This functional diversity extends the regulatory role of sirtuins beyond the control of metabolism, mitochondrial function, stress responses, genome stability, and aging‐related processes. Additionally, the dependence of sirtuin activity on cellular NAD^+^ levels further positions these enzymes as key metabolic sensors linking the cellular energy status to epigenetic and post‐translational regulatory networks [17, 20].

SIRT dysregulation is implicated in various disorders (e.g., neurodegeneration, metabolic and cardiovascular diseases, and cancer) [1, 20], and SIRTs represent valuable pharmacological targets also for novel antiparasitic therapies [21, 22]. Among the seven mammalian sirtuin isoforms, SIRT1 and SIRT2 are the most extensively investigated. Besides their role in neurodegenerative disorders, both isoforms have been implicated in the regulation of key processes involved in tumor development and progression, including cell proliferation, apoptosis, metabolic adaptation, and response to therapy. In detail, SIRT1 is involved in different neurodegenerative conditions (e.g., Parkinson disease (PD), Alzheimer disease (AD), and Huntington disease (HD)) [23], and its inhibition reverses the neurodegenerative process and restores neural functions in the HD animal model [24]. Furthermore, SIRT1 is overexpressed in a wide range of tumors, including breast [25], liver [26, 27], and colorectal [28] cancers. However, SIRT1 plays a dual role, acting as or inactivating an onco‐suppressor depending on tumor types, stages, and microenvironment [29]. SIRT2 levels in the brain correlate with the onset and progression of several neurological disorders including AD, PD, and HD [23]. As for SIRT1, the role of SIRT2 in cancer is controversial as it functions either as a tumor suppressor or as an oncogene depending on the experimental conditions [30].

**FIGURE 1:**
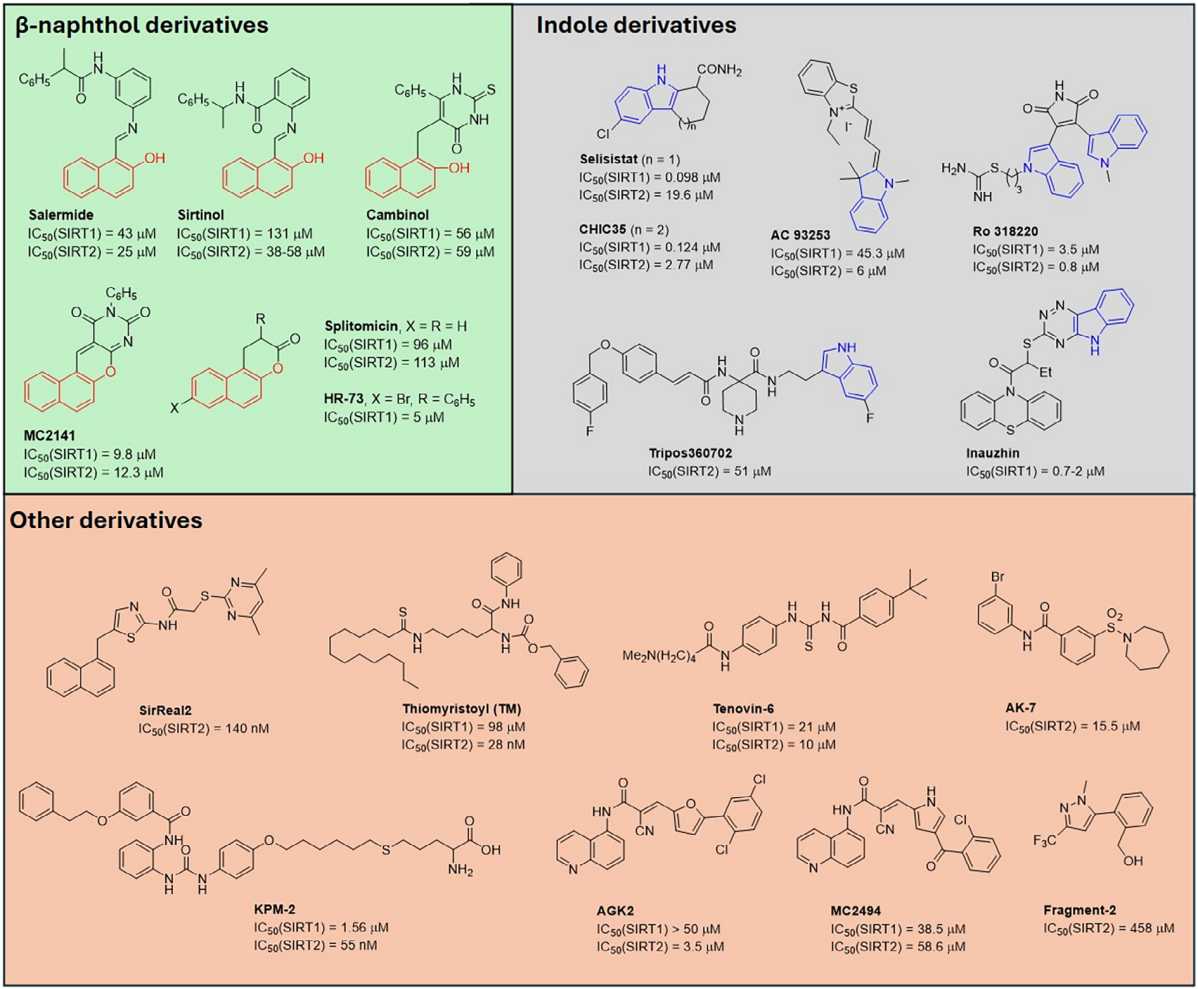
Different classes of SIRT1 and SIRT2 inhibitors and their IC_50_ values against SIRT1 and SIRT2.

The pharmacological potential of SIRTs prompted research into the development of activators and inhibitors of these enzymes, with a focus on SIRT1 and SIRT2 inhibitors [1]. As recently reviewed [1], different chemical classes of inhibitors have been so far identified (e.g., β‐naphthol, indole, thiourea, quinoline, and pyrazole derivatives; Figure 1). Notably, the compounds showed nanomolar or (sub)‐micromolar IC_50_ values with different selectivity against SIRT1 and SIRT2 enzymes depending on the chemical nucleus of the molecules. β‐Naphthol derivatives, such as **Sirtinol** and **Cambinol**, were among the first identified sirtuin inhibitors and have been widely used to investigate SIRT1 and SIRT2 functions. Although they exhibit limited potency and isoform selectivity, they provided early evidence supporting sirtuins as potential therapeutic targets in cancer [7]. At the same time, indole derivatives represent another important class of sirtuin inhibitors, with several compounds showing improved potency and selectivity toward SIRT1 and SIRT2 isoforms. These molecules have been extensively investigated for their anticancer potential and have contributed to the development of more selective sirtuin‐targeted therapeutic strategies [14]. Benzamide derivatives **KPM‐2** and **Thiomyristoyl** represent the most potent SIRT2 inhibitors so far (IC_50_ = 55 and 28 nM, respectively), still maintaining a sub‐micromolar activity against SIRT1. On the contrary, compound **SirReal2** displayed the highest selectivity against the SIRT2 enzyme with a calculated IC_50_ of 140 nM. Interestingly, most of the reported molecules contain an amide, urea, or thiourea functionalities, highlighting the importance of these carbonyl moieties inside the structure for the inhibitory activity.

**FIGURE 2:**
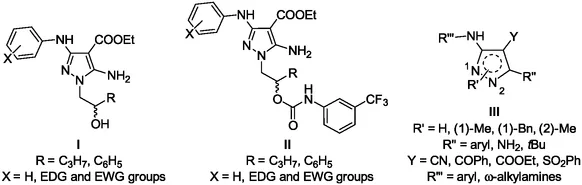
General structures of previously obtained pyrazole compounds.

Pyrazole represents a privileged scaffold in medicinal chemistry and is present in several biologically active compounds, including anticancer [31, 32, 33], anti‐inflammatory [34, 35], and antimalarial [36] agents. Additionally, amino‐pyrazoles have been identified as effective ligands for relevant pharmacological targets [37]. In previous studies, we developed one‐pot, two‐step procedures for the preparation of highly substituted amino‐pyrazoles [38, 39]. The so obtained compounds **I–III** (Figure 2) showed antiproliferative, antiangiogenetic, and antiplasmodial properties, further supporting the pharmacological potential of this chemical scaffold [40, 41, 42, 43]. Furthermore, the pyrazole substructure has been recently identified as a promising fragment for the development of novel SIRT2 inhibitors (**fragment‐2**; Figure 1) [44].

**FIGURE 3:**
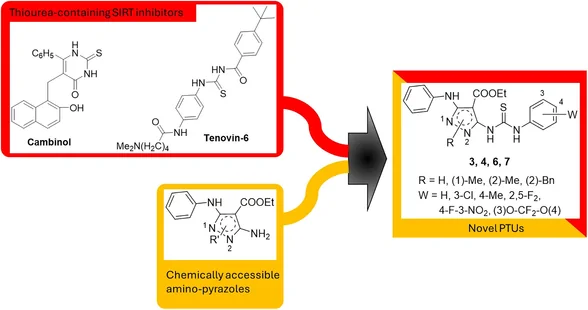
Design of novel PTUs as SIRT2 inhibitors.

**SCHEME 1:**
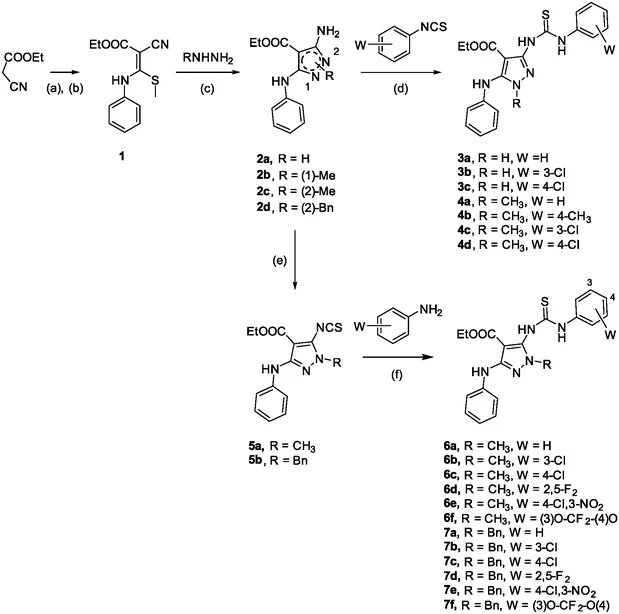
Synthesis of PTUs **3, 4, 6,** and **7**. *Reaction conditions*: (a) DMF_dry_, NaH, PhNCS, rt, 2 h; (b) MeI, rt, 16 h; (c) MeNHNH_2_ or BnNHNH_2_, 80 °C, 2–4 h; (d) ArNCS, toluene, reflux, 4–8 h; (e) anhydrous THF, NaH, CS_2_, I_2_, 0 °C to rt, 4 h; (f) DMF_dry_, ArNH_2_, rt, 1 h.

Our knowledge of amino‐pyrazole synthesis and the occurrence of the thiourea functionality in known sirtuins inhibitors (e.g., **cambinol** and **tenovins**; Figure 3) prompted us to evaluate pyrazolyl‐thioureas (PTUs) **3**, **4**, **6,** and **7** as an unreported chemotype for SIRT1 and SIRT2 inhibition (Figure 3; Scheme 1). The structure‐activity relationship (SAR) strategy was based on the introduction of substituents with different steric, electronic, and lipophilic properties on both the pyrazole nitrogens and the thiourea phenyl group keeping constant the anilino and carboxyethyl groups. Thus, derivative **3** bears a *N*‐unsubstituted pyrazole, whereas the thiourea phenyl group was kept unsubstituted (**3a**) or chlorine‐substituted at position meta (**3b**) or para (**3c**). To assess the effect on the activity of the pyrazole *N*‐substitution, the differently hindered methyl (positional isomers **4c,d** and **6b,c**) and benzyl (**7b,c**) analogs were prepared. To take also into account the influence of the substitution of the thiourea phenyl moiety, different substituants were considered besides the chlorine atoms. Thus, the thiourea phenyl ring was decorated with electron‐donating (**4b**) and electron‐withdrawing groups (**6d,e** and **7d,e**, respectively). Finally, to further extend SARs, the thiourea phenyl group was replaced by a bicyclic 2,2‐difluoro‐1,3‐benzodioxole substructure (**6f** and **7f**). The obtained compounds were tested for enzymatic inhibitory activity, antiproliferative effects, and the ability to modulate α‐tubulin acetylation. In addition, molecular docking studies were performed to rationalize the observed SAR and to provide insights into the molecular basis of SIRT1 and SIRT2 inhibition. Finally, the metabolites that can be formed from PTUs **3**, **4**, **6,** and **7** were calculated by GLORYx software.

## 2 Results and Discussion

### 2.1 Chemistry

The desired compounds were prepared according to a stepwise protocol, starting from the active methylene compound, ethyl cyanoacetate (Scheme 1). As previously reported [39], the one‐pot, two‐step condensation of ethyl cyanoacetate, phenyl isothiocyanate, and iodomethane in the presence of sodium hydride allowed the isolation of push‐pull *N*,*S*‐ketene acetals **1**, which was further condensed with (substituted)hydrazines (namely, hydrazine, methylhydrazine, and benzylhydrazine) to afford amino‐pyrazoles **2a–d**. Noteworthy, the cyclization reaction proved to be regioselective and occurred between the hydrazine nitrogen and the **1** nitrile group without involving its ester functionality. Additionally, the steric hindrance of the hydrazine substituent affected the reaction outcome. Thus, the condensation of **1** with methylhydrazine allowed the isolation of a mixture of the two *N*‐methyl pyrazole isomers (namely, **2b** and **2c**) [39]. Conversely, the reaction of **1** with the sterically hindered benzylhydrazine led to the unique formation of **2d**, with the benzyl group positioned far from the bulky anilino substructure. Key intermediates **2a–d** were converted to final PTUs using two distinct protocols based on the different reactivity of the amino group. In particular, amino‐pyrazoles **2a** and **2b** successfully reacted with properly substituted phenyl isothiocyanates to afford thioureas **3a–c** and **4a–d**. Differently, the presence of a *N*‐pyrazole substitution adjacent to the primary amino group prevented the reaction of **2c** and **2d** with aryl isothiocyanates. Therefore, intermediates **2c** and **2d** were converted into the corresponding isothiocyanates **5a,b** and then condensed with properly substituted anilines to afford PTUs **6a–f** and **7a–f**. This alternative chemical strategy included an additional synthetic step but extended the versatility of the synthetic procedure, taking advantage of the use of largely available aniline building blocks.

### 2.2 Antiproliferative Activity

The antiproliferative properties of PTUs **3**, **4**, **6,** and **7** were tested using the MTT assay at the fixed concentration of 10 µM (Table 1). A panel of seven tumor cell lines (MCF7, MDA‐MB231, SK‐Br3, SKMEL‐28, Hep‐G2, HeLa, and A549) and one normal human fibroblast cell line (GM‐6114) were considered. **Cis‐platinum** was used as the reference drug. Additionally, for those compounds showing growth percentage values lower than 50%, EC_50_ values were determined on sensitive cell lines.

**TABLE 1:** Antiproliferative activity of PTUs **3, 4, 6**, and **7**.^a^ EC_50_ values (µM) in brackets.

Cpd	Mean growth percentage, %							
	MCF7	MDA‐MB231	SkBR3	SKMEL28	HepG2	A549	HeLa	GM6114
**3a**	87	105	85	94	79	100	92	90
**3b**	88	99	99	105	94	84	100	90
**3c**	86	91	92	108	110	93	104	95
**4a**	98	138	104	112	68	102	101	88
**4b**	105	126	107	128	101	107	93	101
**4c**	80	115	96	111	119	87	112	112
**4d**	90	112	106	110	112	92	124	106
**6a**	105	103	109	96	64	102	112	99
**6b**	103	106	104	95	42 (16.7)	99	101	99
**6c**	90	108	100	93	32 (13.0)	81	74	91
**6d**	97	111	97	107	81	81	117	107
**6e**	69	103	93	94	45 (15.2)	51	79	91
**6f**	63	92	87	84	39 (13.1)	39 (11.7)	72	97
**7a**	86	91	94	74	57	78	86	87
**7b**	95	105	92	90	40 (13.1)	88	91	96
**7c**	73	76	82	60	46 (8.7)	61	54	63
**7d**	86	96	77	82	35 (15.3)	83	89	88
**7e**	85	100	84	77	32 (17.6)	72	65	94
**7f**	54	55	56	60	30 (10.4)	38 (9.1)	46 (10.1)	70
**Cis‐Pt**	73	86	71	44	38	59	29	40

a The mean of 3 determinations is shown (SD < 10% for all compounds).

Regardless of the phenyl thiourea substitution pattern, *N*‐unsubstituted pyrazoles **3** and *N*‐methyl‐substituted derivatives **4** did not show any significant antiproliferative activity, with growth inhibition values generally above 50% across all tested cell lines. A significant improvement was observed for compound **6**, in which the methyl group is located on the pyrazole nitrogen adjacent to the thiourea moiety. In particular, compounds **6b** and **6c** (positional isomers of **4c** and **4d**) showed selective antiproliferative activity against the HepG2 cell line, being as active as the reference drug and displaying EC_50_ values in the micromolar range. Similar antiproliferative activity was observed for compounds **6e** and **6f,** characterized by a 4‐chloro‐3‐nitrophenyl and a difluorobenzodioxol scaffold, respectively. Noteworthily, compound **6f** also showed activity against the A549 cell line (EC_50_ = 11.7 µM), suggesting a broader antiproliferative profile.

Benzyl‐substituted derivative **7** showed the most pronounced cellular activity. With the sole exception of unsubstituted compound **7a**, all derivatives in this series exhibited relevant antiproliferative activity against HepG2 cells with growth percentage values in the 30%–46% range and EC_50_ values of 8.7–17.6 µM. Compound **7f** was identified as the most active derivative and proved to be effective, at a micromolar concentration, also on the proliferation of A549 and HeLa cell lines. Remarkably, none of the tested compounds showed significant cytotoxic activity against normal fibroblast GM6114, thus indicating selective antiproliferative action against tumor cell lines.

### 2.3 Enzymatic Assays

To assess the SIRT2 inhibitory activity of PTUs **3**, **4**, **6,** and **7**, enzymatic assays were carried out on the purified recombinant enzyme (Table 2). **AGK2** was used as a reference compound. At 150 µM, all compounds showed a significant inhibitory effect, with fourteen out of nineteen molecules abrogating SIRT2 activity.

**TABLE 2:** SIRT2 inhibition percentages (I%) exerted by PTUs **3, 4, 6** and **7**.^a^

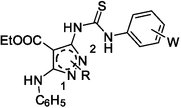				
	R	W	I, %	
			150 µM	20 µM
**3a**	H	H	100	39.1
**3b**	H	3‐Cl	100	77.2
**3c**	H	4‐Cl	100	49.0
**4a**	(1)‐Me	H	35	ND
**4b**	(1)‐Me	4‐Me	73	ND
**4c**	(1)‐Me	3‐Cl	100	45.5
**4d**	(1)‐Me	4‐Cl	100	49.7
**6a**	(2)‐Me	H	100	100
**6b**	(2)‐Me	3‐Cl	84	ND
**6c**	(2)‐Me	4‐Cl	87	ND
**6d**	(2)‐Me	2,5‐F2	100	63.4
**6e**	(2)‐Me	4‐Cl,3‐NO2	100	89.0
**6f**	(2)‐Me	OCF2O	100	49.0
**7a**	(2)‐Bn	H	86	ND
**7b**	(2)‐Bn	3‐Cl	100	65.9
**7c**	(2)‐Bn	4‐Cl	100	81.5
**7d**	(2)‐Bn	2,5‐F2	100	29
**7e**	(2)‐Bn	4‐Cl,3‐NO2	100	34.8
**7f**	(2)‐Bn	OCF2O	100	47.8
**AGK2**			97	ND

Abbreviation: ND, not determined.

a The average of 3 determinations is shown (SD < 20% for all compounds).

To derive SARs, the compounds showing I% values of 100% at 150 µM were evaluated at 20 µM. The activity of trisubstituted PTU **3** was affected by the nature of the substituent of the thiourea phenyl ring, with 3‐chloro‐substituted compound **3b** being more active than its unsubstituted (compound **3a**) or 4‐chloro‐substituted (compound **3c**) analogs, suggesting that meta‐substitution may promote more favorable interactions The insertion of a methyl group on the pyrazole nitrogen adjacent to the anilino group (compounds **4**) differently affected the activity according to the substituent on the thiourea moiety. Thus, the methyl substitution of the pyrazole nucleus was detrimental for unsubstituted or 3‐Cl‐substituted analogs (compare **3a** with **4a** and **3b** with **4c**), whereas it did not affect the activity of the 4‐chlorophenyl compound (compare **3c** with **4c**). Furthermore, the replacement of an electron‐withdrawing group (chlorine atom) with the electron‐donating methyl group strongly reduced SIRT2 inhibitory activity (compare **4d** with **4b**). The formal shift of the methyl group to the pyrazole nitrogen adjacent to the thiourea functionality strongly enhanced the activity of phenyl‐unsubstituted derivatives (compare **6a** with **4a**) but proved to diminish the inhibitory properties of chloro‐phenyl analogs (compare **6b** and **4c** and **6c** and **4d**). The insertion of phenyl groups disubstituted with differently hindered electron‐withdrawing groups (compounds **6d** and **6e**) or of a bicyclic difluorobenzodioxole moiety (**6f**) partially restored the inhibitory effect, still not obtaining inhibitors as effective as the unsubstituted precursor **6a**. Indeed, **6a** was identified as the most active compound of the series, abrogating SIRT2 activity at 20 μM, and exhibiting an IC_50_ value against SIRT2 of 2.2 µM. The replacement of the pyrazole methyl group with the hindered benzyl substructure strongly reduced activity of unsubstituted and disubstituted analogs (compare **6a** with **7a**, **6d** with **7d**, and **6e** with **7e**) and marginally affected the activity of the difluorobenzodioxole compound (**6f** and **7f**). Conversely, the insertion of a benzyl group proved to be beneficial within the chloro‐phenyl‐substituted series (compare **6b** and **6c** with **7b** and **7c**). Variations of the substituents on thiourea phenyl rings, or their replacement with the difluorobenzodioxole substructure, led to less active compounds (compare **7b**, **7c** and **7d**, **7e**, **7f**). Finally, activity toward SIRT1 was tested on the two most promising compounds (namely, **6a** and **7f**), as emerged from HepG2 cell viability and enzymatic assays (Tables 1 and 2). **6a** and **7f** showed comparable IC_50_ values for SIRT1 and SIRT2 (for **6a**: IC_50_ (SIRT1) = 5.3 µM, IC_50_ (SIRT2) = 2.2 µM; for **7f**: IC_50_ (SIRT1) = 12.4 µM; IC_50_ (SIRT2) = 13.6 µM), resulting in the methyl derivative being more effective than its benzyl congener.

### 2.4 Tubulin Acetylation

**FIGURE 4:**
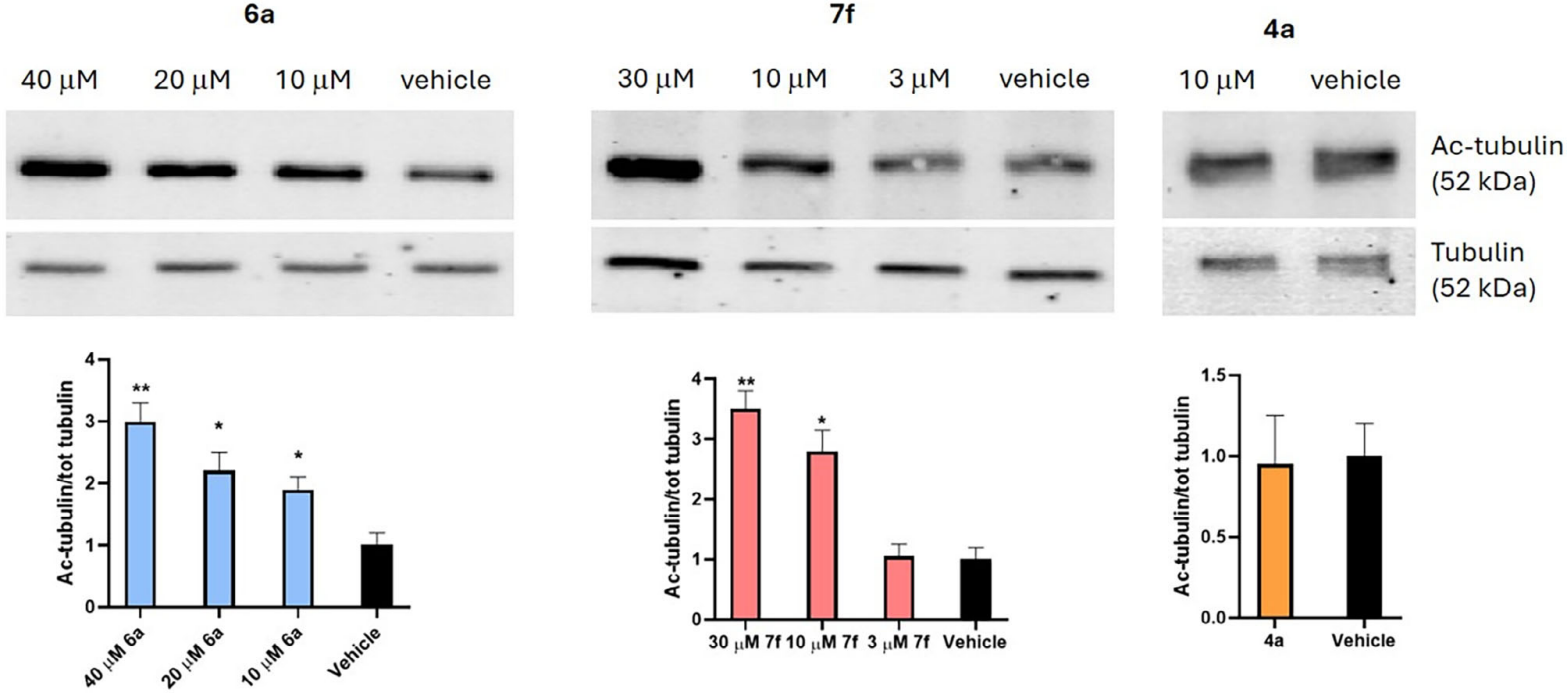
Effect of compounds **6a** and **7f** on α‐tubulin acetylation in HepG2 cells. Cells were incubated with the indicated concentrations of the compounds or the respective amount of vehicle DMSO and used for protein lysate generation. Total and acetylated α‐tubulin levels were detected by immunoblotting, quantified, normalized to total α‐tubulin, and expressed as percentage increase versus vehicle‐treated cells. One representative Western blot analysis and the mean ± SD of 3 quantifications are shown. The effect of compound **4a** is shown in parallel, as negative control. *p < 0.05 and **p < 0.01 versus vehicle.

A comparison between enzymatic inhibition and antiproliferative data highlights a partial correlation between SIRT2 inhibitory potency and cellular activity. Although compound **6a** emerged as the most potent inhibitor in the enzymatic assay, its antiproliferative effects were moderate. In contrast, compound **7f** exhibited more pronounced cellular activity, particularly against HepG2 cells, despite its lower potency at the enzymatic level. This apparent discrepancy suggests that additional factors, such as cell membrane permeability, intracellular accumulation, or compound stability, may play a key role in modulating the overall biological response in a cellular context. To further investigate this aspect and confirm effective intracellular target engagement, Western blot analyses were performed to quantify the acetylation levels of α‐tubulin, a well‐established substrate of SIRT2. Thus, HepG2 cells were treated with **6a** or **7f** at three different concentrations selected on the basis of the antiproliferative properties of the compounds (i.e., 10, 20, and 40 µM for **6a**; 3, 10, and 30 µM for **7f**). As acetylation levels of α‐tubulin are not only influenced by SIRT2 activity and are not directly linked to SIRT2 inhibition [45], PTU **4a** (10 µM), devoid of any significant SIRT2 inhibitory activity, was tested as a negative control. Derivatives **6a** and **7f** exhibited a dose‐dependent increase of acetylated α‐tubulin levels compared with control, untreated cells (Figure 4). The lack of any effect of **4a** on acetyl α‐tubulin levels provides indirect validation of SIRT2 target engagement by the active analogs **6a** and **7f**. Although **6a** inhibited SIRT2 more effectively than **7f** (IC_50_ (**6a**) = 2.2 µM; IC_50_ (**7f**) = 13.6 µM; Table 2), the benzyl derivative showed higher activity at 10 µM on tubulin acetylation, possibly due to its improved ability to cross HepG2 cell membranes. Moreover, this evidence supports the higher cytotoxic activity of the molecule in the MTT assay against liver cancer cells.

### 2.5 Docking Calculations

**FIGURE 5:**
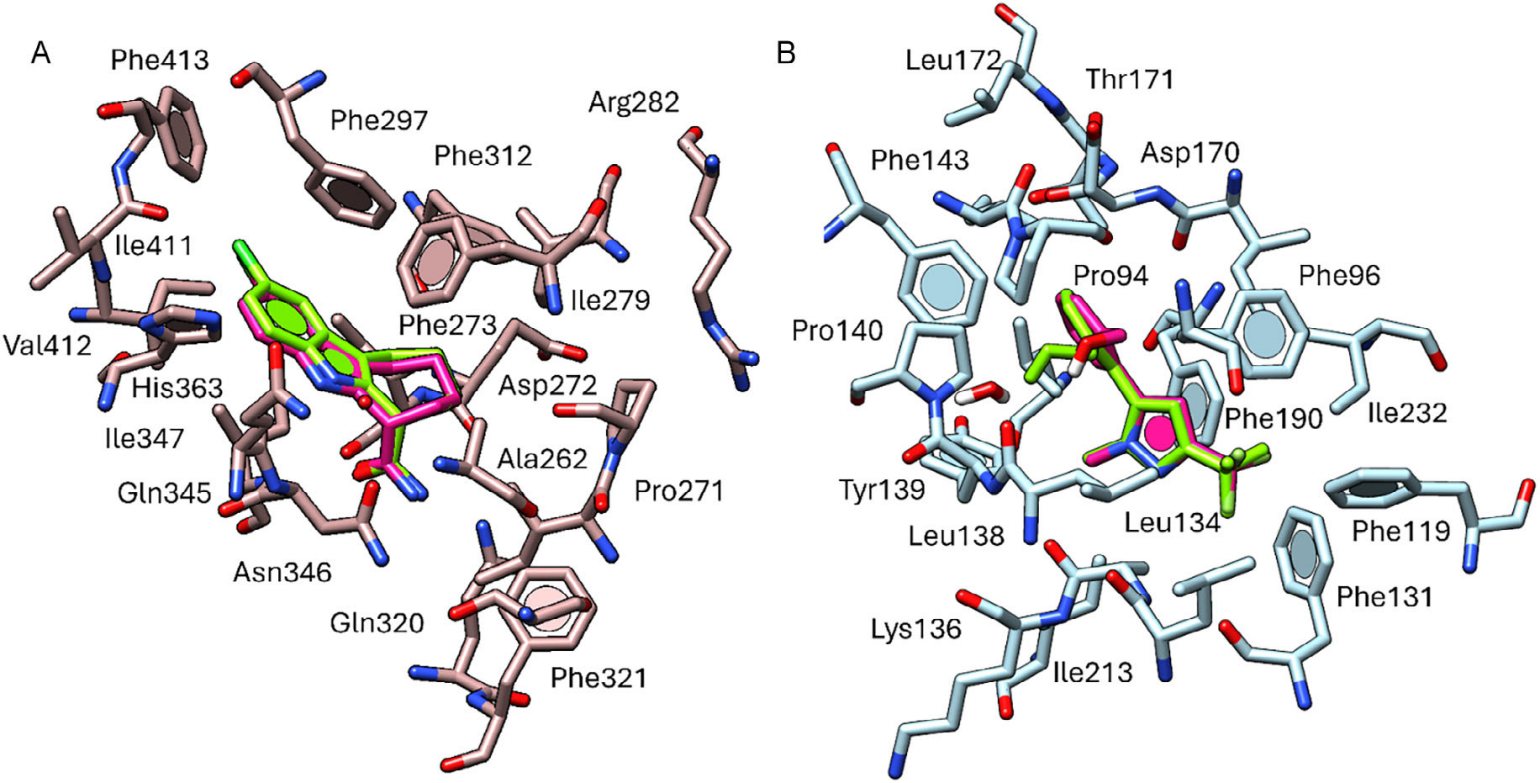
Comparison between docking and crystallographic SIRT1/**CHIC35** (panel A) and SIRT2/**fragment‐2** (panel B) complexes. The docking poses are colored purple whereas the crystallographic poses are shown in green.

To define the structural determinants for SIRT1/2 interactions, molecular modeling studies were carried out on compounds **6a** and **7f**. Docking simulations were conducted on the SIRT1 and SIRT2 catalytic domain, based on the corresponding X‐ray crystallographic data (SIRT1, PDB code = 4I5I; SIRT2, PDB code = 9FRU) [44, 46]. To assess the reliability of the docking protocol, a redocking procedure was applied on the SIRT1‐ and SIRT2‐co‐crystallized inhibitors: **CHIC35** (Figure 1) for SIRT1 and **fragment‐2** (Figure 1) for SIRT2 (Figure 5). The results pointed out comparable binding poses for the docking model and the crystallographic structure (RMSD values over all atoms heavy atoms = 0.4510 Å for **CHIC35**; RMSD value over all atoms = 0.4982 Å for **fragment‐2**) bearing the two top score poses estimated free binding energy (FBE) of −8.28029 and −6.63888 kcal mol^−1^, respectively. In particular, **CHIC35** occupies a narrow hydrophobic pocket delimited by Phe297, Phe309, Ile411, Val 412, Phe413 and Ile270, Pro271, Phe273, and Ile279 (Figure 5A).

The SIRT1/**CHIC35** complex is mainly stabilized by π−π stacking interaction between the ligand aromatic portion and Phe297 and Phe413 side chains and Van der Waals contacts between the cycloaliphatic moiety and Ile270 and Pro271. Additionally, H bonds and polar contacts occur between the carbazole nitrogen atom and Gln345 and between the amide group and Asn346.

**FIGURE 6:**
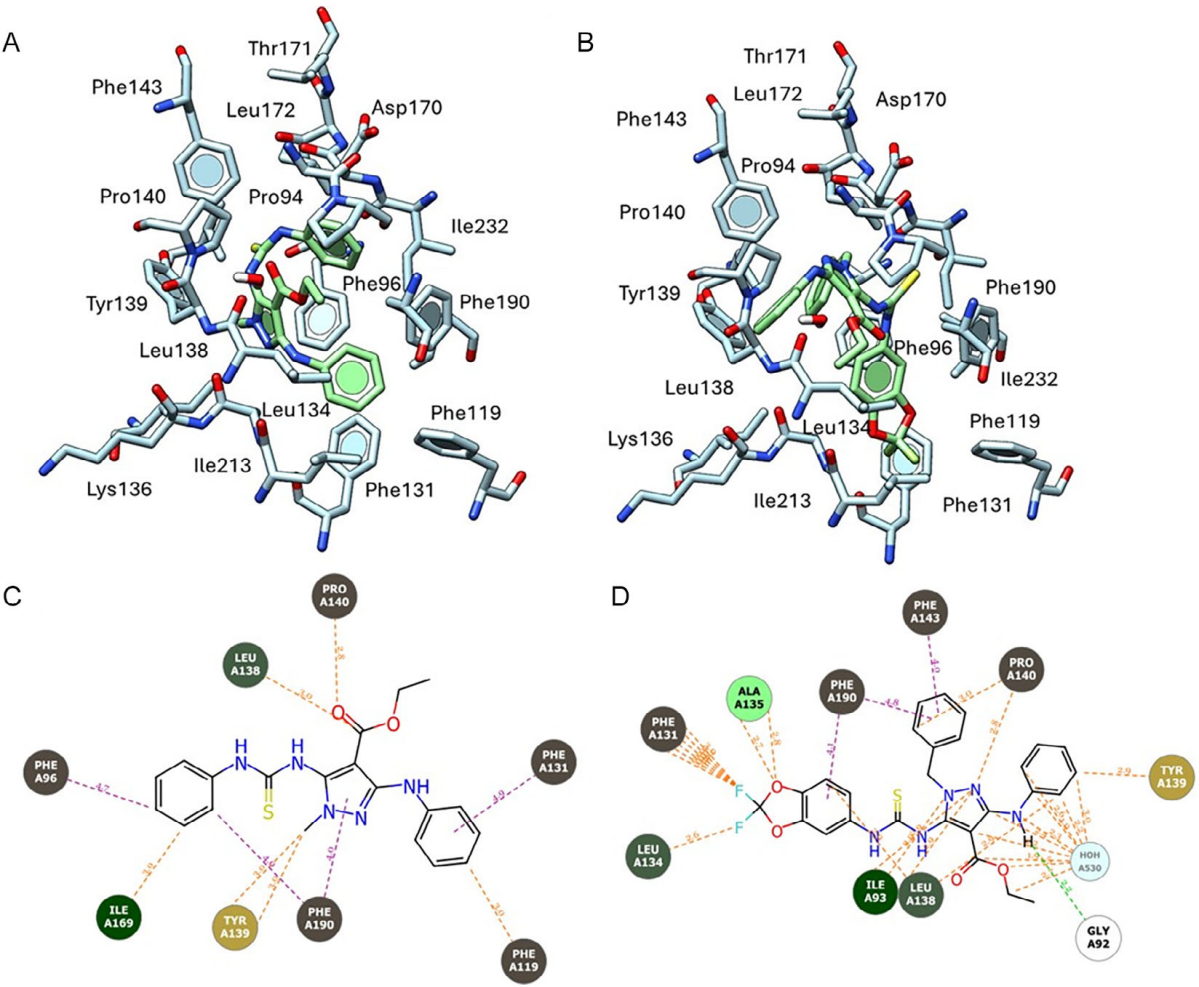
(A) **6a** docking pose (light green) within SIRT2 binding site. (B) **7f** docking pose (light green) within SIRT2 binding site. Residues lining the binding site are labeled. (C) Ligplot for the SIRT2/**6a** docking complex. (D) Ligplot for the SIRT2/**7f** docking complex.

In the SIRT2/**fragment‐2** complex, the ligand phenyl ring is engaged in π−π stacking interactions with Tyr139, Phe143, and Phe190, whereas the pyrazole core forms Van der Waals contacts with Leu138, Pro94, and Pro140 (Figure 5B). Moreover, the trifluoromethyl substituent displayed hydrophobic contacts with Phe96, Phe119, and Leu134, and the hydroxyl group is hydrogen‐bonded to Leu138 via a water bridge. Interestingly, this H‐bond would also occur between **6a** ester carbonyl and the Leu138 main chain in the SIRT2/**6a** docking complex (Figure 6A). The Van der Waals contacts between the thiourea phenyl ring and Pro94 and Ile232 and the π−π interactions between the aniline substructure and Phe96 and Phe131 side chains would provide further stabilization to the complex (Figure 6A). The estimated FBE values calculated for SIRT2/**6a** (FBE = −9.38719 kcal mol^−1^) and SIRT2/**fragment‐2** (FBE = −6.63888 kcal mol^−1^) complexes paralleled the measured differences between the IC_50_ values for the two inhibitors (2.2 vs. 458 μM).

In comparison with its analog **6a**, the bulkier **7f** would assume a different orientation within the SIRT2 binding site. In particular, the benzodioxole group would be oriented towards Phe96 and Phe131, while the aniline substituent was involved in π−π stacking with Tyr139 (Figure 6B). Interestingly, in both SIRT2/**6a** and SIRT2/**7f** (FBE = −10.18029 kcal mol^−1^) complexes, the ester group would be involved in polar contacts and water‐bridge‐mediated interactions with Leu138.

**FIGURE 7:**
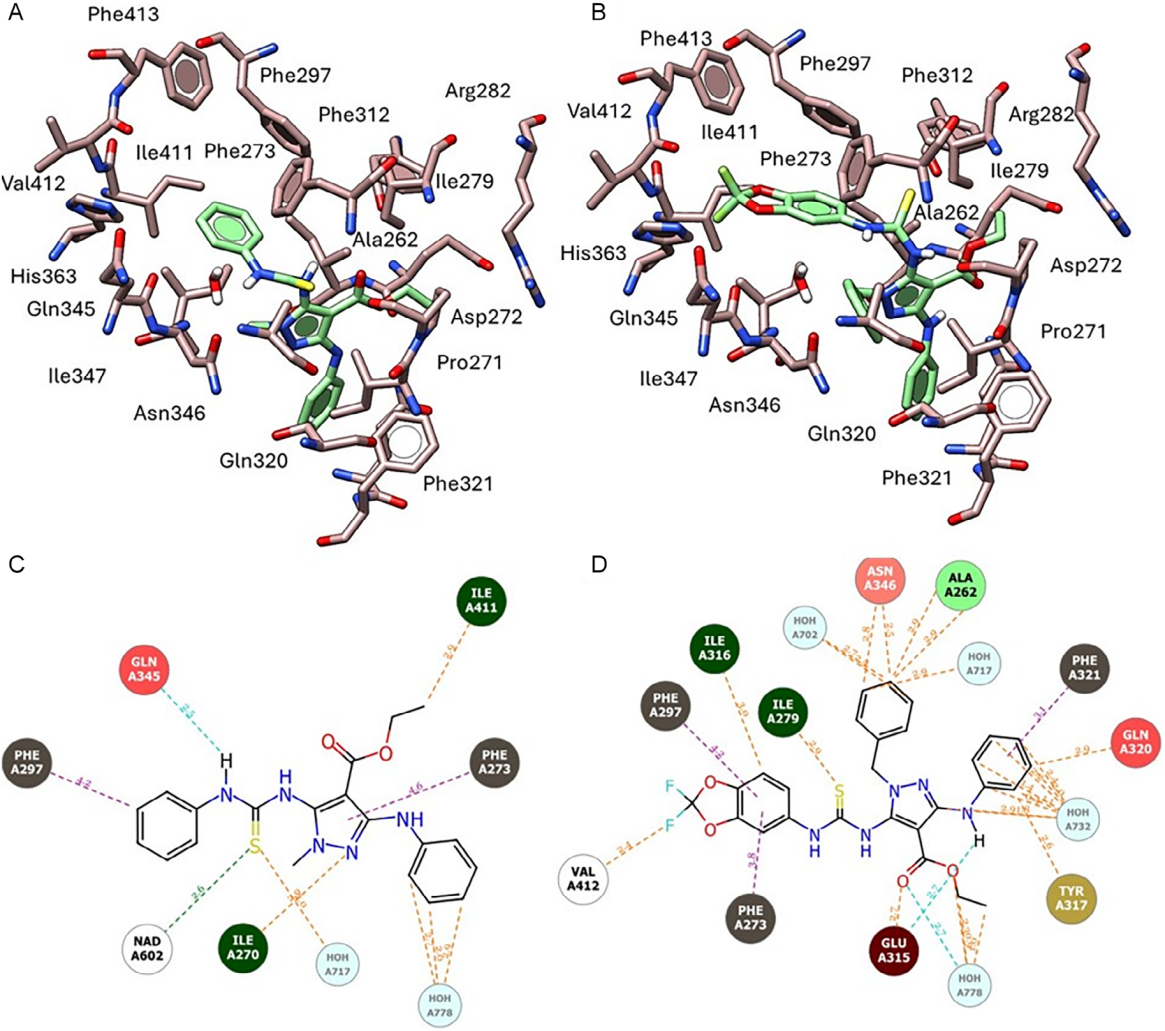
(A) **6a** (light purple) docking pose in SIRT1 binding cavity. (B) **7f** (light purple) docking pose in SIRT1 binding cavity. The residues lining the binding site are labeled. (C) Ligplot for the SIRT1/**6a** docking complex. (D) Ligplot for the SIRT1/**7f** docking complex.

To achieve SIRT1 inhibition, docking simulations suggested that *N*‐methyl pyrazole **6a** would be more effective than its benzyl analog **7f**. In fact, only in the SIRT1/**6a** docking complex, the thiourea NH group of the ligand would assume a similar orientation to that of **CHIC35** carbazole nitrogen, forming water‐bridge‐mediated contacts with Gln345 and Asn346 (estimated FBE = −9.07590 kcal mol^−1^) (Figure 7A).

Conversely, the docking orientation calculated for **7f** would prevent any polar contact between the thiourea group and SIRT1, with a dramatic decrease of the binding affinity (estimated FBE = −5.25155 kcal mol^−1^) (Figure 7B). In both SIRT1/**6a** and SIRT1/**7f** docking complexes, the thiourea aromatic rings of the ligands would be able to interact with Phe297, Phe309, Ile411, Val 412, and Phe413; noteworthy, these residues also established contacts also with the co‐crystallized **CHIC35** ligand [46]. In addition, the pyrazole ring, the aniline, and ester substituents displayed π−π stacking and hydrophobic contacts with Phe321 and with Pro271 and Ile279, respectively.

### 2.6 Phase 1 Metabolites Prediction

To identify potential metabolic liabilities associated with PTUs **3, 4, 6, 7**, a prediction of phase 1 metabolites was carried out by the GLORYx platform [47]. The hypothesized metabolites were ranked according to a priority score, ranging from 0 to 1. The scores are directly proportional to the probability of the metabolite (priority score > 0.7: high probability; priority score 0.3–0.7: intermediate probability; priority score < 0.3: low probability). As detailed in the supplementary information file (Figures S46–S64), the predicted metabolic reaction on the prepared PTUs include *S*‐oxidation of the thiourea functionality (probability score = 0.30–0.34) and aromatic oxidation on either the thiourea *N*‐phenyl ring or anilino moiety (probability score = 0.22–0.32). For compounds **7**, the oxidation of the benzylic aromatic ring has been calculated as a low‐probability metabolic reaction. Noteworthily, the modification associated with the highest probability score was the oxidation of the tolyl methyl group in compound **4b** (probability score = 0.47), thus predicting for the prepared PTUs good metabolic stability.

## 3 Conclusion

A novel series of pyrazolyl thioureas were designed and synthesized as SIRT1 and SIRT2 inhibitors by combining a privileged pyrazole scaffold with a thiourea functionality. The developed synthetic strategy enabled the efficient preparation of structurally diverse derivatives, allowing a systematic investigation of SARs. Enzyme‐ and cell‐based biological evaluations were carried out in parallel. Several compounds showed significant SIRT2 inhibitory activity, with **6a** (IC_50_ SIRT2 = 2.2 µM) and **7f** (IC_50_ SIRT2 = 13.6 µM) identified as the most interesting compounds in terms of enzyme inhibition and antiproliferative activity. SAR analysis revealed that both the substitution pattern on the pyrazole core and the nature of the thiourea phenyl group critically influence activity, with optimal SIRT2 inhibition requiring a balanced steric and electronic profile. Docking studies provided a molecular rationale for these observations, highlighting key interactions within the SIRT2 binding pocket and suggesting possible determinants for SIRT1 and SIRT2 binding.

Interestingly, **6a** and **7f** induced a dose‐dependent increase of α‐tubulin acetylation in HepG2‐based assays, confirming effective intracellular target engagement. The lack of cytotoxicity toward normal fibroblasts further supports a favorable selectivity profile.

Overall, the results demonstrate the strength of the design strategy, leading to the discovery of pyrazolyl thioureas as a highly promising and tunable scaffold for SIRT1 and SIRT2 inhibition. The combined enzymatic, cellular, docking, and metabolite prediction data provide a solid foundation for future optimization and further development of these compounds as a novel class of pharmacologically relevant agents.

## 4 Experimental Section

### 4.1 Chemistry

Benzylhydrazine oxalate, aryl isothiocyanates, phenyl aniline, and reagents (60% sodium hydride dispersion in mineral oil and carbon disulfide) were purchased from Alfa‐Aesar and Sigma–Aldrich. DMF and THF were of reagent grade and were dried on molecular sieves (5 Å 1/16” inch pellets). Unless otherwise stated, all commercial reagents were used without further purification. Organic solutions were dried over anhydrous sodium sulfate. Thin‐layer chromatography (TLC) was carried out using aluminum‐backed silica gel plates (Merck DC‐Alufolien Kieselgel 60 F_254_). Neat DCM or DCM/2%–10% methanol mixture was used as a developing solvent, and detection of spots was made by UV light and/or by iodine vapors. Melting points were determined on a Fisher‐Johns apparatus and are uncorrected. ^1^H NMR and ^13^C NMR spectra were recorded on a Varian Gemini (200 MHz Palo Alto, California, USA) or JEOL JNMECZR (400 MHz, Tokyo, Japan); chemical shifts were reported in *δ* (ppm) units relative to the internal reference tetramethylsilane, and the splitting patterns were described as follows: s (singlet), bs (broad singlet), d (doublet), t (triplet), q (quartet), and m (multiplet). The first‐order values reported for coupling constants J were given in Hz. Elemental analyses were determined by an EA1110 Analyzer, Fison Instruments (Milan, Italy). IR spectra were recorded by FT‐IR Spectrum Two (Perkin‐Elmer, Waltham, MA, USA). Compounds **1** and **2a–c** were prepared as previously reported [38, 39].

#### 4.1.1 Synthetic Procedure for the Preparation of Ethyl 5‐Amino‐1‐Benzyl‐3‐(phenylamino)‐1H‐Pyrazole‐4‐Carboxylate 2d

In a sealed tube, a mixture of TEA (1 mL), **1** (803 mg, 3 mmol), and benzylhydrazine oxalate (650 mg, 3 mmol) was heated at 80 °C for 4 h. After cooling at rt, water (10 mL) was added, and the solution was acidified with HCl 2 M. The precipitated solid was collected by filtration and then purified by crystallization from EtOH.

Yield: 68%. White solid. Mp 135–137 °C (EtOH). ^1^H NMR (400 MHz, DMSO‐d_6_) *δ* 1.31 (t, 3H, *J* = 7.1 Hz, CH_3_), 4.26 (q, 2H, *J* = 7.1 Hz, CH_2_O), 5.13 (s, 2H, CH_2_N), 6.41 (bs, 2H, NH_2_, exchangeable), 6.77–6.83 (m, 1H, arom. H), 7.17–7.28 (m, 5H, arom. H), 7.30–7.36 (m, 2H, arom. H), 7.48–7.53 (m, 2H, arom. H), 8.08 (bs, 1H, NH, exchangeable). ^13^C NMR (101 MHz, DMSO‐d_6_): *δ* 164.00, 150.20, 148.94, 141.30, 137.32, 128.77, 128.44, 127.19, 127.03, 119.58, 116.22, 81.93, 58.96, 49.35, 14.53. Anal calcd for C_19_H_20_N_4_O_2_: C, 67.84; H, 5.99; N, 16.66. Found: C, 68.17; H, 6.12; N, 16.62.

#### 4.1.2 General Procedure for the Synthesis of Pyrazolyl Thioureas 3 and 4

To a dry toluene solution of pyrazole **2a** (250 mg, 1 mmol) or **2b** (268 mg, 1 mmol), the proper phenyl isothiocyanate (1 mmol) was added, and the reaction mixture was stirred at reflux for 6–8 h. After cooling at rt, the precipitated solid was collected by filtration and purified by column chromatography in silica gel using DCM as eluent (**3a–c**) or recrystallized from the proper solvent or solvent mixture (**4a–d**).

##### 4.1.2.1 *Ethyl 5‐*(*Phenylamino*)*‐3‐*(*3‐Phenylureido*)*‐1H‐Pyrazole‐4‐Carboxylate* (3a)

Yield: 55%. White solid. Mp 155–157 °C (Et_2_O). ^1^H NMR (400 MHz, DMSO‐d_6_) *δ* 1.36 (t, 3H, *J* = 7.0 Hz, CH_3_), 4.34 (q, 2H, *J* = 7.0 Hz, CH_2_O), 6.92–6.96 (m, 1H, arom. H), 7.26–7.31 (m, 2H, arom. H), 7.33–7.38 (m, 1H, arom. H), 7.43–7.50 (m, 4H, arom. H), 7,69–7.73 (m, 2H, arom. H), 8.46 (bs, 1H, NH phenyl, exchangeable), 11.01 (bs, 1H, NH thiourea, exchangeable). ^13^C NMR (101 MHz, DMSO‐d_6_) *δ* 174.34, 163.70, 153.23, 149.90, 139.67, 137.69, 129.01, 128.76, 127.45, 127.18, 121.21, 117.88, 83.62, 59.96, 14.44. Anal calcd for C_19_H_19_N_5_O_2_S: C, 59.83; H, 5.02; N, 18.36; S, 8.40. Found: C, 60.04; H, 4.83; N, 18.29; S, 8.25.

##### 4.1.2.2 *Ethyl 3‐*(*3‐*(*3‐Chlorophenyl*)*Thioureido*)*‐5‐*(*Phenylamino*)*‐1H‐Pyrazole‐4‐Carboxylate* (3b)

Yield: 40%. White solid. Mp 131–133 °C (EtOH). ^1^H NMR (400 MHz, DMSO‐d_6_) *δ* 1.32 (t, 3H, *J* = 7.1 Hz, CH_3_), 4.31 (q, 2H, *J* = 7.1 Hz, CH_2_O), 6.89–6.94 (m, 1H, arom. H), 7.23–7.29 (m, 2H, arom. H), 7.36–7.48 (m, 3H, arom. H), 7.54–7.57 (m, 1H, arom. H), 7.65–7.69 (m, 2H, arom. H), 8.42 (bs, 1H, NH phenyl, exchangeable), 10.98 (bs, 1H, NH thiourea, exchangeable). ^13^C NMR (101 MHz, DMSO‐d_6_) *δ* 174.41, 163.61, 153.24, 149.92, 139.60, 132.71, 131.37, 130.22, 128.96, 128.03, 127.30, 126.35, 121.22, 117.86, 83.66, 59.94, 14.38. Anal calcd for C_19_H_18_ClN_5_O_2_S: C, 54.87; H, 4.36; N, 16.84; S, 7.71. Found: C, 54.75; H, 4.18; N, 16.53; S, 7.53.

##### 4.1.2.3 *Ethyl 3‐*(*3‐*(*4‐Chlorophenyl*)*Thioureido*)*‐5‐*(*Phenylamino*)*‐1H‐Pyrazole‐4‐Carboxylate* (3c)

Yield: 48%. White solid. Mp 139–140 °C (EtOH). ^1^H NMR (400 MHz, DMSO‐d_6_) *δ* 1.32 (t, 3H, *J* = 7.1 Hz, CH_3_), 4.31 (q, 2H, *J* = 7.1 Hz, CH_2_O), 6.86–6.97 (m, 1H, arom. H), 7.21–7.28 (m, 2H, arom. H), 7.42–7.52 (m, 4H, arom. H), 7.63–7.72 (m, 2H, arom. H), 8.41 (bs, 1H, NH phenyl, exchangeable), 10.96 (bs, 1H, NH thiourea, exchangeable). ^13^C NMR (101 MHz, DMSO‐d_6_) *δ* 174.38, 163.62, 153.21, 149.92, 139.61, 129.90, 129.25, 128.96, 128.64, 127.77, 121.21, 117.85, 83.64, 59.94. 14.38. Anal calcd for C_19_H_18_ClN_5_O_2_S: C, 54.87; H, 4.36; N, 16.84; S, 7.71. Found: C, 54.83; H, 4.38; N, 16.73; S, 7.81.

##### 4.1.2.4 *Ethyl 1‐Methyl‐5‐*(*Phenylamino*)*‐3‐*(*3‐Phenylthioureido*)*‐1H‐Pyrazole‐4‐Carboxylate* (4a)

Yield: 49%. White solid. Mp 151–153 °C (Et_2_O/Ligroin). ^1^H NMR (400 MHz, CDCl_3_) *δ* 1.40 (t, 3H, *J* = 7.1 Hz, CH_3_), 3.37 (s, 3H, CH_3_N), 4.36 (q, 2H, *J* = 7.1 Hz, CH_2_O), 6.89–6.94 (m, 2H, arom. H), 7.06–7.12 (m, 1H, arom. H), 7.21–7.27 (m, 2H, arom. H), 7.29–7.42 (m, 4H, 3 arom. H + NH phenyl, exchangeable), 7.62–7.67 (m, 2H, arom. H), 9.27 (bs, 1H, NH thiourea, exchangeable), 11.52 (bs, 1H, NH thiourea, exchangeable). ^13^C NMR (101 MHz, CDCl_3_) *δ* 177.46, 163.24, 147.64, 146.69, 140.83, 138.90, 129.78, 128.87, 126.43, 124.99, 124.11, 120.13, 88.83, 60.96, 37.34, 14.50. Anal calcd for C_20_H_21_N_5_O_2_S: C, 60.74; H, 5.35; N, 17.71; S, 8.11. Found: C, 60.56; H, 5.22; N, 17.75; S, 7.94.

##### 4.1.2.5 *Ethyl 1‐Methyl‐5‐*(*Phenylamino*)*‐3‐*(*3‐*(*p‐Tolyl*)*Thioureido*)*‐1H‐Pyrazole‐4‐Carboxylate* (4b)

Yield: 51%. White solid. Mp 175–176 °C (Et_2_O/Ligroin). ^1^H NMR (400 MHz, DMSO‐d_6_) *δ* 0.91 (t, 3H, *J* = 7.1 Hz, CH_3_), 2.32 (s, 3H, CH_3_‐Ph), 3.62 (s, 3H, CH_3_N), 4.02 (q, 2H, *J* = 7.1 Hz, CH_2_O), 6.75–6.82 (m, 2H, arom. H), 6.82–6.90 (m, 1H, arom. H), 7.18–7.26 (m, 4H, arom. H), 7.51–7.58 (m, 2H, arom. H), 8.48 (bs, 1H, NH phenyl, exchangeable), 9.60 (bs, 1H, NH thiourea, exchangeable), 11.40 (bs, 1H, NH thiourea, exchangeable). ^13^C NMR (101 MHz, DMSO‐d_6_) *δ* 176.20, 162.74, 148.04, 143.43, 143.00, 136.04, 135.29, 129.09, 129.03, 124.30, 120.38, 115.85, 90.33, 59.95, 35.68, 20.61, 13.47. Anal calcd for C_21_H_23_N_5_O_2_S: C, 61.59; H, 5.66; N, 17.10; S, 7.83. Found: C, 61.64; H, 5.70; N, 16.90; S, 7.53.

##### 4.1.2.6 *Ethyl 3‐*(*3‐*(*3‐Chlorophenyl*)*Thioureido*)*‐1‐Methyl‐5‐*(*Phenylamino*)*‐1H‐Pyrazole‐4‐Carboxylate* (4c)

Yield: 69%. White solid. Mp 145–147 °C (DCM/EtOH). ^1^H NMR (400 MHz, DMSO‐d_6_) *δ* 0.88 (t, 3H, *J* = 7.1 Hz, CH_3_), 3.59 (s, 3H, NCH_3_), 3.99 (q, 2H, *J* = 7.1 Hz, CH_2_O), 6.71–6.77 (m, 2H, arom. H), 6.80–6.86 (m, 1H, arom. H), 7.14–7.21 (m, 2H, arom. H), 7.25–7.29 (m, 1H, arom. H), 7.37–7.43 (m, 1H, arom. H), 7.53–7.57 (m, 1H, arom. H), 7.90–7.93 (m, 1H, arom. H), 8.41 (bs, 1H, NH phenyl, exchangeable), 9.65 (bs, 1H, NH thiourea, exchangeable), 11.48 (bs, 1H, NH thiourea, exchangeable). ^13^C NMR (101 MHz, DMSO‐d_6_) *δ* 176.33, 162.64, 147.69, 143.38, 143.05, 140.02, 132.71, 130.23, 129.01, 125.65, 123.69, 122.89, 120.39, 115.85, 90.70, 59.95, 35.68, 13.45. Anal calcd for C_20_H_20_ClN_5_O_2_S: C, 55.88; H, 4.69; N, 16.29; S, 7.46. Found: C, 55.69; H, 5.02; N, 16.54; S, 7.61.

##### 4.1.2.7 *Ethyl 3‐*(*3‐*(*4‐Chlorophenyl*)*Thioureido*)*‐1‐Methyl‐5‐*(*Phenylamino*)*‐1H‐Pyrazole‐4‐Carboxylate* (4d)

Yield: 63%. White solid. Mp 174–176 °C (DCM/EtOH). ^1^H NMR (400 MHz, DMSO‐d_6_) *δ* 0.92 (t, 3H, *J* = 7.1 Hz, CH_3_), 3.62 (s, 3H, NCH_3_), 4.02 (q, 2H, *J* = 7.1 Hz, CH_2_O), 6.76–6.81 (m, 2H, arom. H), 6.84–6.89 (m, 1H, arom. H), 7.19–7.24 (m, 2H, arom. H), 7.44–7.50 (m, 2H, arom. H), 7.70–7.74 (m, 2H, arom. H), 8.45 (bs, 1H, NH phenyl, exchangeable), 9.67 (bs, 1H, NH thiourea, exchangeable), and 11.47 (bs, 1H, NH thiourea, exchangeable). ^13^C NMR (101 MHz, DMSO‐d_6_) *δ* 176.34, 162.66, 147.78, 143.38, 143.05, 137.54, 129.85, 129.01, 128.53, 125.98, 120.39, 115.85, 90.59, 59.94, 35.67, 13.45. Anal calcd for C_20_H_20_ClN_5_O_2_S: C, 55.88; H, 4.69; N, 16.29; S, 7.46. Found: C, 55.79; H, 4.58; N, 16.24; S, 7.15.

#### 4.1.3 General Procedure for the Synthesis of Isothiocyanate Intermediates 5

To a dry THF solution (15 mL) of the proper pyrazole **2c** or **2d** (3 mmol) cooled at 0 °C, 55% sodium hydride dispersion in mineral oil (357 mg, 7.5 mmol) was added. After stirring at 0 °C for 10 min, CS_2_ (1828 µL, 30 mmol) was added, and the mixture was heated at 40 °C for 3 h. After cooling at 0 °C, I_2_ (381 mg, 3 mmol) was added portionwise, and the reaction was stirred at 0 °C for 1 h. The solution was diluted with Et_2_O (30 mL), and the precipitated solid was removed by filtration. The filtrate was washed with HCl 1 M (3 × 10 mL) and water (1 × 10 mL), dried, and filtered. Evaporation in vacuo gave a residue that was purified by column chromatography (silica gel, eluent: DCM).

##### 4.1.3.1 *Ethyl 5‐Isothiocyanato‐1‐Methyl‐3‐*(*Phenylamino*)*‐1H‐Pyrazole‐4‐Carboxylate* (5a)

Yield: 27%. Orange oil. IR (ATR)  cm^−1^ 3340 (NH), 2034 (NCS), 1676 (C = O). Anal calcd for C_14_H_14_N_4_O_2_S: C, 55.62; H, 4.67; N, 18.53; S, 10.60. Found: C, 55.40; H, 4.73; N, 18.27; S, 10.47.

##### 4.1.3.2 *Ethyl 1‐Benzyl‐5‐Isothiocyanato‐3‐*(*Phenylamino*)*‐1H‐Pyrazole‐4‐Carboxylate* (5b)

Yield: 16%. Yellow oil. IR (ATR)  cm^−1^ 3338 (NH), 2036 (NCS), 1675 (C = O). Anal calcd for C_20_H_18_N_4_O_2_S: C, 63.47; H, 4.79; N, 14.80; S, 8.47. Found: C, 63.35; H, 4.80; N, 14.59; S, 8.28.

#### 4.1.4 General Synthetic Procedure for the Synthesis of Compounds 6 and 7

To a dry DMF solution (7 mL) of pyrazole isothiocyanates **5a** or **5b** (1 mmol), the proper aniline (1 mmol) was added, and the reaction mixture was stirred at rt for 1 h. The solution was diluted with water (20 mL) and acidified with HCl 2 M (pH = 5). The precipitated solid was collected by filtration and recrystallized from the proper solvent or solvent mixture.

##### 4.1.4.1 *Ethyl 1‐Methyl‐3‐*(*Phenylamino*)*‐5‐*(*3‐Phenylthioureido*)*‐1H‐Pyrazole‐4‐Carboxylate* (6a)

Yield: 64%. White solid. Mp 164–165 °C (EtOH). ^1^H NMR (400 MHz, DMSO‐d_6_) *δ* 1.27 (t, 3H, *J* = 7.1 Hz, CH_3_), 3.63 (s, 3H, NCH_3_), 4.23 (q, 2H, *J* = 7.1 Hz, CH_2_O), 6.83–6.89 (m, 1H, arom. H), 7.16–7.21 (m, 1H, arom. H), 7.24–7.30 (m, 2H, arom. H), 7.35–7.41 (m, 2H, arom. H), 7.49–7.53 (m, 2H, arom. H), 7.56–7.60 (m, 2H, arom. H), 8.20 (bs, 1H, NH phenyl, exchangeable), 9.36 (bs, 1H, NH thiourea, exchangeable), 10.34 (bs, 1H, NH thiourea, exchangeable). ^13^C NMR (101 MHz, DMSO‐d_6_) *δ* 181.03, 163.39, 150.96, 141.08, 138.86, 138.71, 128.88, 128.71, 125.20, 123.78, 119.97, 116.38, 93.31, 59.61, 35.52, 14.21. Anal calcd for C_20_H_21_N_5_O_2_S: C, 60.74; H, 5.35; N, 17.71; S: 8.11. Found: C, 60.59; H, 5.53; N, 17.77; S, 8.26.

##### 4.1.4.2 *Ethyl 1‐Methyl‐3‐*(*Phenylamino*)*‐5‐*(*3‐Phenylthioureido*)*‐1H‐Pyrazole‐4‐Carboxylate* (6b)

Yield: 71%. White solid. Mp 158–160 °C (EtOH). ^1^H NMR (400 MHz, DMSO‐d_6_) *δ* 1.26 (t, 3H, *J* = 7.1 Hz, CH_3_), 3.63 (s, 3H, NCH_3_), 4.22 (q, 2H, *J* = 7.1 Hz, CH_2_O), 6.83–6.90 (m, 1H, arom. H), 7.22–7.30 (m, 3H, arom. H), 7.37–7.45 (m, 2H, arom. H), 7.56–7.61 (m, 2H, arom. H), 7.71–7.74 (m, 1H, arom. H), 8.21 (bs, 1H, NH phenyl, exchangeable), 9.58 (bs, 1H, NH thiourea, exchangeable), 10.44 (bs, 1H, NH thiourea, exchangeable). ^13^C NMR (101 MHz, DMSO‐d_6_) *δ* 181.19, 163.31, 151.03, 141.05, 140.33, 138.51, 132.73, 130.31, 128.88, 124.78, 123.14, 122.08, 120.02, 116.41, 93.31, 59.67, 35.50, 14.20. Anal calcd for C_20_H_20_ClN_5_O_2_S: C, 55.88; H, 4.69; N, 16.29; S, 7.46. Found: C, 55.72; H, 4.83; N, 16.14; S, 7.63.

##### 4.1.4.3 *Ethyl 5‐*(*3‐*(*4‐Chlorophenyl*)*Thioureido*)*‐1‐Methyl‐3‐*(*Phenylamino*)*‐1H‐Pyrazole‐4‐Carboxylate* (6c)

Yield: 79%. White solid. Mp 154–156 °C (EtOH). ^1^H NMR (400 MHz, DMSO‐d_6_) *δ* 1.25 (t, 3H, *J* = 7.1 Hz, CH_3_), 3.62 (s, 3H, NCH_3_), 4.21 (q, 2H, *J* = 7.1 Hz, CH_2_O), 6.83–6.89 (m, 1H, arom. H), 7.24–7.30 (m, 2H, arom. H), 7.41–7.46 (m, 2H, arom. H), 7.52–7.61 (m, 4H, arom. H), 8.21 (bs, 1H, NH phenyl, exchangeable), 9.51 (bs, 1H, NH thiourea, exchangeable), 10.41 (bs, 1H, NH thiourea, exchangeable). ^13^C NMR (101 MHz, DMSO‐d_6_) *δ* 181.23, 163.38, 162.35, 151.04, 141.08, 137.82, 129.09, 128.94, 128.62, 125.49, 120.05, 116.42, 93.31, 59.70, 35.53, 14.26. Anal calcd for C_20_H_20_ClN_5_O_2_S: C, 55.88; H, 4.69; N, 16.29; S, 7.46. Found: C, 55.94; H, 4.84; N, 15.91; S, 7.12.

##### 4.1.4.4 *Ethyl 5‐*(*3‐*(*2*,*5‐Difluorophenyl*)*Thioureido*)*‐1‐Methyl‐3‐*(*Phenylamino*)*‐1H‐Pyrazole‐4‐Carboxylate* (6d)

Yield: 42%. White solid. Mp 152–155 °C (DCM/Cyclohexane). ^1^H NMR (400 MHz, DMSO‐d_6_) *δ* 1.23 (t, 3H, *J* = 7.1 Hz, CH_3_), 3.60 (s, 3H, NCH_3_), 4.19 (q, 2H, *J* = 7.1 Hz, CH_2_O), 6.79–6.86 (m, 1H, arom. H), 7.06–7.15 (m, 1H, arom. H), 7.20–7.26 (m, 2H, arom. H), 7.29–7.35 (m, 1H, arom. H), 7.52–7.58 (m, 2H, arom. H), 7.63–7.72 (m, 1H, arom. H), 8.16 (bs, 1H, NH phenyl, exchangeable), 9.69 (bs, 1H, NH thiourea, exchangeable), 10.12 (bs, 1H, NH thiourea, exchangeable). ^13^C NMR (101 MHz, DMSO‐d_6_) *δ* 181.99, 163.34, 158.54, 156.15, 151.00, 141.04, 128.89, 127.58, 120.03, 117.04, 116.72, 116.41, 116.18, 114.16, 93.37, 59.65, 35.50, 14.08. Anal calcd for C_20_H_19_F_2_N_5_O_2_S: C, 55.68; H, 4.44; N, 16.23; S, 7.43. Found: C, 55.66; H, 4.34; N, 16.31; S, 7.55.

##### 4.1.4.5 *Ethyl 5‐*(*3‐*(*4‐Chloro‐3‐Nitrophenyl*)*Thioureido*)*‐1‐Methyl‐3‐*(*Phenylamino*)*‐1H‐Pyrazole‐4‐Carboxylate* (6e)

Yield: 18%. White solid. Mp 175–177 °C (DCM/Cyclohexane). ^1^H NMR (400 MHz, DMSO‐d_6_) *δ* 1.20 (t, 3H, *J* = 7.1 Hz, CH_3_), 3.60 (s, 3H, NCH_3_), 4.17 (q, 2H, *J* = 7.1 Hz, CH_2_O), 6.80–6.86 (m, 1H, arom. H), 7.19–7.27 (m, 2H, arom. H), 7.49–7.58 (m, 2H, arom. H), 7.69–7.84 (m, 2H, arom. H), 8.13–8.19 (m, 1H, arom. H), 8.36 (bs, 1H, NH phenyl, exchangeable), 9.80 (bs, 1H, NH thiourea, exchangeable), 10.60 (bs, 1H, NH thiourea, exchangeable). ^13^C NMR (101 MHz, DMSO‐d_6_) *δ* 174.18, 163.22, 155.94, 151.09, 146.89, 141.02, 138.97, 138.21, 131.63, 128.89, 128.48, 120.07, 116.44, 114.89, 90.66, 59.71, 35.49, 14.20. Anal calcd for C_20_H_19_ClN_6_O_4_S: C, 50.58; H, 4.03; N, 17.70; S, 6.75. Found: C, 50.37; H, 3.97; N, 17.75; S, 6.54.

##### 4.1.4.6 *Ethyl 5‐*(*3‐*(*2*,*2‐Difluorobenzo*[*d* [*1*,*3*] *Dioxol‐5‐yl*)*Thioureido*)*‐1‐Methyl‐3‐*(*Phenylamino*)*‐1H‐Pyrazole‐4‐Carboxylate* (6f)

Yield: 76%. White solid. Mp 176–179 °C (DCM/EtOH). ^1^H NMR (400 MHz, DMSO‐d_6_) *δ* 1.28 (t, 3H, *J* = 7.1 Hz, CH_3_), 3.63 (s, 3H, NCH_3_), 4.24 (q, 2H, *J* = 7.1 Hz, CH_2_O), 6.84–6.89 (m, 1H, arom. H), 7.16–7.30 (m, 3H, arom. H), 7.41–7.46 (m, 1H, arom. H), 7.56–7.61 (m, 2H, arom. H), 7.64–7.68 (m, 1H, arom. H), 8.20 (bs, 1H, NH phenyl, exchangeable), 9.53 (bs, 1H, NH thiourea, exchangeable), 10.54 (bs, 1H, NH thiourea, exchangeable). ^13^C NMR (101 MHz, DMSO‐d_6_) *δ* 181.54, 163.32, 150.98, 142.43, 141.06, 140.13, 138.66, 135.17, 131.35, 128.89, 120.38, 120.02, 116.41, 109.99, 107.34, 93.35, 59.67, 35.52, 14.25. Anal calcd for C_21_H_19_F_2_N_5_O_4_S: C, 53.05; H, 4.03; N, 14.73; S, 6.74. Found: C, 53.14; H, 3.97; N, 14.58; S, 6.99.

##### 4.1.4.7 *Ethyl 1‐Benzyl‐3‐*(*Phenylamino*)*‐5‐*(*3‐Phenylthioureido*)*‐1H‐Pyrazole‐4‐Carboxylate* (7a)

Yield: 79%. White solid. Mp 172–175 °C (EtOH). ^1^H NMR (400 MHz, DMSO‐d_6_) *δ* 1.27 (t, *J* = 7.1 Hz, 3H, CH_3_), 4.23 (q, *J* = 7.1 Hz, 2H, CH_2_O), 5.17 (s, 2H, PhCH_2_), 6.81–6.87 (m, 1H, arom. H), 7.15–7.39 (m, 10H, arom. H), 7.45–7.49 (m, 2H, H arom. H), 7.51–7.55 (m, 2H, arom. H), 8.23 (bs, 1H, NH phenyl, exchangeable), 9.49 (bs, 1H, NH thiourea, exchangeable), 10.26 (bs, 1H, NH thiourea, exchangeable). ^13^C NMR (101 MHz, DMSO‐d_6_) *δ* 181.33, 163.45, 151.27, 141.02, 138.74, 136.38, 128.86, 128.64, 128.36, 127.86, 127.52, 125.23, 123.97, 119.99, 116.34, 93.89, 59.68, 51.31, 14.18. Anal calcd for C_26_H_25_N_5_O_2_S: C, 66.22; H, 5.34; N, 14.85; S, 6.80. Found: C, 66.12; H, 5.43; N, 14.57; S, 7.10.

##### 4.1.4.8 *Ethyl 1‐Benzyl‐5‐*(*3‐*(*3‐Chlorophenyl*)*Thioureido*)*‐3‐*(*Phenylamino*)*‐1H‐Pyrazole‐4‐Carboxylate* (7b)

Yield: 39%. White solid. Mp 164–166 °C (EtOH). ^1^H NMR (400 MHz, DMSO‐d_6_) *δ* 1.26 (t, *J* = 7.1 Hz, 3H, CH_3_), 4.23 (q, *J* = 7.1 Hz, 2H, CH_2_O), 5.17 (s, 2H, PhCH_2_), 6.82–6.88 (m, 1H, arom. H), 7.20–7.41 (m, 10H, arom. H), 7.51–7.57 (m, 2H, H arom. H), 7.65–7.71 (m, 1H, arom. H), 8.23 (bs, 1H, NH phenyl, exchangeable), 9.70 (bs, 1H, NH thiourea, exchangeable), 10.36 (bs, 1H, NH thiourea, exchangeable). ^13^C NMR (101 MHz, DMSO‐d_6_) *δ* 181.68, 163.58, 151.52, 141.20, 140.52, 136.51, 132.87, 130.43, 129.07, 128.57, 128.10, 127.76, 125.06, 123.62, 122.55, 120.24, 116.58, 94.13, 59.92, 51.49, 14.38. Anal calcd for C_26_H_24_ClN_5_O_2_S: C, 61.71; H, 4.78; N, 13.84; S, 6.34. Found: C, 62.09; H, 5.00; N, 13.55; S, 6.12.

##### 4.1.4.9 *Ethyl 1‐Benzyl‐5‐*(*3‐*(*4‐Chlorophenyl*)*Thioureido*)*‐3‐*(*Phenylamino*)*‐1H‐Pyrazole‐4‐Carboxylate* (7c)

Yield: 78%. White solid. Mp 191–193 °C (EtOH). ^1^H NMR (400 MHz, DMSO‐d_6_) *δ* 1.25 (t, 3H, *J* = 7.1 Hz, CH_3_), 4.22 (q, 2H, *J* = 7.1 Hz, CH_2_O), 5.17 (s, 2H, PhCH_2_), 6.81–6.87 (m, 1H, arom. H), 7.21–7.26 (m, 2H, arom. H), 7.27–7.31 (m, 1H, arom. H), 7.33–7.36 (m, 4H, arom. H), 7.39–7.44 (m, 2H, arom. H), 7.47–7.56 (m, 4H, arom. H), 8.24 (bs, 1H, NH phenyl, exchangeable), 9.63 (bs, 1H, NH thiourea, exchangeable), 10.31 (bs, 1H, NH thiourea, exchangeable). ^13^C NMR (101 MHz, DMSO‐d_6_) *δ* 181.73, 163.65, 151.55, 141.23, 138.04, 136.58, 129.36, 129.12, 128.74, 128.61, 128.11, 127.79, 125.94, 120.26, 116.59, 94.14, 59.95, 51.48, 14.43. Anal calcd for C_26_H_24_ClN_5_O_2_S: C, 61.71; H, 4.78; N, 13.84; S, 6.34. Found: C, 62.07; H, 4.87; N, 13.61; S, 6.18.

##### 4.1.4.10 *Ethyl 1‐Benzyl‐5‐*(*3‐*(*2*,*5‐Difluorophenyl*)*Thioureido*)*‐3‐*(*Phenylamino*)*‐1H‐Pyrazole‐4‐Carboxylate* (7d)

Yield: 21%. Yellow solid. Mp 147–149 °C (DCM/Cyclohexane). ^1^H NMR (400 MHz, DMSO‐d_6_) *δ* 1.27 (t, *J* = 7.1 Hz, 3H, CH_3_), 4.23 (q, *J* = 7.1, 2H, CH_2_O), 5.18 (s, 2H, PhCH_2_), 6.80–6.87 (m, 1H, arom. H), 7.10–7.18 (m, 1H, arom. H), 7.20–7.39 (m, 9H, arom. H), 7.52–7.56 (m, 2H, arom. H), 8.23 (bs, 1H, NH phenyl, exchangeable), 9.86 (bs, 1H, NH thiourea, exchangeable), 10.08 (bs, 1H, NH thiourea, exchangeable). ^13^C NMR (101 MHz, DMSO‐d_6_) *δ* 182.25, 163.39, 158.50, 156.11, 151.32, 140.98, 136.32, 128.88, 128.43, 127.84, 127.61, 120.07, 116.98, 116.89, 116.75, 116.66, 116.38, 113.88, 93.95, 59.73, 51.34, 14.05. Anal calcd for C_26_H_23_F_2_N_5_O_2_S: C, 61.53; H, 4.57; N, 13.80; S, 6.32. Found: C, 61.33; H, 4.61; N, 13.65; S, 6.12.

##### 4.1.4.11 *Ethyl 1‐Benzyl‐5‐*(*3‐*(*4‐Chloro‐3‐Nitrophenyl*)*Thioureido*)*‐3‐*(*Phenylamino*)*‐1H‐Pyrazole‐4‐Carboxylate* (7e)

Yield: 63%. Yellow solid. Mp 112–115 °C (DCM/Cyclohexane). ^1^H NMR (400 MHz, DMSO‐d_6_) *δ* 1.24 (t, *J* = 7.1 Hz, 3H, CH_3_), 4.22 (q, *J* = 7.1 Hz, 2H, CH_2_O), 5.17 (s, 2H, PhCH_2_), 6.82–6.87 (m, 1H, arom. H), 7.20–7.37 (m, 7H, arom. H), 7.52–7.57 (m, 2H, arom. H), 7.73–7.82 (m, 3H, arom. H), 8.24 (bs, 1H, NH phenyl, exchangeable), 9.95 (bs, 1H, NH thiourea, exchangeable), 10.57 (bs, 1H, NH thiourea, exchangeable). ^13^C NMR (101 MHz, DMSO‐d_6_) *δ* 181.75, 163.28, 162.32, 151.37, 146.87, 140.97, 139.02, 136.23, 131.58, 128.90, 128.82, 128.39, 127.95, 127.61, 120.49, 120.10, 116.41, 94.00, 59.78, 51.28, 14.19. Anal calcd for C_26_H_23_ClN_6_O_4_S: C, 56.67; H, 4.21; N, 15.25; S, 5.82. Found: C, 56.79; H, 3.97; N, 14.98; S, 5.91.

##### 4.1.4.12 *Ethyl 1‐Benzyl‐5‐*(*3‐*(*2*,*2‐Difluorobenzo*[*d* [*1*,*3*] *Dioxol‐5‐yl*)*Thioureido*)*‐3‐*(*Phenylamino*)*‐1H‐Pyrazole‐4‐Carboxylate* (7f)

Yield: 36%. White solid. Mp 153–156 °C (DCM/EtOH). ^1^H NMR(400 MHz, DMSO‐d_6_) *δ* 1.27 (t, *J* = 7.1 Hz, 3H, CH_3_), 4.24 (q, *J* = 7.1 Hz, 2H, CH_2_O), 5.17 (s, 2H, PhCH_2_), 6.81–6.87 (m, 1H, arom. H), 7.11–7.16 (m, 1H, arom. H), 7.20–7.45 (m, 8H, H arom), 7.50–7.57 (m, 3H, arom. H), 8.22 (bs, 1H, NH phenyl, exchangeable), 9.60 (bs, 1H, NH thiourea, exchangeable), 10.37 (bs, 1H, NH thiourea, exchangeable). ^13^C NMR (101 MHz, DMSO‐d6) *δ* 182.33, 163.91, 151.79, 142.92, 141.54, 140.76, 136.87, 135.64, 131.88, 129.41, 128.90, 128.46, 128.10, 121.27, 120.56, 116.89, 110.46, 108.22, 94.45, 60.27, 51.84, 14.78. Anal calcd for C_27_H_23_F_2_N_5_O_4_S: C, 58.80; H, 4.20; N, 12.70; S, 5.81. Found: C, 58.66; H, 4.13; N, 13.05; S, 5.87.

### 4.2 Biology

#### 4.2.1 MTT Assays

3‐(4,5‐dimethyl‐2‐thiazolyl)‐2,5‐diphenyl‐2H‐tetrazolium bromide (MTT) assays were performed using MCF‐7 (breast adenocarcinoma), Hep‐G2 (hepatocellular carcinoma), SK‐MEL28 (skin melanoma), MDA‐MB231 (breast adenocarcinoma), HeLa (cervical adenocarcinoma), SK‐BR3 (breast adenocarcinoma), A549 (lung carcinoma), and GM‐6114 (embryonic human fibroblast) cells. All tumor cell lines were obtained from Biologic Bank and Cell Factory, IRCCS Azienda Ospedaliera Metropolitana, Genoa, Italy, while the GM‐6114 cell line was obtained from ATCC, Manassas, VA. Cell culture protocol and compound testing procedure have been previously reported [48]. The results are expressed as a percentage of the control samples (100%) in which the cell lines were incubated with the same amount of solvent but without any compounds. The assay was repeated three times. In each set, every single compound was tested six times. Means and standard deviations were calculated.

#### 4.2.2 Enzymatic Assays

The synthesis of acetylated peptide H3K9 (H3K9Ac) and the evaluation of SIRT2 and SIRT1 deacetylase activity were performed as previously described [49]. The recombinant SIRT1 was produced in house: SIRT1.1 was a gift from Prof. John Denu (Addgene plasmid #13735; http://n2t.net/addgene:13735; RRID: Addgene_13735) [50].

#### 4.2.3 Western Blotting

HepG2 cells were treated with the putative SIRT2 inhibitors at selected concentrations or the respective amount of DMSO vehicle. After 36 h, cells were lysed, and the level of acetylated α‐tubulin was quantified by Western blot analysis, as previously described [51], using the following antibodies: clone 11H10 (Cell Signaling, #2125, Lot 11) for α‐tubulin, clone D20G3 (Cell Signaling, #5335, Lot 5) for acetylated α‐tubulin, and mouse anti‐rabbit IgG‐HRP (Santa Cruz Biotechnology, sc‐2357, Lot B1125) as secondary antibody.

### 4.3 Docking Calculations

Molecular docking calculations on SIRT1 and 2 proteins were carried out relying on the experimental data, PDB code 4I5I and 9FRU, respectively. **6a** and **7f** were built in silico by the MOE Builder tool included in the MOE2019.01 software [Molecular Operating Environment (MOE) | MOEsaic | PSILO, https://www.chemcomp.com/en/Products.htm (accessed 10 November 2025)]. Compounds have been parameterized with the AM1 partial charge calculation method. The energy minimization step has been performed by the Energy Minimize tool using the MMFF94x forcefield, included in MOE. The RMS (root mean square) gradient was equal to 0.0001, being the root mean square gradient the norm of the gradient times the square root of the number of (unfixed) atoms. All the molecular docking simulations at the 9FRU and 4I5I proteins have been performed by means of the DOCK tool included in Flare software [Flare, Pro version, Cresset, Litlington, Cambridgeshire, UK; https://cresset‐group.com/flare/], based on Lead Finder options. Lead Finder assumes that the protein is rigid and analyses the possible conformations of the ligand by rotating functional groups along each freely rotatable bond. The docking engine in Lead Finder combines a genetic algorithm search with local optimization procedures, which make Lead Finder efficient in coarse sampling of ligand poses and following refinement of promising solutions. Among the available scoring functions, dG prediction has been applied [52]. This scoring function has been designed to perform an accurate estimation of the free energy of protein‐ligand binding for a given protein–ligand complex. The scaling coefficients for this function have been derived by fitting calculated binding energies to the experimental values for a set of 100 protein–ligand complexes with known 3D structure and experimentally measured binding constants. Ligand docking involves a search for the global minimum on the multidimensional surface describing the free energy of the protein–ligand binding. Among five docking regimes implemented in Lead Finder, the “normal” one was exploited. The normal regimen uses the default Lead Finder docking algorithm, optimized to provide an accurate and exhaustive search algorithm. A validation of Lead Finder protein‐ligand docking against a set of 407 protein–ligand complexes is available [53]. For the two compounds, 10 conformations have been proposed, and the top scored ones were analyzed by visual inspection.

## Funding

This study was supported by the Università degli Studi di Genova (FRA 2024), Ministero dell'Università e della Ricerca (#2022P9RM9M), and Ministero della Salute (Ricerca corrente 2025).

## Conflicts of Interest

The authors declare no conflict of interest.

## Supporting information

Supplementary Material

## Data Availability

The data that support the findings of this study are available from the corresponding author upon reasonable request.
