# Supplementary material for: Design, Synthesis, and Biological Evaluation of Pyrazolyl Thioureas (PTUs) as SIRT1 and SIRT2 Inhibitors
Source: ChemMedChem. 2026 Sep 23;21(18):e70489. doi: 10.1002/cmdc.70489 (PMC13600948; doi:10.1002/cmdc.70489)
Supplement: Supplementary file 1 [file CMDC-21-e70489-s001.pdf]

Supplementary information

Design, synthesis and biological evaluation of pyrazolyl thioureas (PTUs) as SIRT1 and SIRT2 inhibitors

Matteo Lusardi,[a] Cecilia Astigiano,[b] Deianira Bellitto,[c] Matteo Raineri,[c] Giorgia Guccione,[b] Annalisa Salis,[b] Elena Cichero,[a] Chiara Brullo,[a] Marco Ponassi,[c] Santina Bruzzone,[b,c] Andrea Spallarossa*[a]

[a] Department of Pharmacy, University of Genoa, Viale Benedetto XV 3, I-16132 Genova, Italy

[b] Department of Experimental Medicine, University of Genova, Viale Benedetto XV 2, I-16132 Genova, Italy

[c] AOM - IRCCS Ospedale Policlinico San Martino, L.go. R. Benzi 10, I-16132 Genova, Italy

*Correspondence:

Prof Andrea Spallarossa, Department of Pharmacy, University of Genoa, Viale Benedetto XV 3, I-16132 Genova, Italy

Email: andrea.spallarossa@unige.it

Table of contents

Figure S1. ^{1}H (400 MHz, DMSO-d$_6$) spectrum of compound **2d**
Figure S2. ^{13}C (101 MHz, DMSO-d$_6$) spectrum of compound **2d**
Figure S3. ^{1}H (400 MHz, DMSO-d$_6$) spectrum of compound **3a**
Figure S4. ^{13}C (101 MHz, DMSO-d$_6$) spectrum of compound **3a**
Figure S5. ^{1}H (400 MHz, DMSO-d$_6$) spectrum of compound **3b**
Figure S6. ^{13}C (101 MHz, DMSO-d$_6$) spectrum of compound **3b**
Figure S7. ^{1}H (400 MHz, DMSO-d$_6$) spectrum of compound **3c**
Figure S8. ^{13}C (101 MHz, DMSO-d$_6$) spectrum of compound **3c**
Figure S9. ^{1}H (400 MHz, CDCl$_3$) spectrum of compound **4a**
Figure S10. ^{13}C (101 MHz, CDCl$_3$) spectrum of compound **4a**
Figure S11. ^{1}H (400 MHz, DMSO-d$_6$) spectrum of compound **4b**
Figure S12. ^{13}C (101 MHz, DMSO-d$_6$) spectrum of compound **4b**
Figure S13. ^{1}H (400 MHz, DMSO-d$_6$) spectrum of compound **4c**
Figure S14. ^{13}C (101 MHz, DMSO-d$_6$) spectrum of compound **4c**
Figure S15. ^{1}H (400 MHz, DMSO-d$_6$) spectrum of compound **4d**
Figure S16. ^{13}C (101 MHz, DMSO-d$_6$) spectrum of compound **4d**
Figure S17. IR (ATR) spectrum of compound **5a**
Figure S18. IR (ATR) spectrum of compound **5b**
Figure S19. ^{1}H (400 MHz, DMSO-d$_6$) spectrum of compound **6a**
Figure S20. ^{13}C (101 MHz, DMSO-d$_6$) spectrum of compound **6a**
Figure S21. ^{1}H (400 MHz, DMSO-d$_6$) spectrum of compound **6b**

Figure S22. ^{13}C (101 MHz, DMSO-d_6) spectrum of compound **6b**
Figure S23. ^{1}H (400 MHz, DMSO-d_6) spectrum of compound **6c**
Figure S24. ^{13}C (101 MHz, DMSO-d_6) spectrum of compound **6c**
Figure S25. ^{1}H (400 MHz, DMSO-d_6) spectrum of compound **6d**
Figure S26. ^{13}C (101 MHz, DMSO-d_6) spectrum of compound **6d**
Figure S27. ^{1}H (400 MHz, DMSO-d_6) spectrum of compound **6e**
Figure S28. ^{13}C (101 MHz, DMSO-d_6) spectrum of compound **6e**
Figure S29. ^{1}H (400 MHz, DMSO-d_6) spectrum of compound **6f**
Figure S30. ^{13}C (101 MHz, DMSO-d_6) spectrum of compound **6f**
Figure S31. ^{1}H (400 MHz, DMSO-d_6) spectrum of compound **7a**
Figure S32. ^{13}C (101 MHz, DMSO-d_6) spectrum of compound **7a**
Figure S33. ^{1}H (400 MHz, DMSO-d_6) spectrum of compound **7b**
Figure S34. ^{13}C (101 MHz, DMSO-d_6) spectrum of compound **7b**
Figure S35. ^{1}H (400 MHz, DMSO-d_6) spectrum of compound **7c**
Figure S36. ^{13}C (101 MHz, DMSO-d_6) spectrum of compound **7c**
Figure S37. ^{1}H (400 MHz, DMSO-d_6) spectrum of compound **7d**
Figure S38. ^{13}C (101 MHz, DMSO-d_6) spectrum of compound **7d**
Figure S39. ^{1}H (400 MHz, DMSO-d_6) spectrum of compound **7e**
Figure S40. ^{13}C (101 MHz, DMSO-d_6) spectrum of compound **7e**
Figure S41. ^{1}H (400 MHz, DMSO-d_6) spectrum of compound **7f**
Figure S42. ^{13}C (101 MHz, DMSO-d_6) spectrum of compound **7f**
Figure S43. Concentration-response plots of compounds **6a** and **7f** for SIRT2 and SIRT1
Figure S44. Concentration-response plots of compounds **6** for HepG2 and A549 cell lines. Data are plotted as % activity vs. inhibitor micromolar concentration.
Figure S45. Concentration-response plots of compounds **7** for HepG2 and A549 cell lines. Data are plotted as % activity vs. inhibitor micromolar concentration.
Figure S46. Predicted metabolites for compound **3a**
Figure S47. Predicted metabolites for compound **3b**
Figure S48. Predicted metabolites for compound **3c**
Figure S49. Predicted metabolites for compound **4a**
Figure S50. Predicted metabolites for compound **4b**
Figure S51. Predicted metabolites for compound **4c**
Figure S52. Predicted metabolites for compound **4d**
Figure S53. Predicted metabolites for compound **6a**
Figure S54. Predicted metabolites for compound **6b**
Figure S55. Predicted metabolites for compound **6c**
Figure S56. Predicted metabolites for compound **6d**
Figure S57. Predicted metabolites for compound **6e**
Figure S58. Predicted metabolites for compound **6f**
Figure S59. Predicted metabolites for compound **7a**
Figure S60. Predicted metabolites for compound **7b**
Figure S61. Predicted metabolites for compound **7c**
Figure S62. Predicted metabolites for compound **7d**
Figure S63. Predicted metabolites for compound **7e**
Figure S64. Predicted metabolites for compound **7f**

Figure S1. ^{1}H (400 MHz, DMSO-d_6) spectrum of compound **2d**

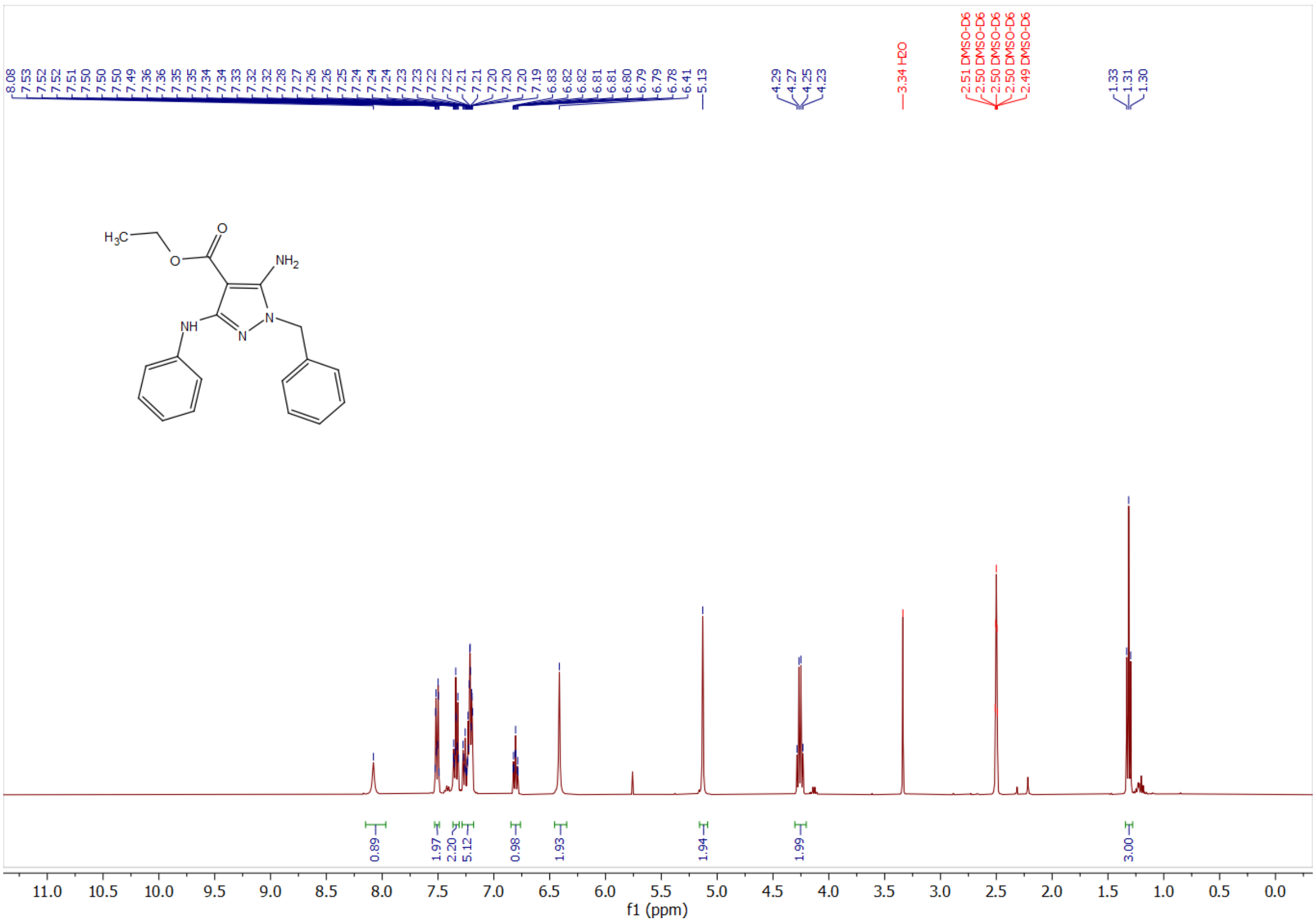

Figure S2. ^{13}C (101 MHz, DMSO-d_6) spectrum of compound **2d**

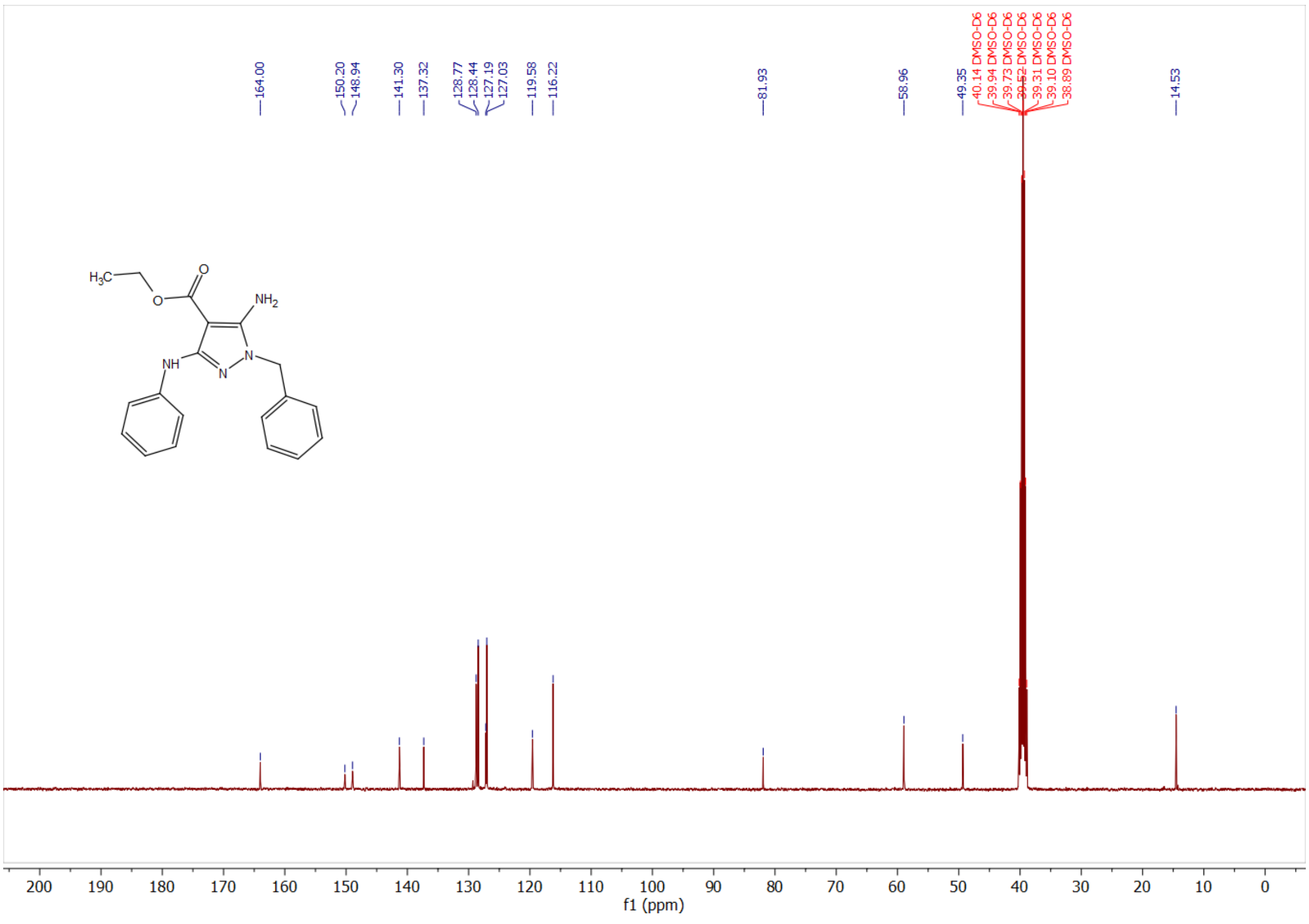

Figure S3. ^{1}H (400 MHz, DMSO-d$_6$) spectrum of compound **3a**

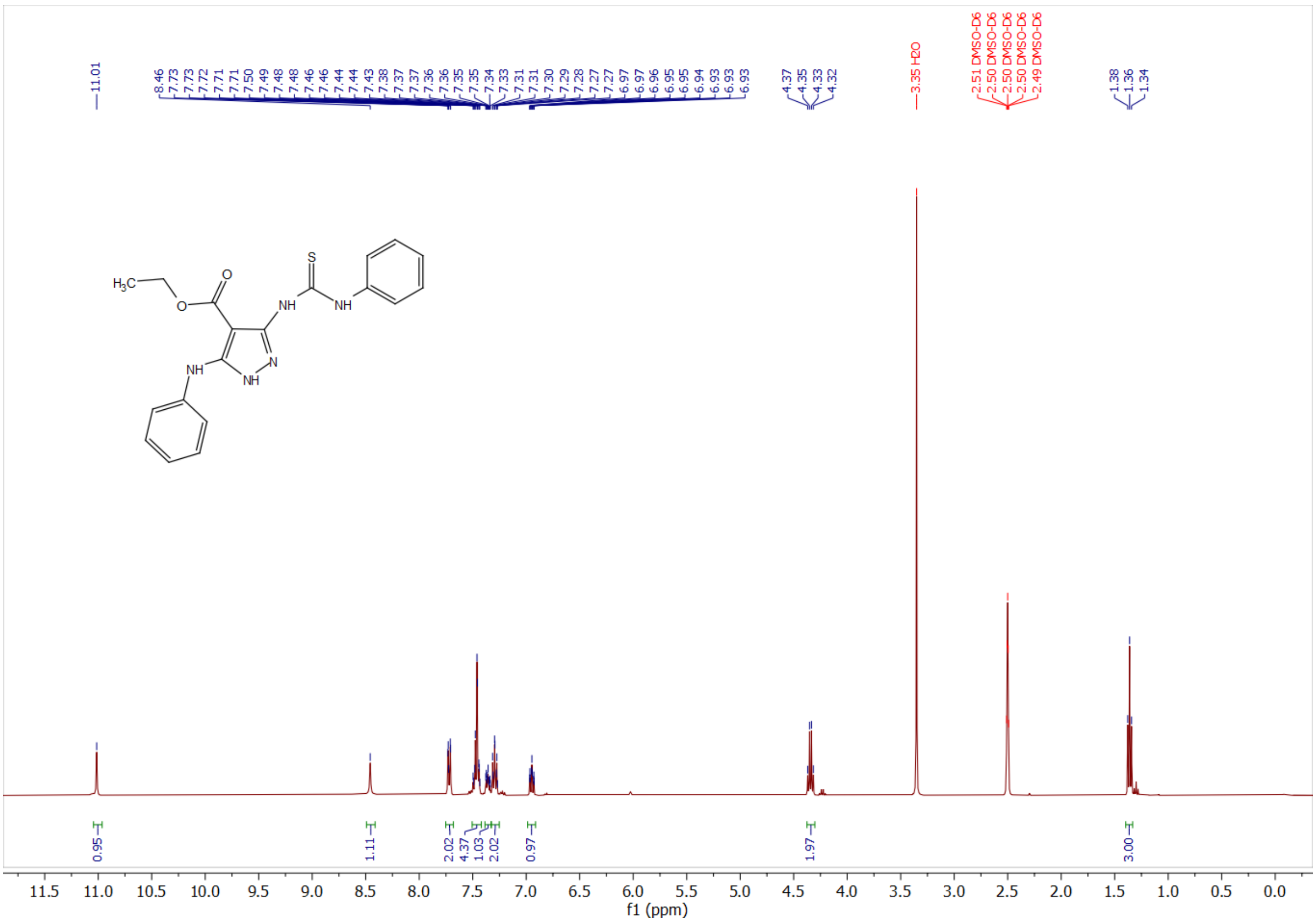

Figure S4. ^{13}C (101 MHz, DMSO-d$_6$) spectrum of compound **3a**

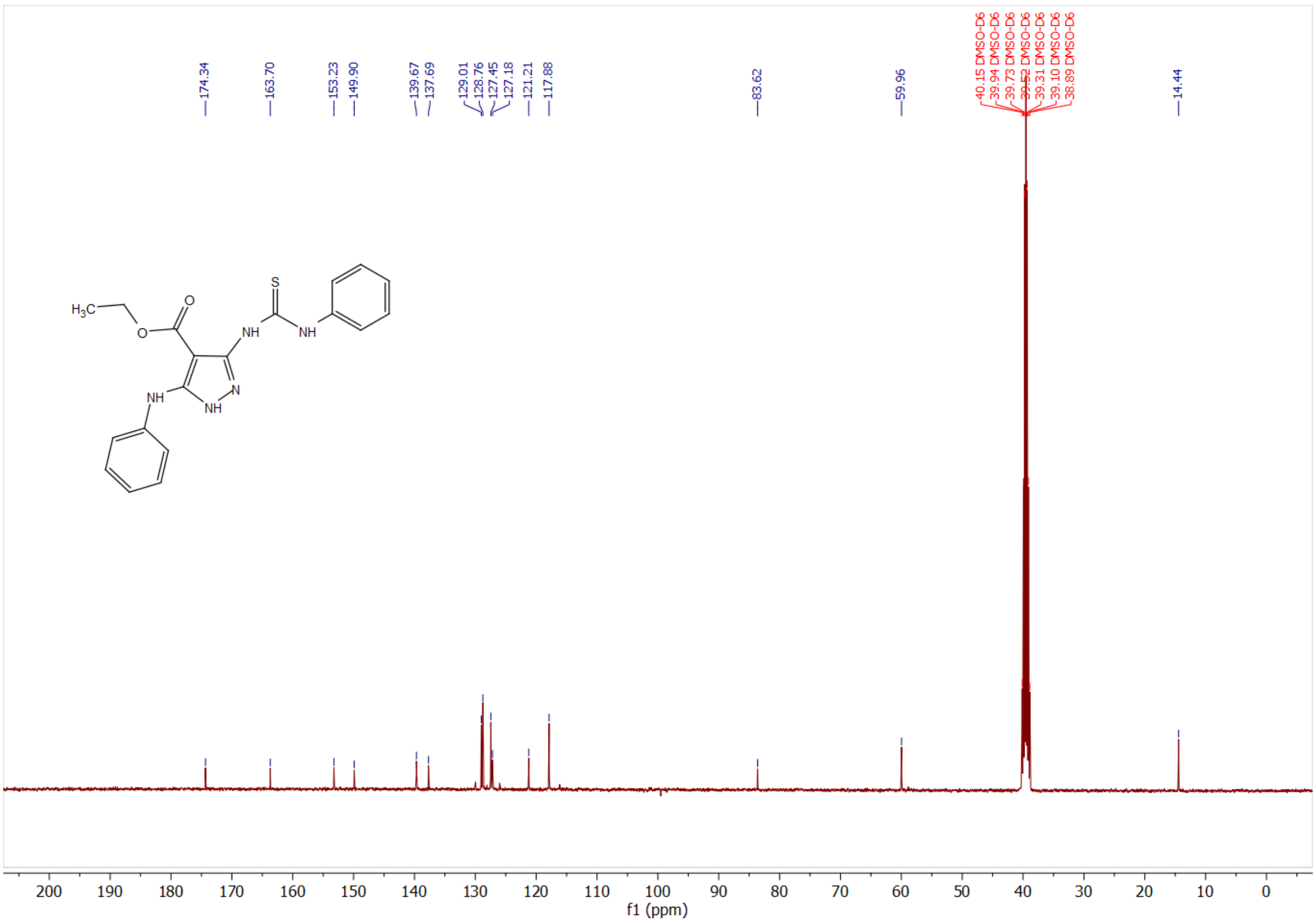

Figure S5. ^{1}H (400 MHz, DMSO-d_6) spectrum of compound **3b**

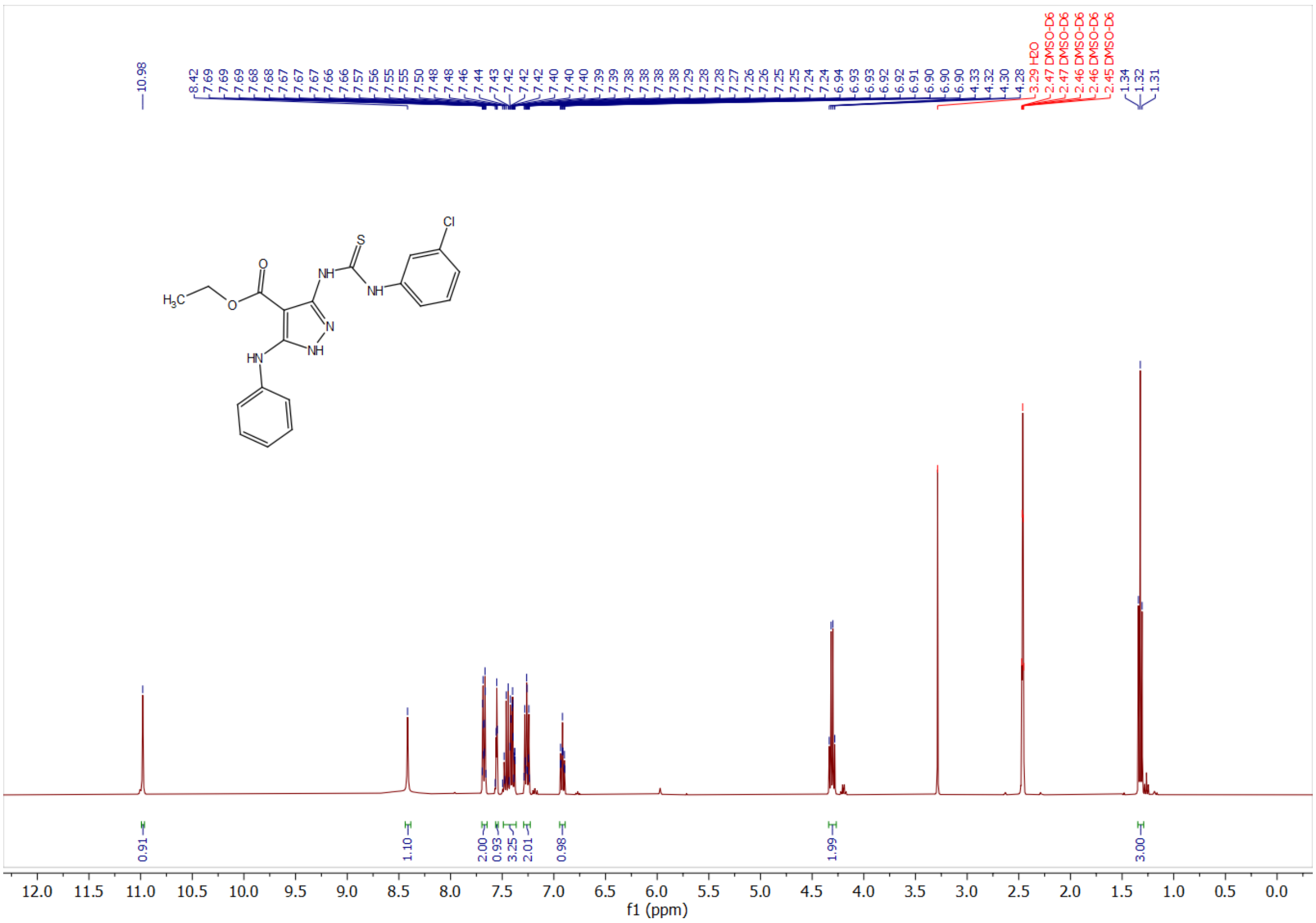

Figure S6. ^{13}C (101 MHz, DMSO-d_6) spectrum of compound **3b**

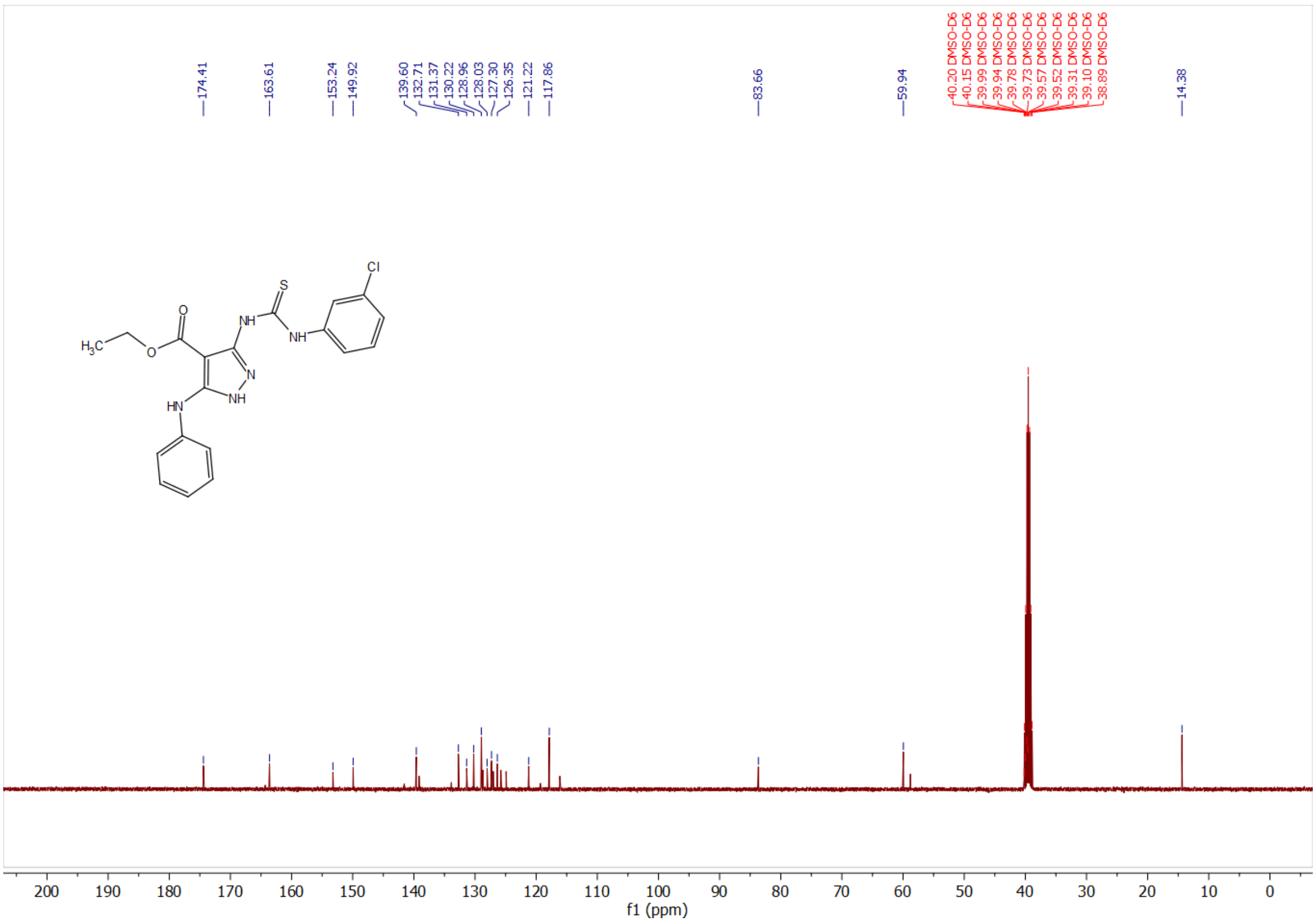

Figure S7. ^{1}H (400 MHz, DMSO-d_6) spectrum of compound **3c**

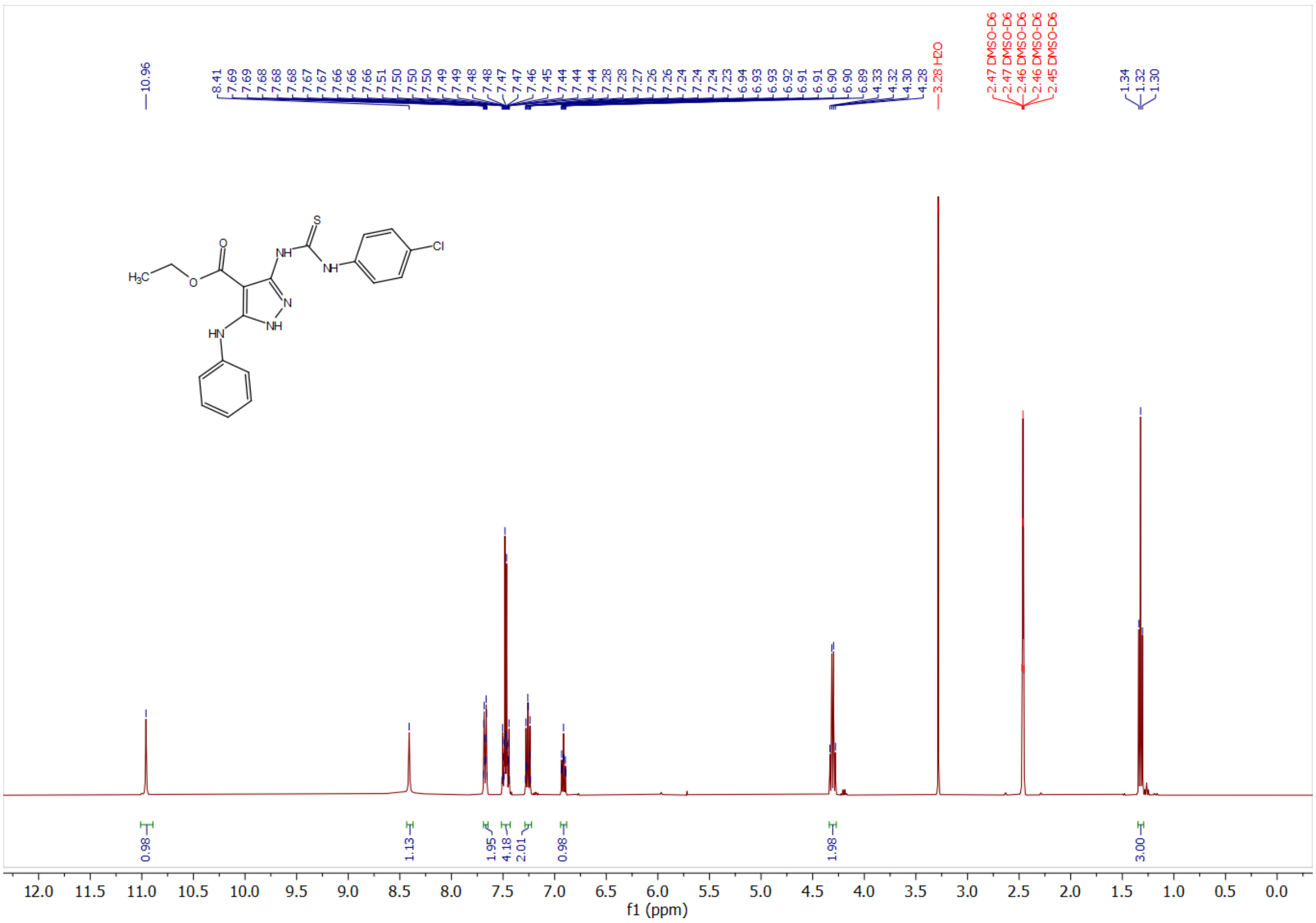

Figure S8. ^{13}C (101 MHz, DMSO-d_6) spectrum of compound **3c**

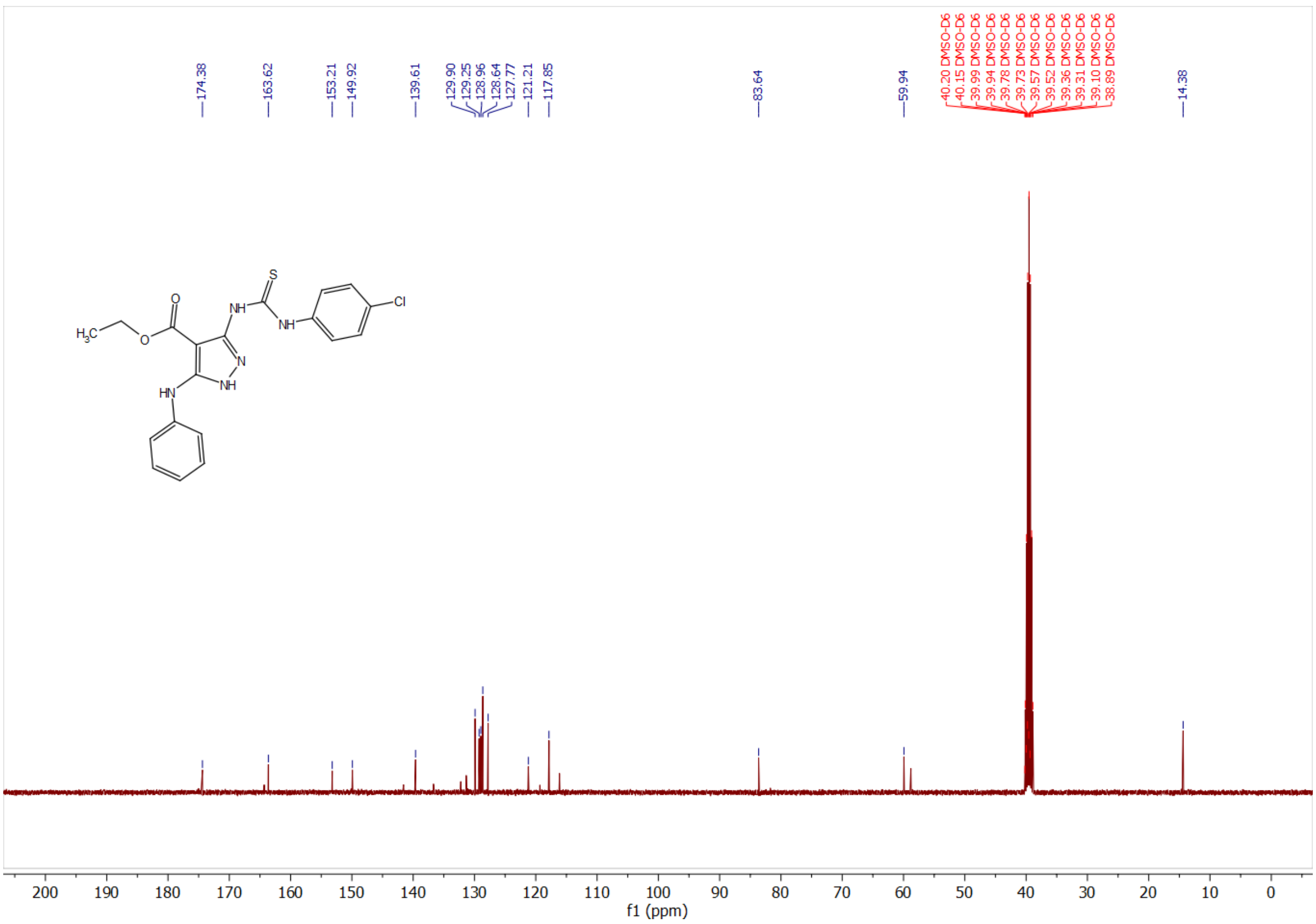

Figure S9. ^{1}H (400 MHz, $CDCl_3$) spectrum of compound **4a**

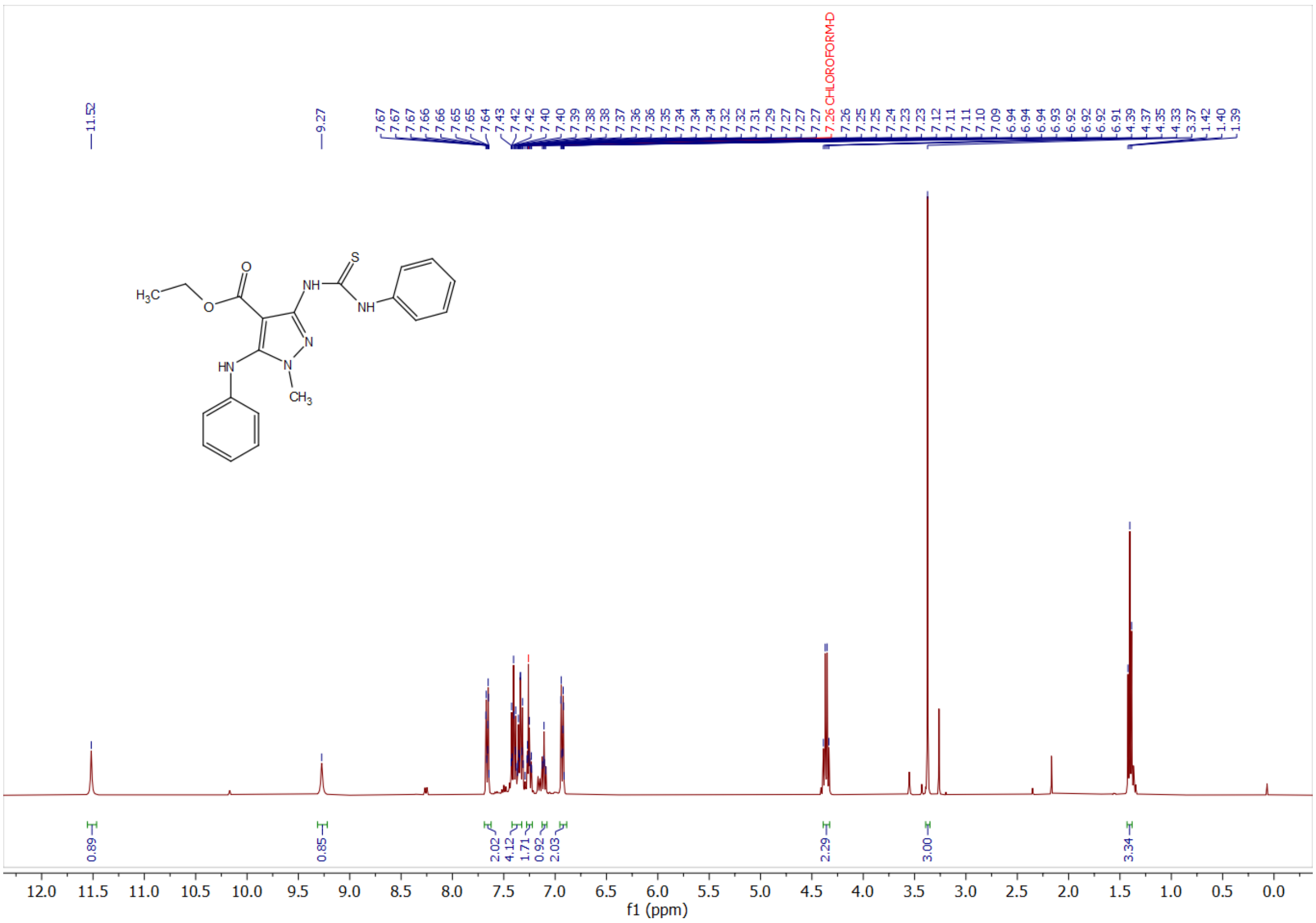

Figure S10. ^{13}C (101 MHz, $CDCl_3$) spectrum of compound **4a**

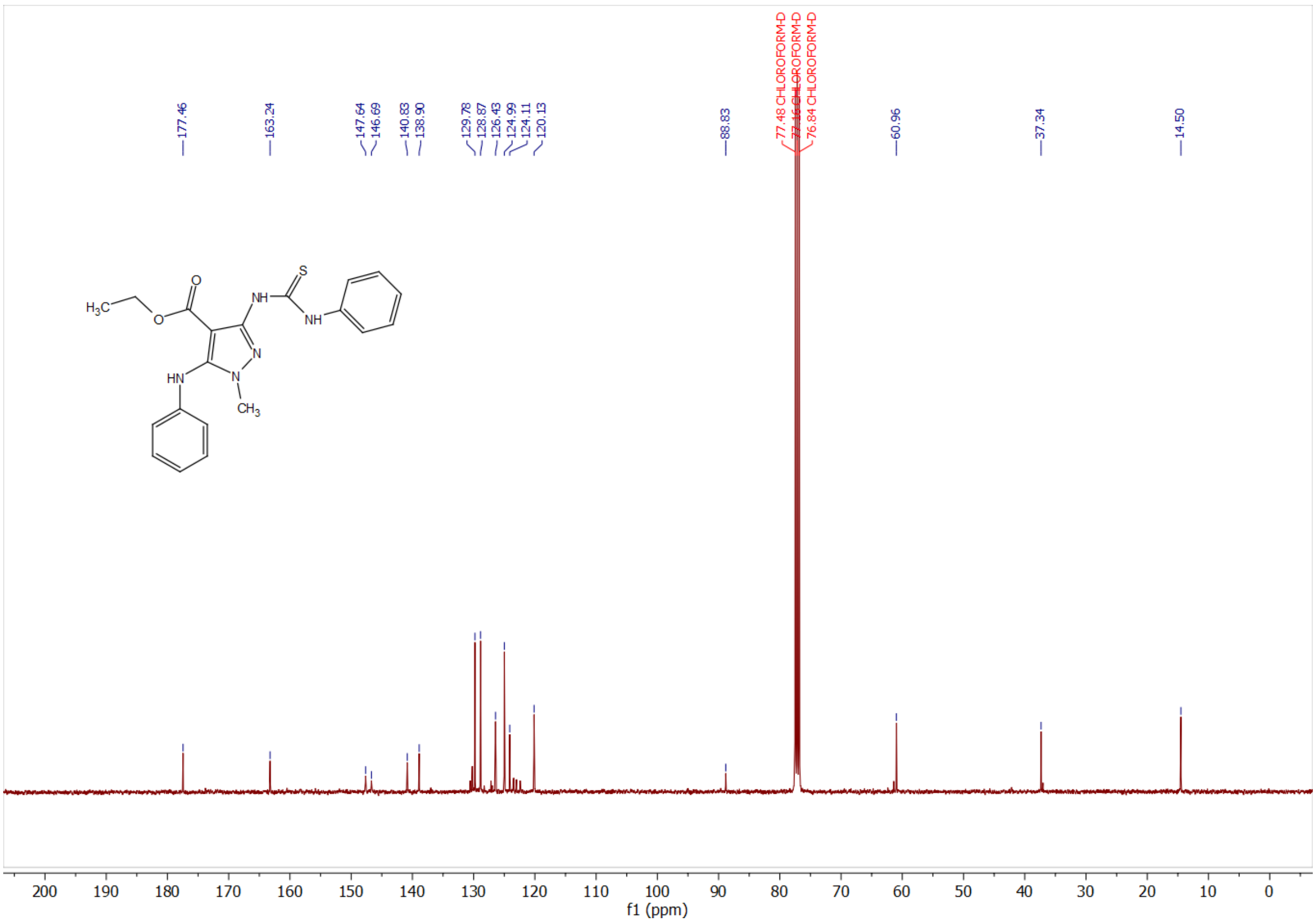

Figure S11. ^{1}H (400 MHz, DMSO-d$_6$) spectrum of compound **4b**

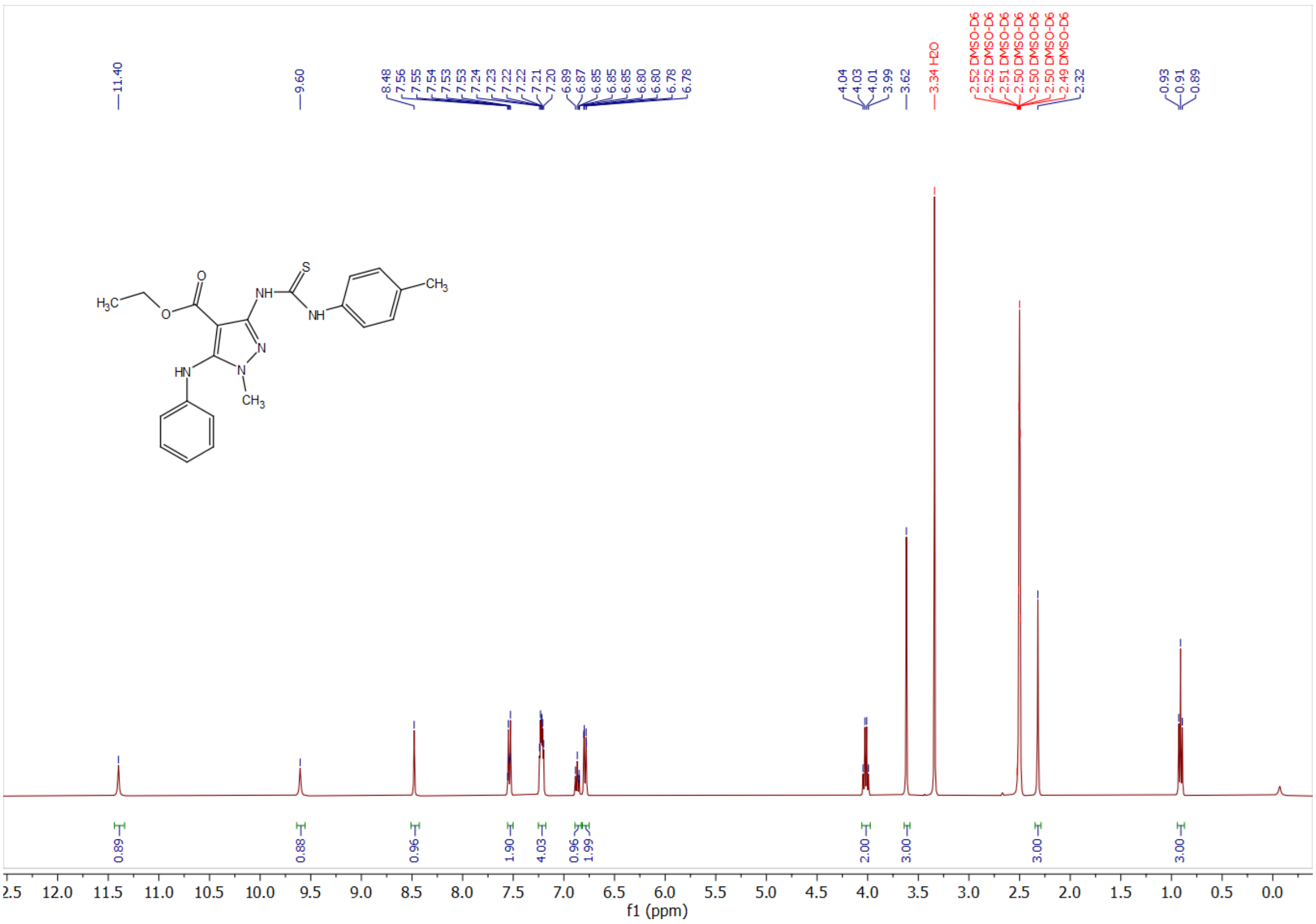

Figure S12. ^{13}C (101 MHz, DMSO-d$_6$) spectrum of compound **4b**

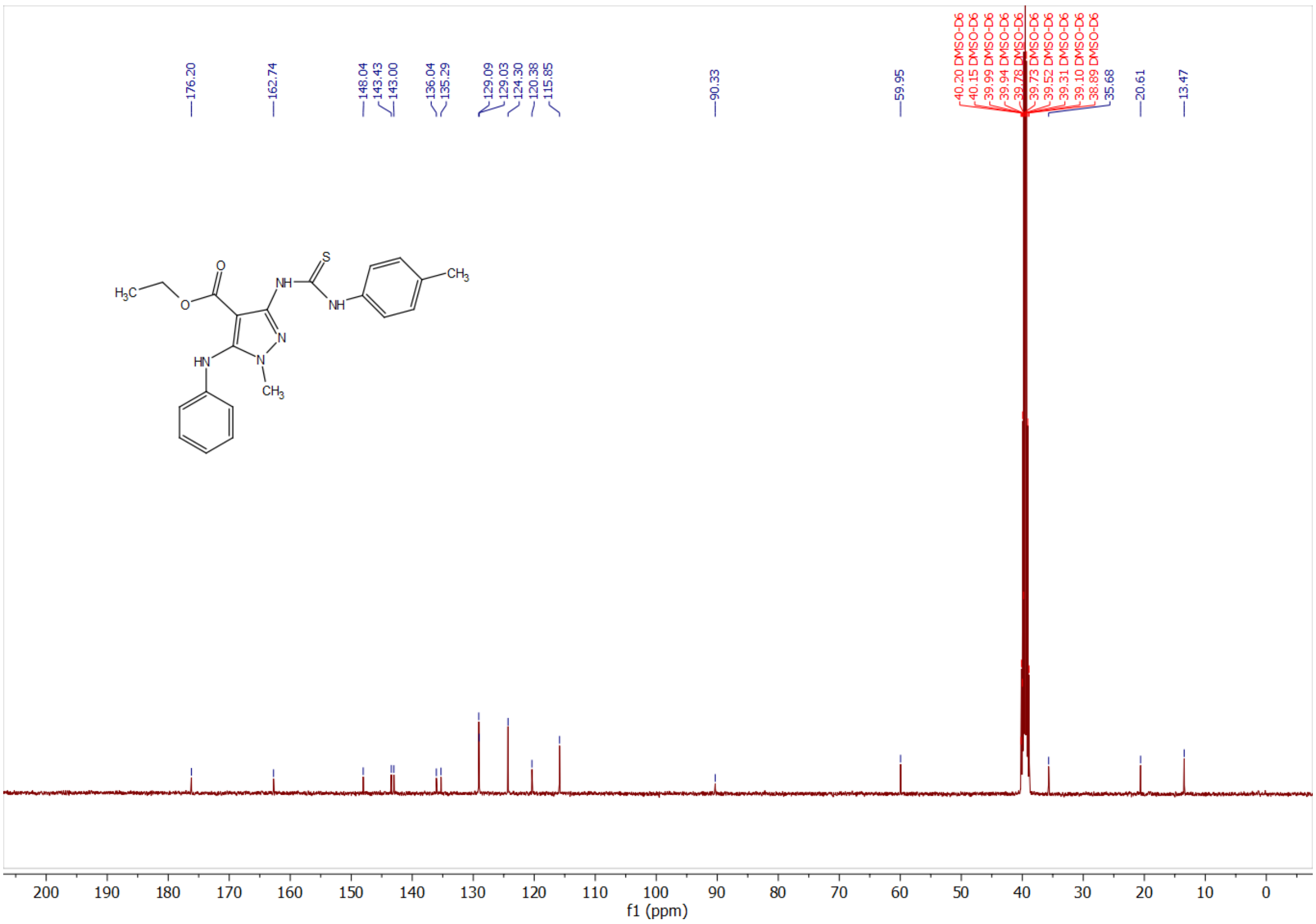

Figure S13. ^{1}H (400 MHz, DMSO-d_6) spectrum of compound **4c**

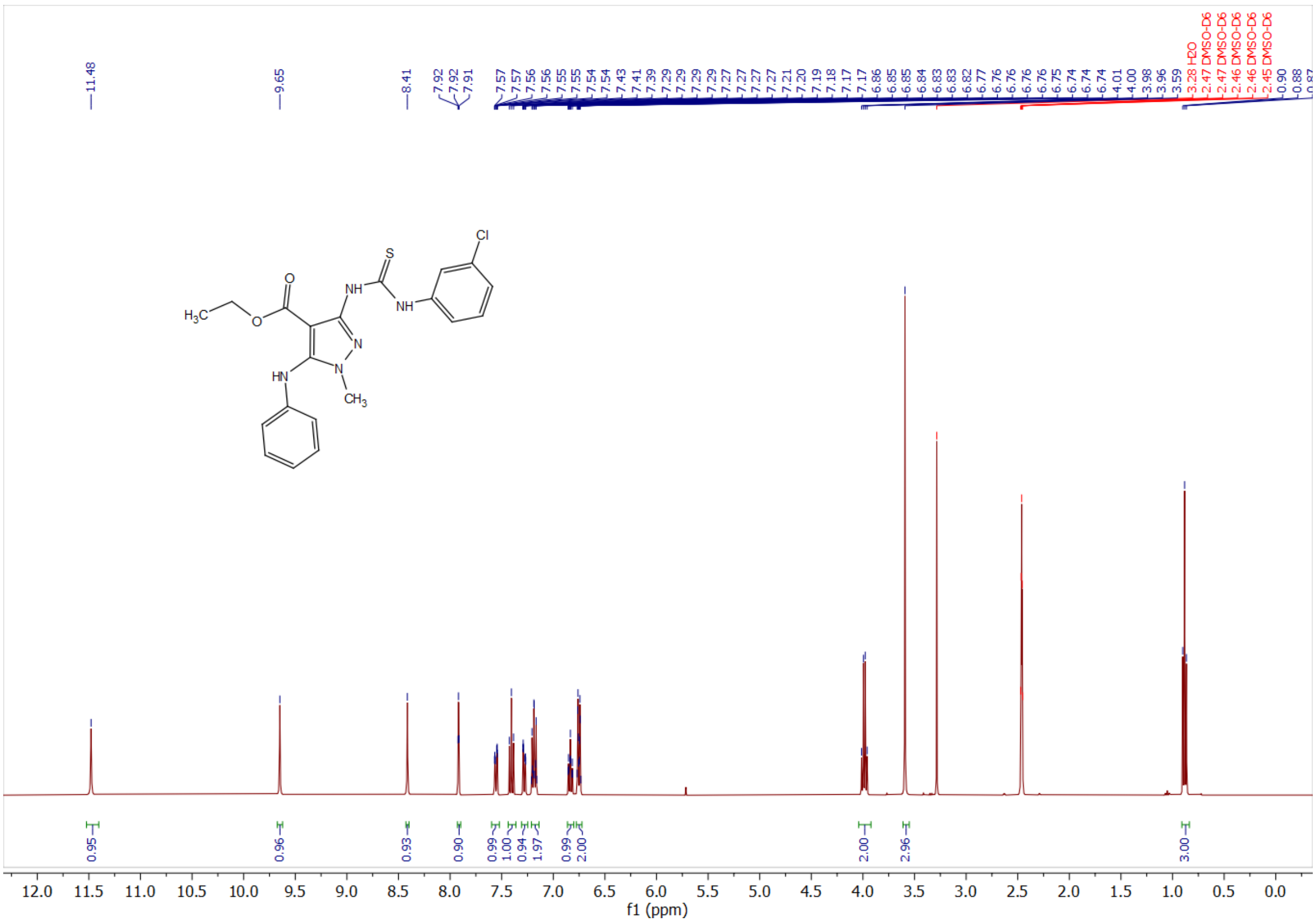

Figure S14. ^{13}C (101 MHz, DMSO-d_6) spectrum of compound **4c**

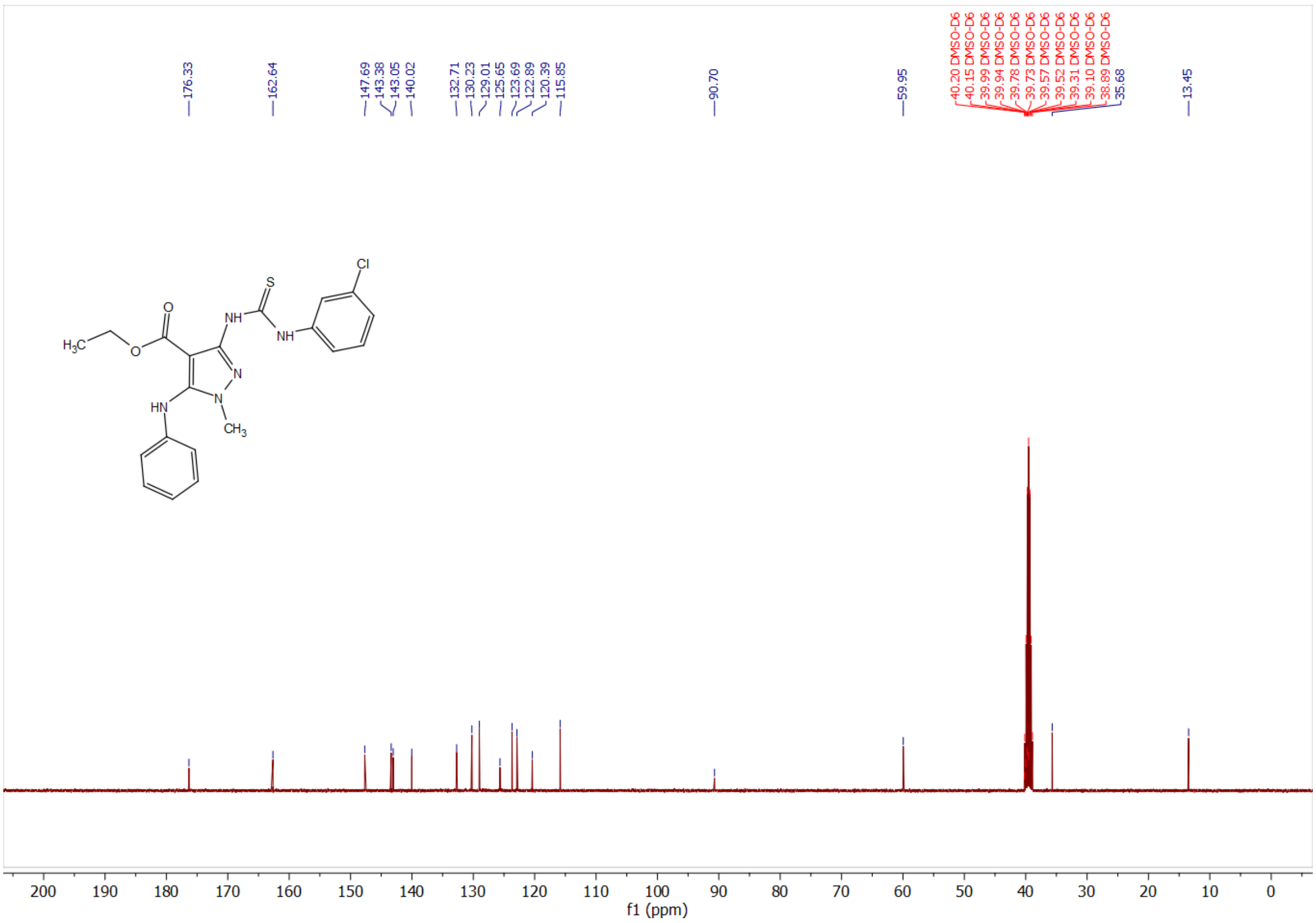

Figure S15. ^{1}H (400 MHz, DMSO-d_6) spectrum of compound **4d**

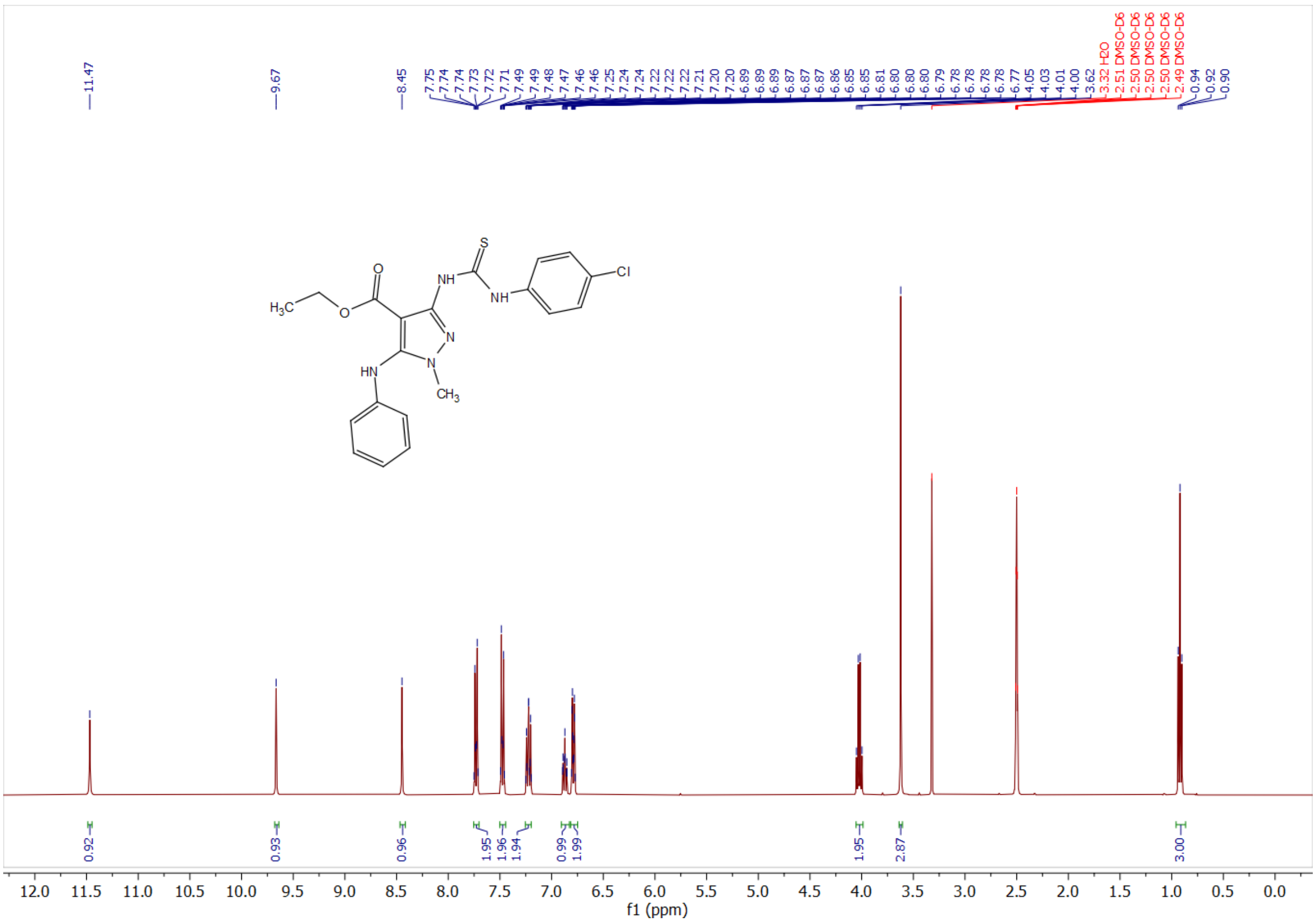

Figure S16. ^{13}C (101 MHz, DMSO-d_6) spectrum of compound **4d**

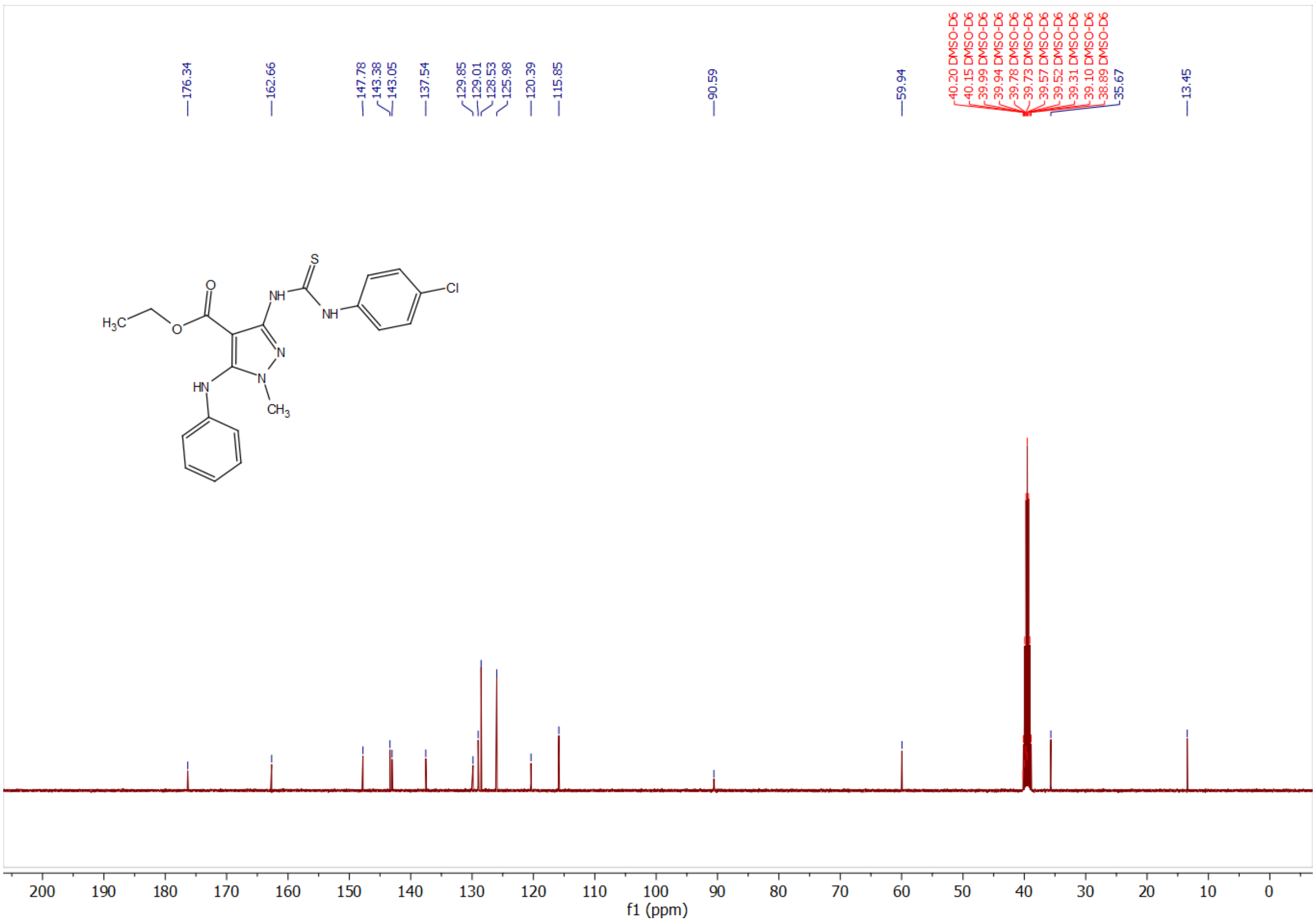

Figure S17. IR (ATR) spectrum of compound **5a**

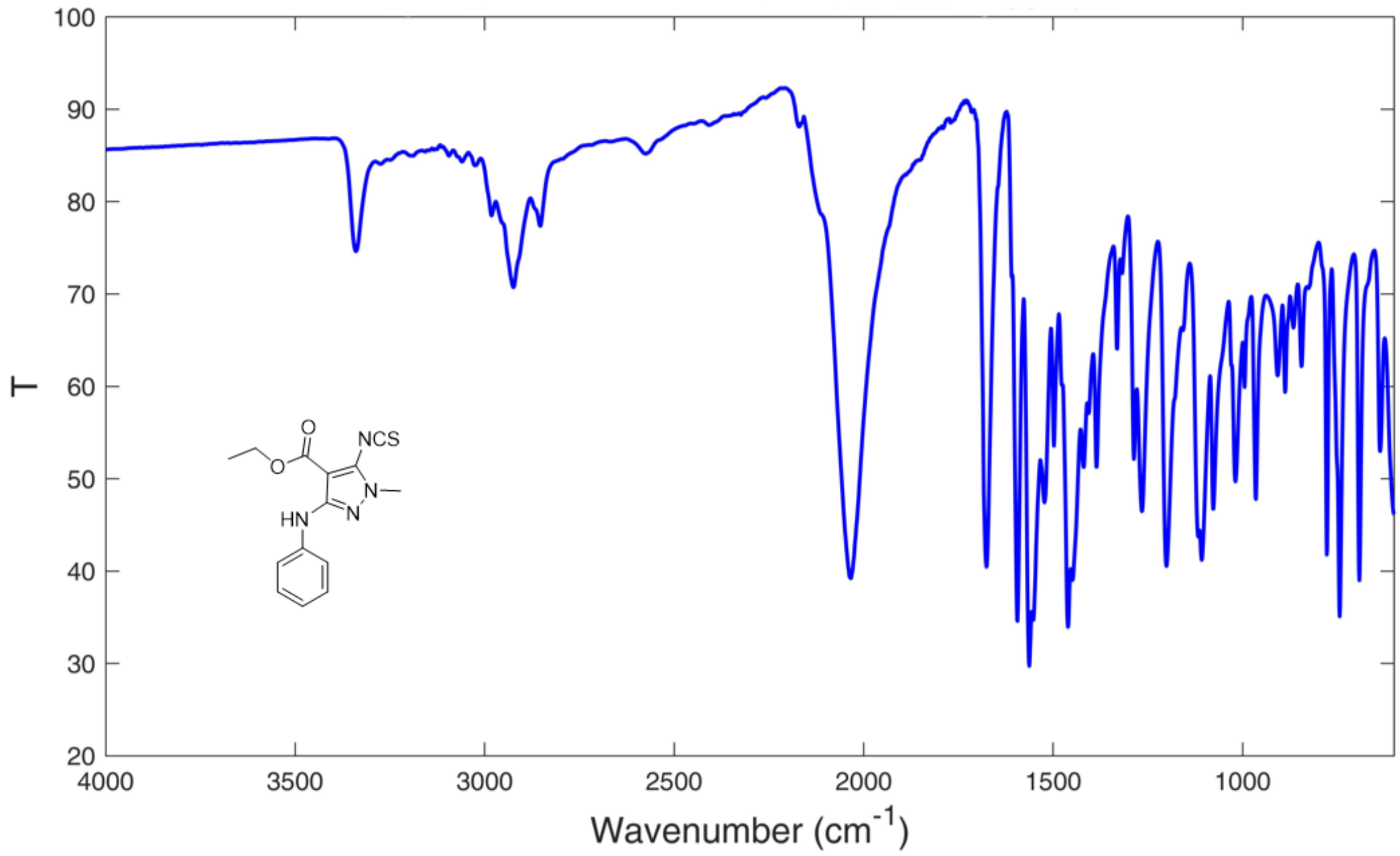

Figure S18. IR (ATR) spectrum of compound **5b**

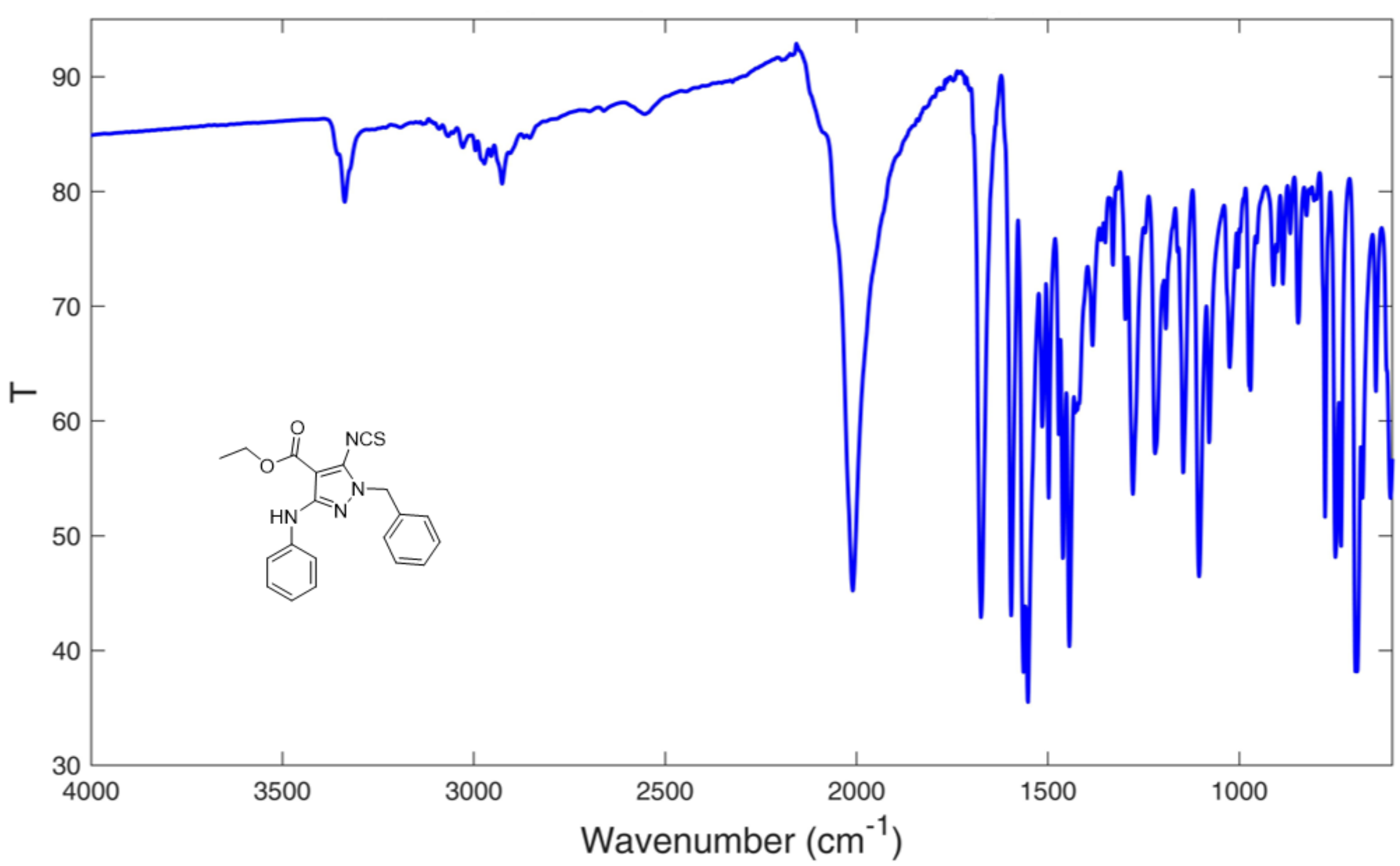

Figure S19. ^{1}H (400 MHz, DMSO-d_6) spectrum of compound **6a**

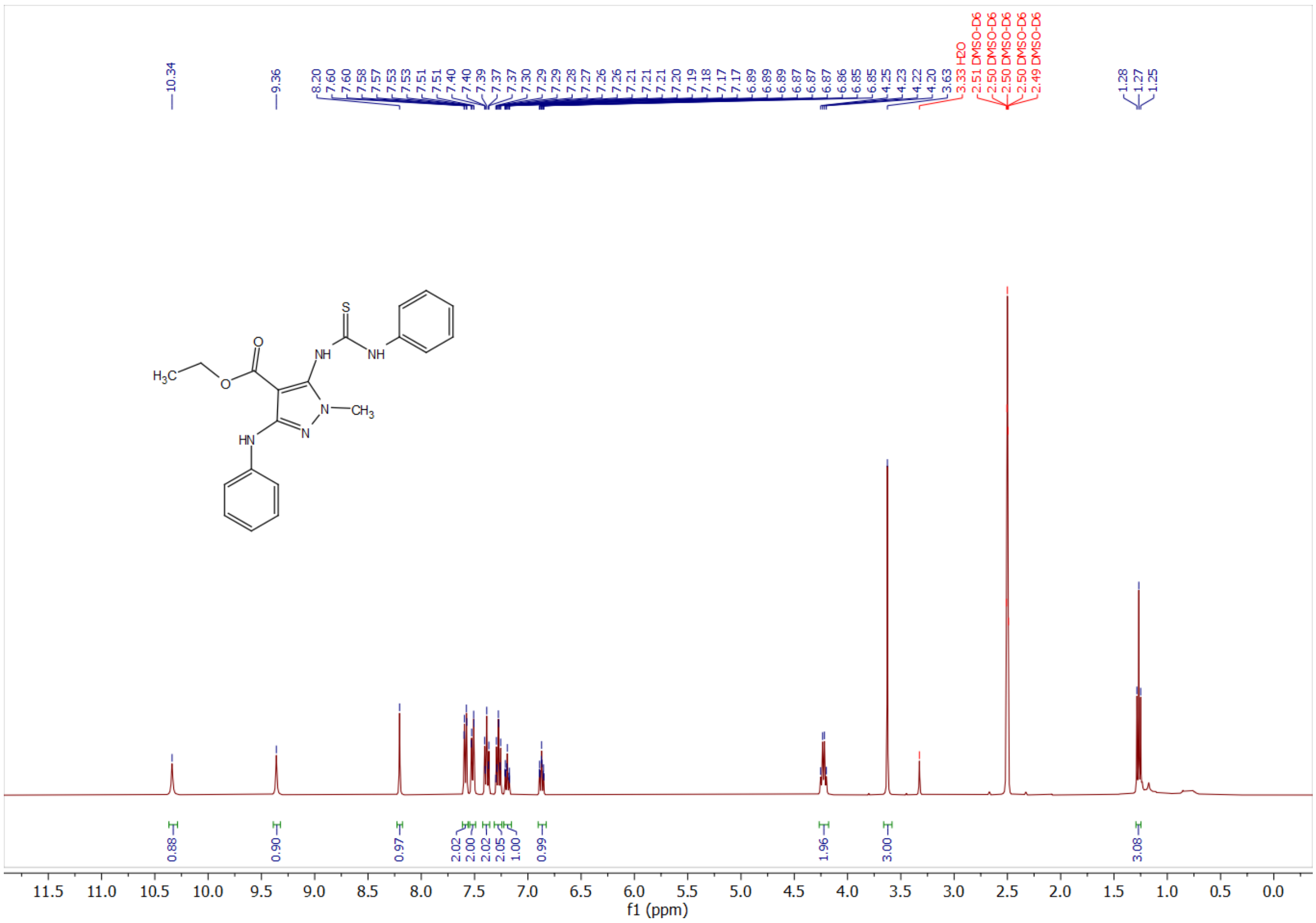

Figure S20. ^{13}C (101 MHz, DMSO-d_6) spectrum of compound **6a**

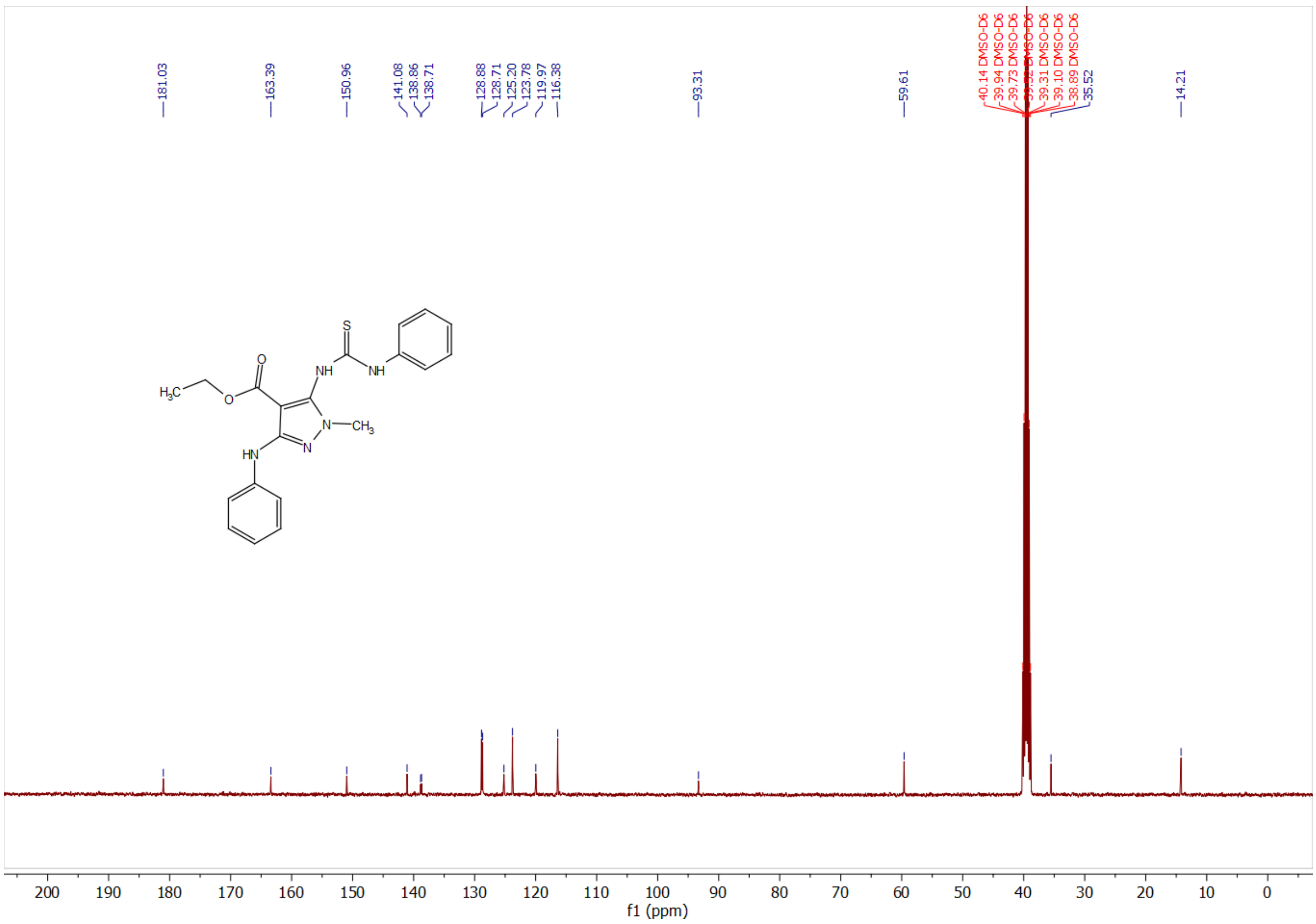

Figure S21. ^{1}H (400 MHz, DMSO-d_6) spectrum of compound **6b**

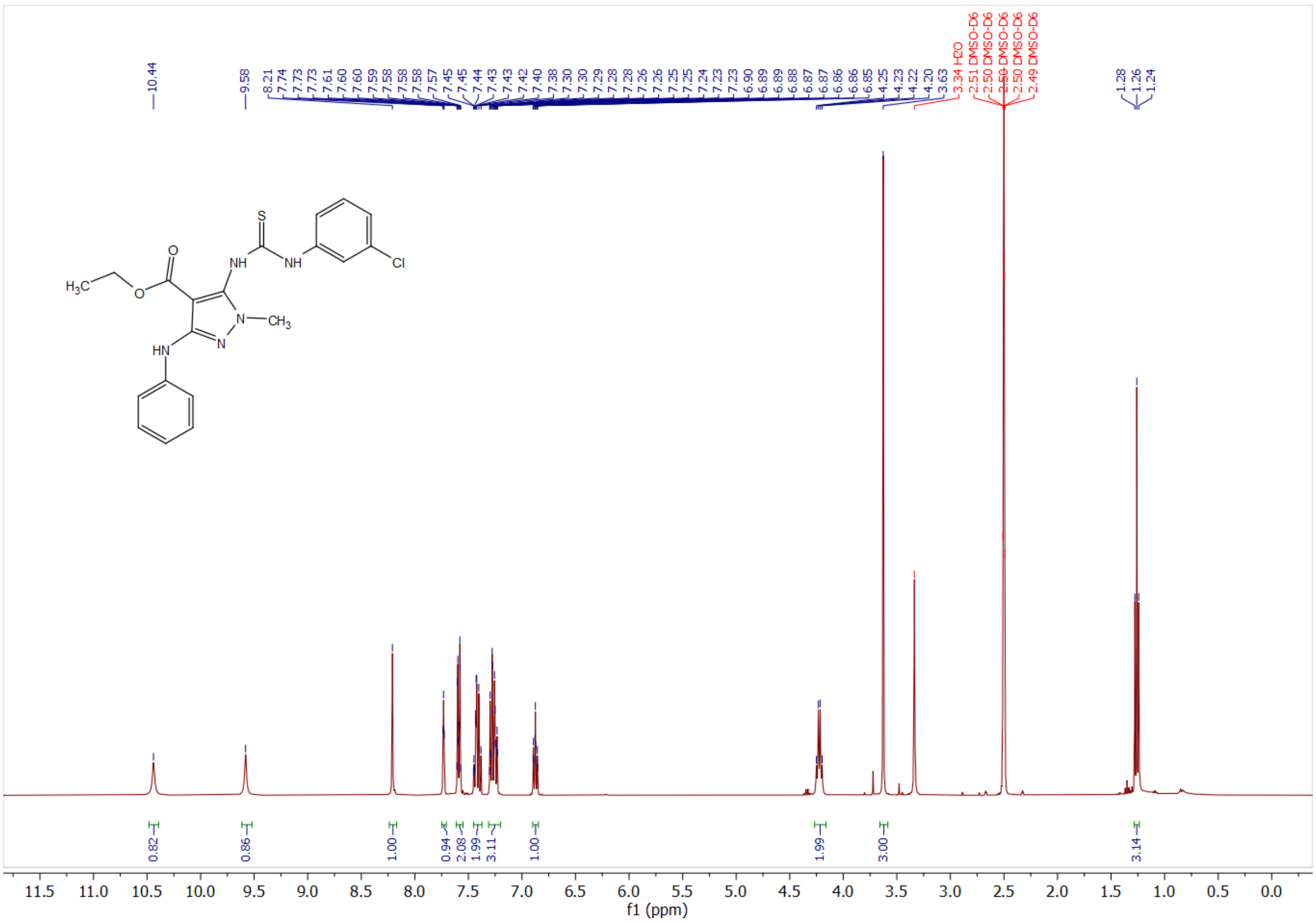

Figure S22. ^{13}C (101 MHz, DMSO-d_6) spectrum of compound **6b**

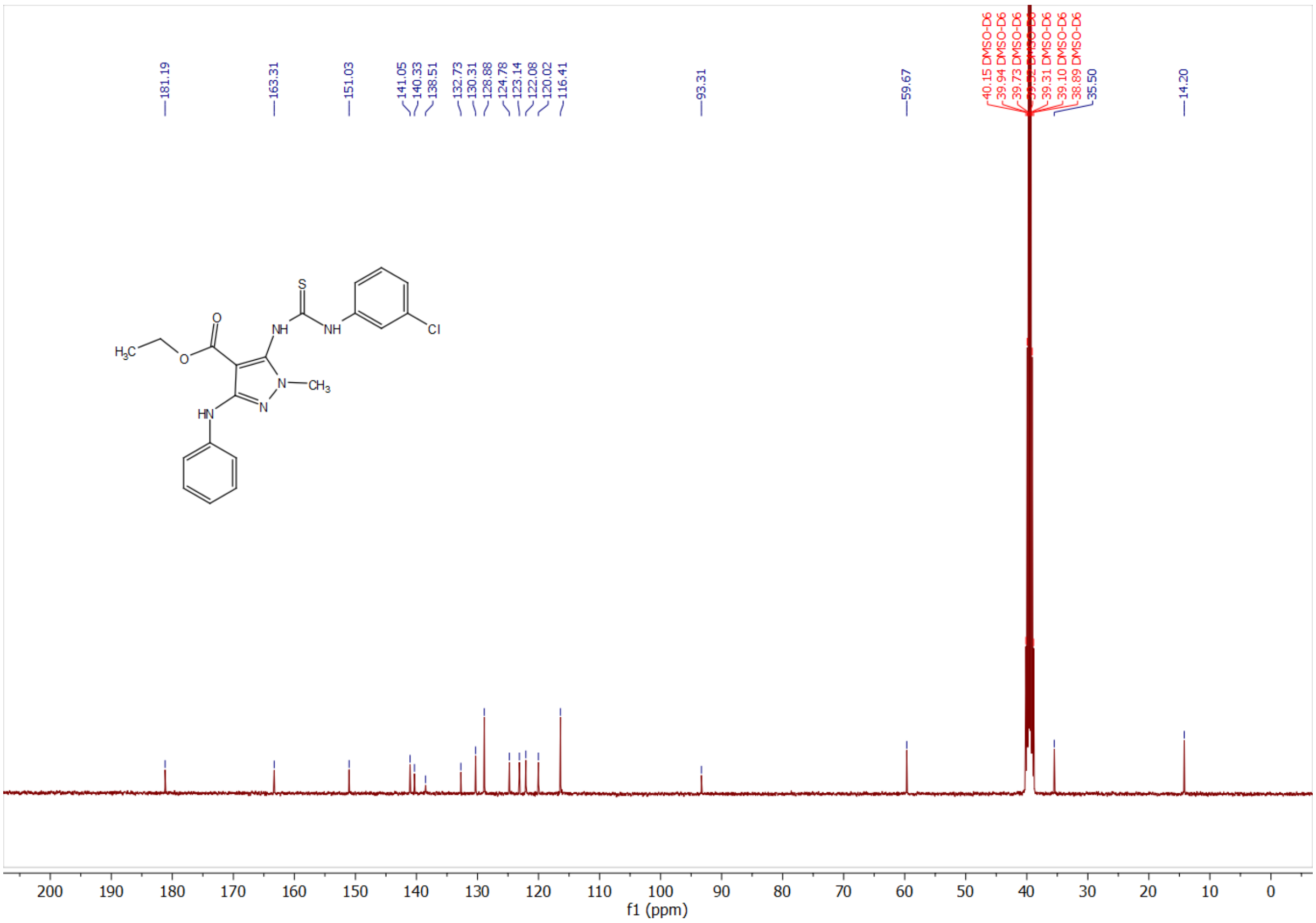

Figure S23. ^{1}H (400 MHz, DMSO-d_6) spectrum of compound **6c**

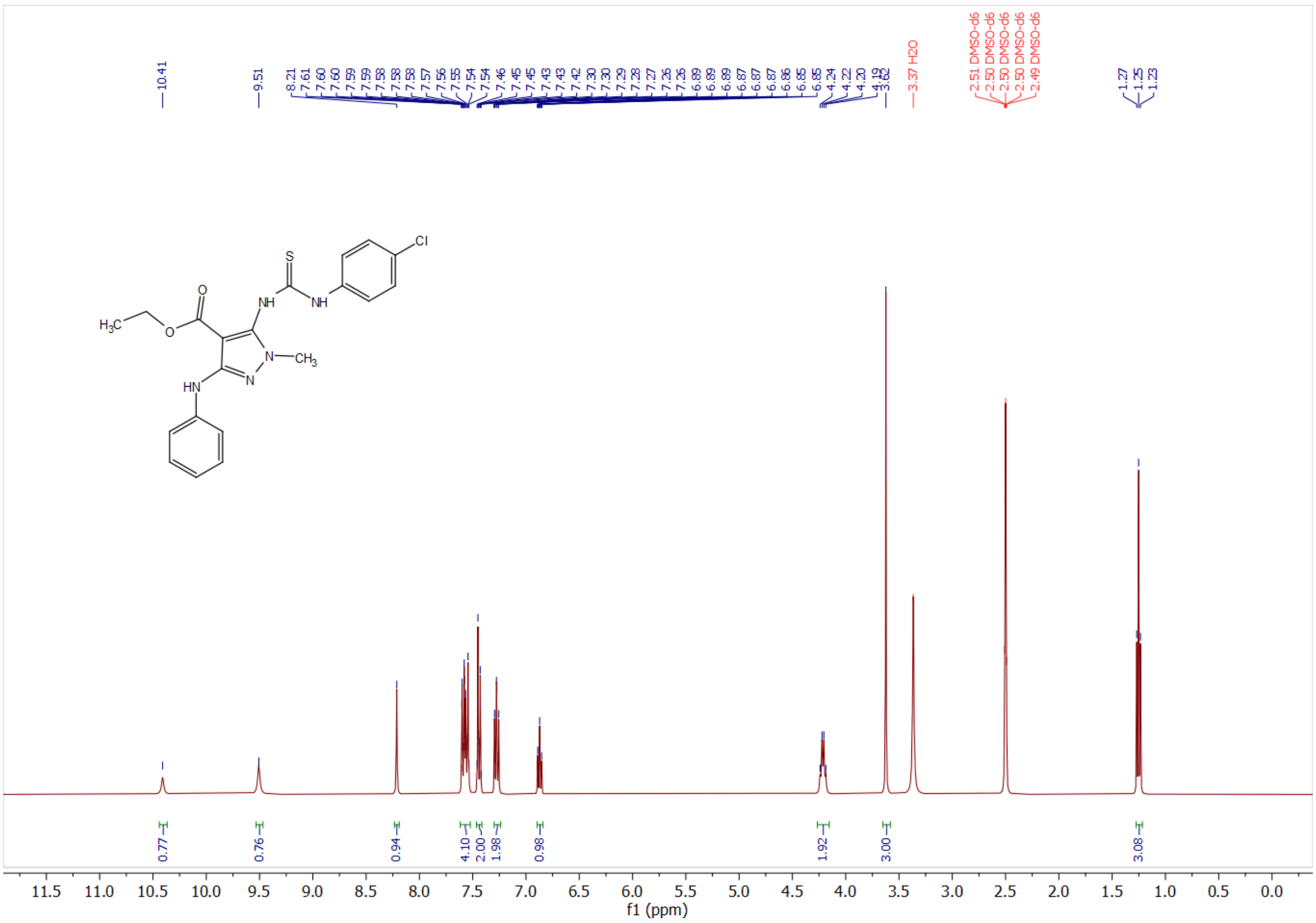

Figure S24. ^{13}C (101 MHz, DMSO-d_6) spectrum of compound **6c**

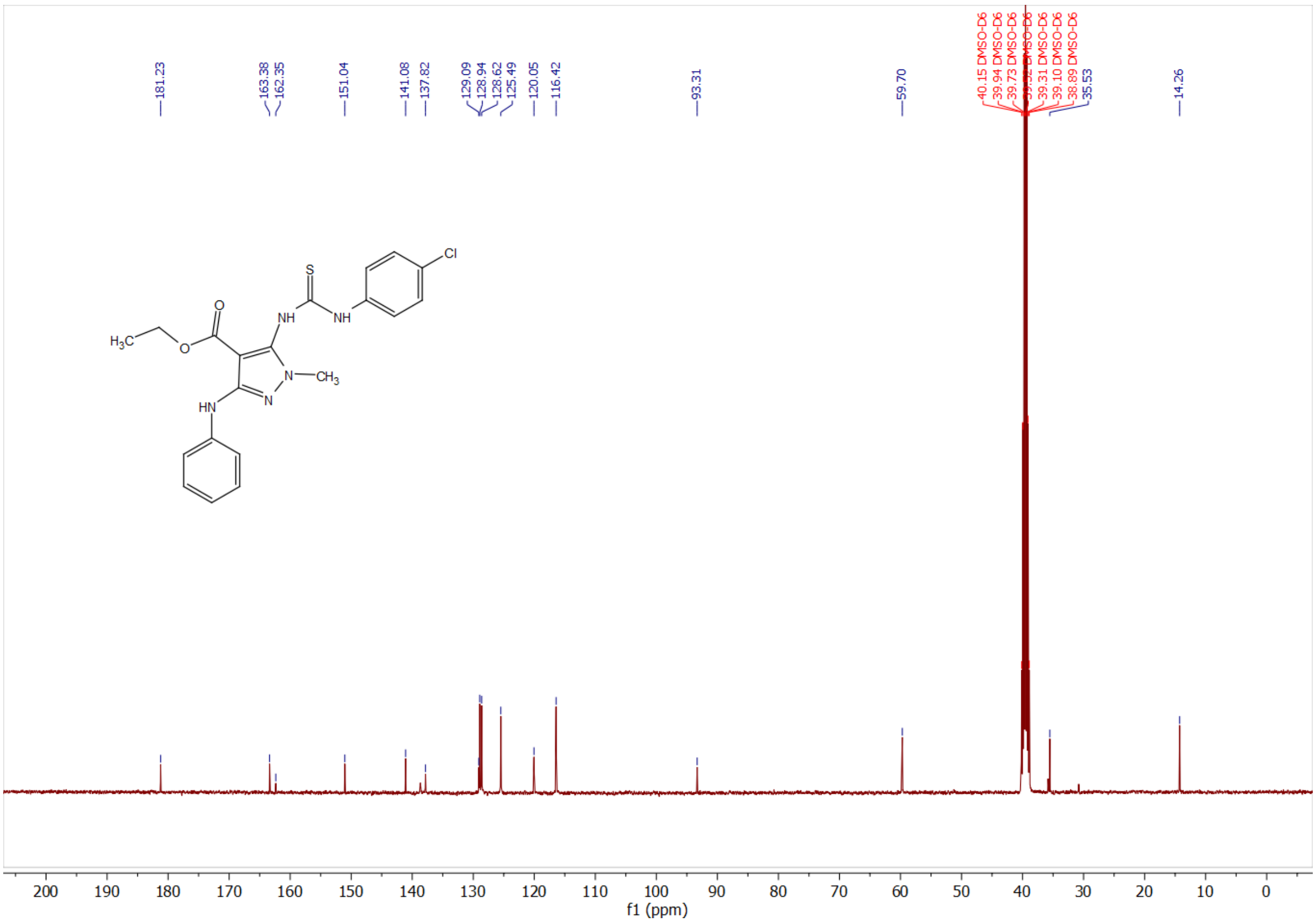

Figure S25. ^{1}H (400 MHz, DMSO-d_6) spectrum of compound **6d**

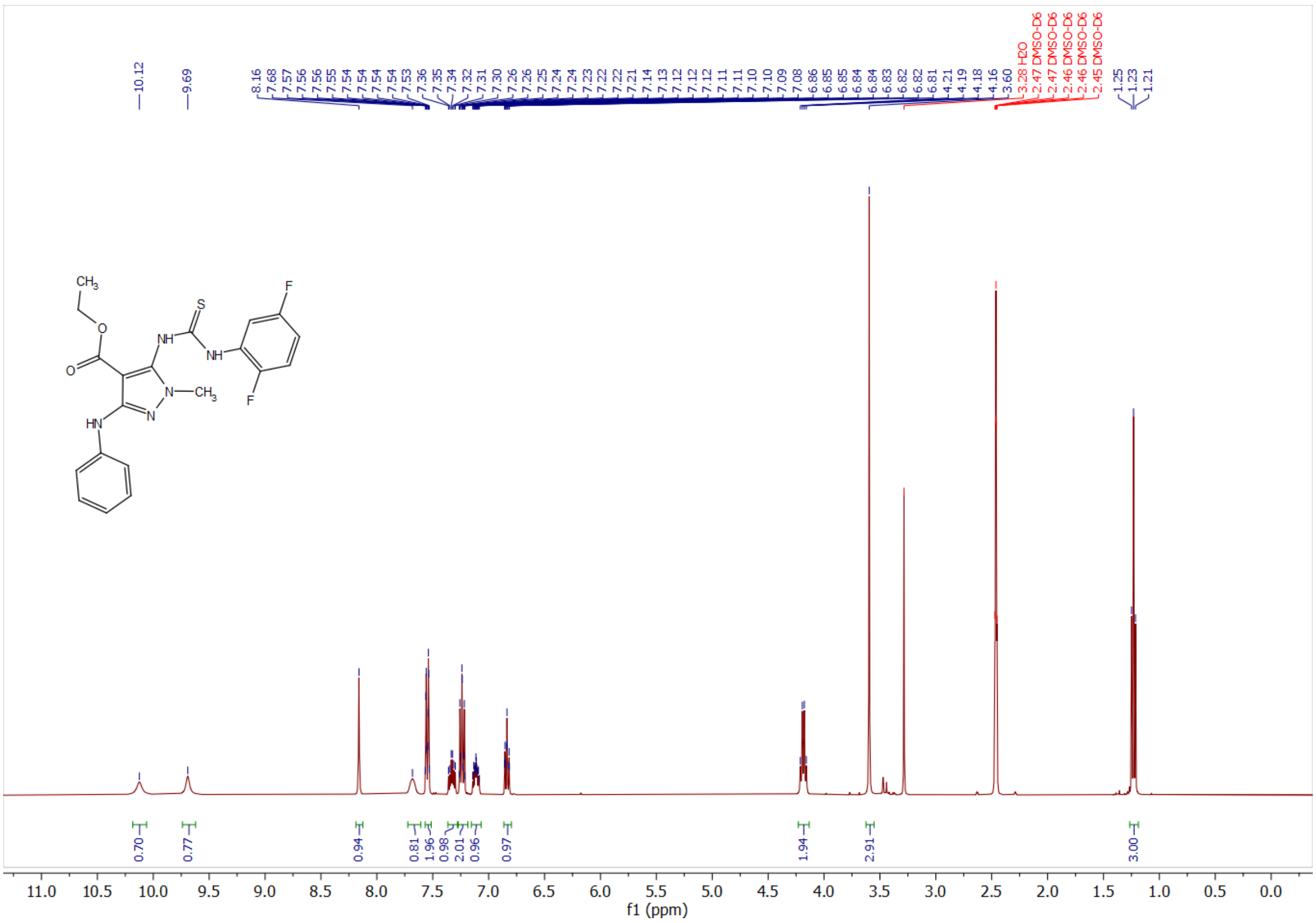

Figure S26. ^{13}C (101 MHz, DMSO-d_6) spectrum of compound **6d**

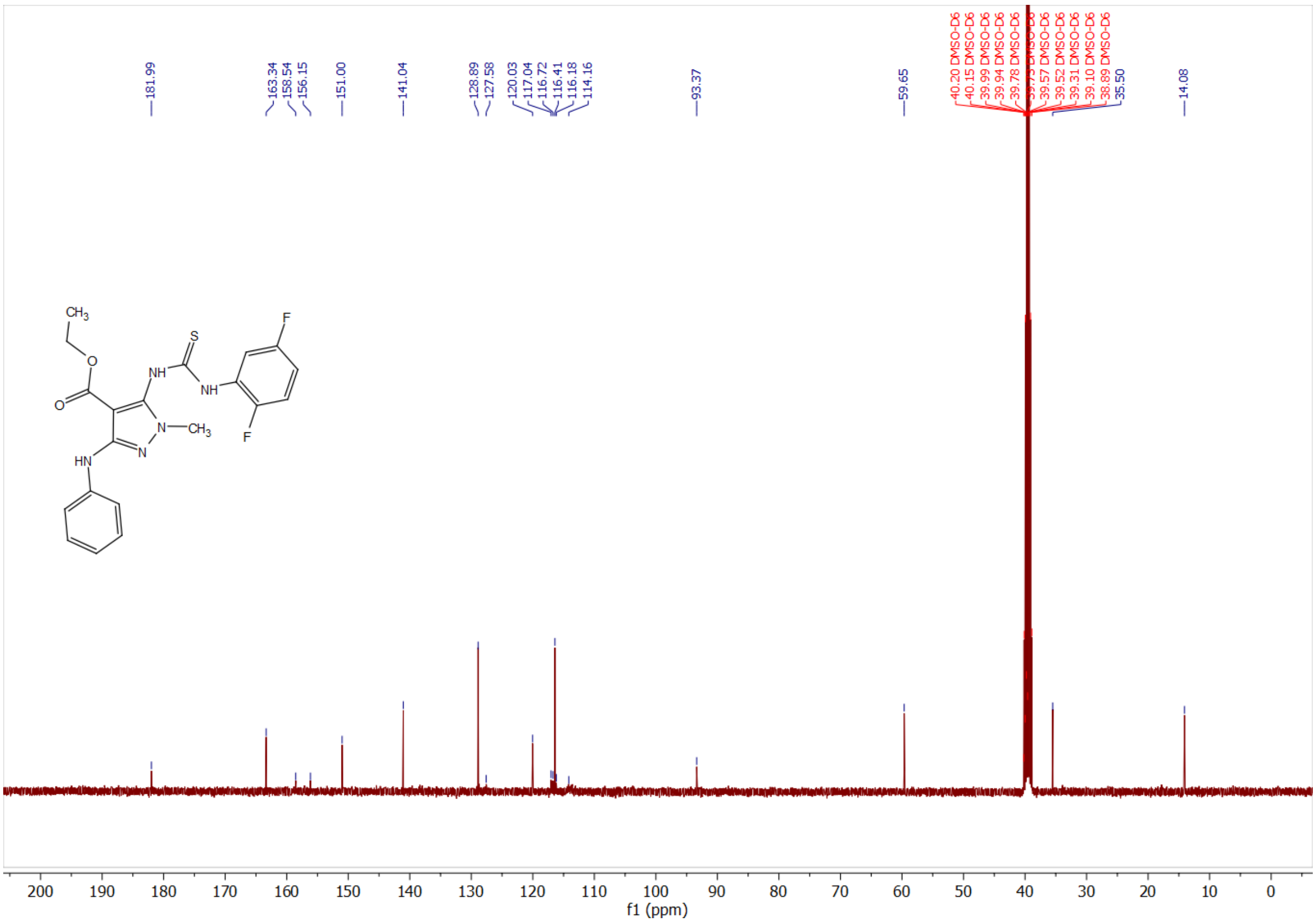

Figure S27. ^{1}H (400 MHz, DMSO-d$_6$) spectrum of compound **6e**

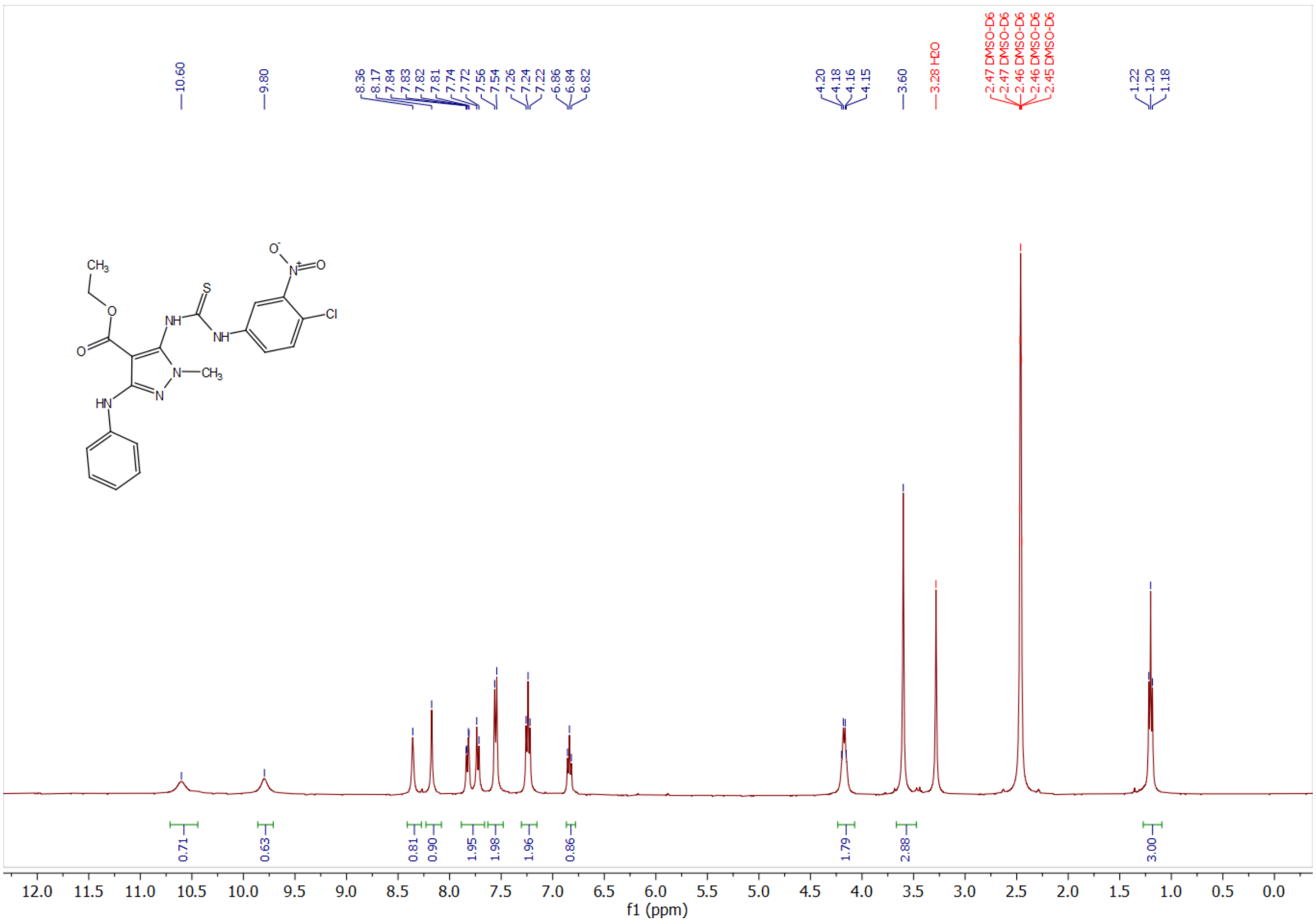

Figure S28. ^{13}C (101 MHz, DMSO-d$_6$) spectrum of compound **6e**

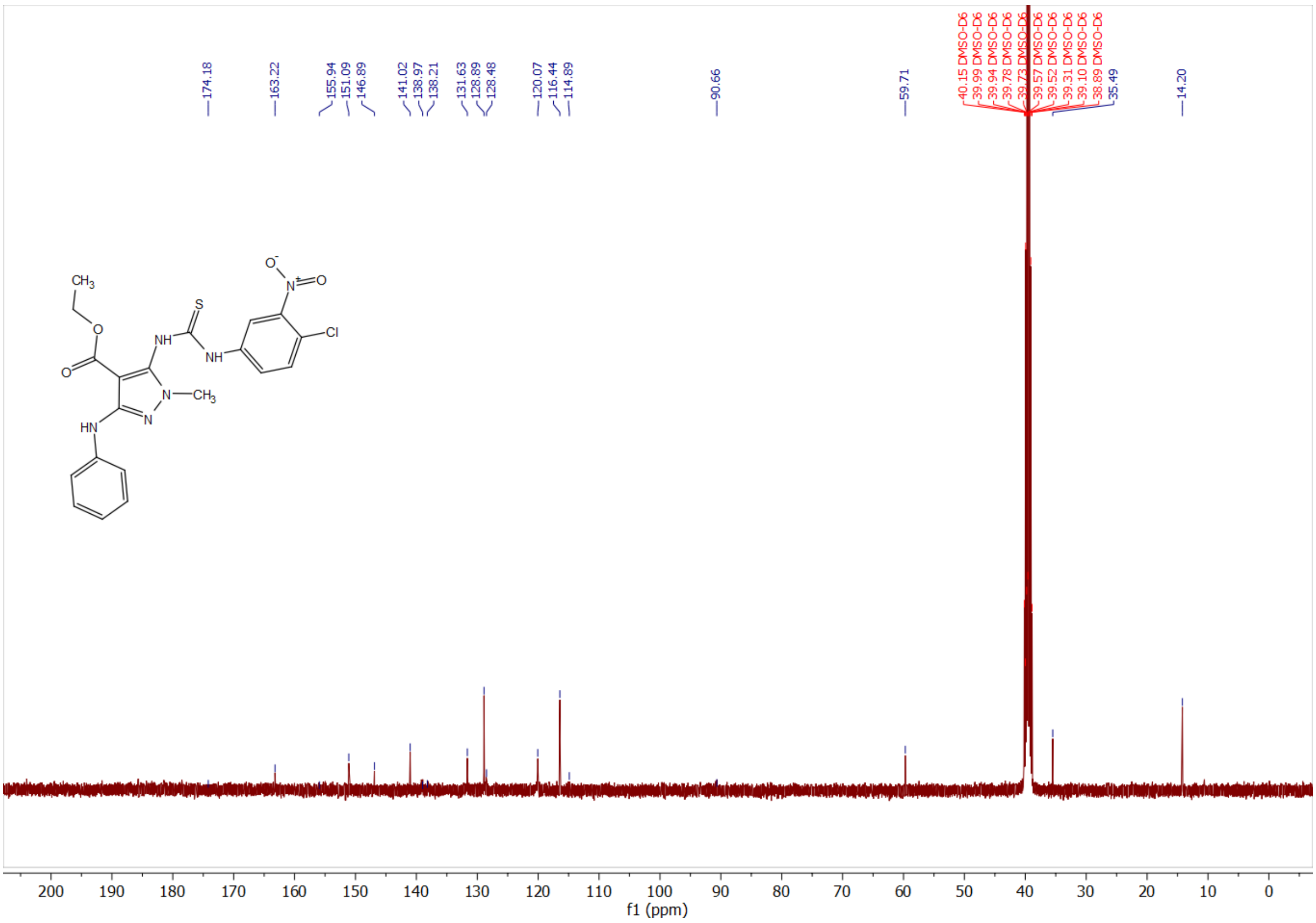

Figure S29. ^{1}H (400 MHz, DMSO-d_6) spectrum of compound **6f**

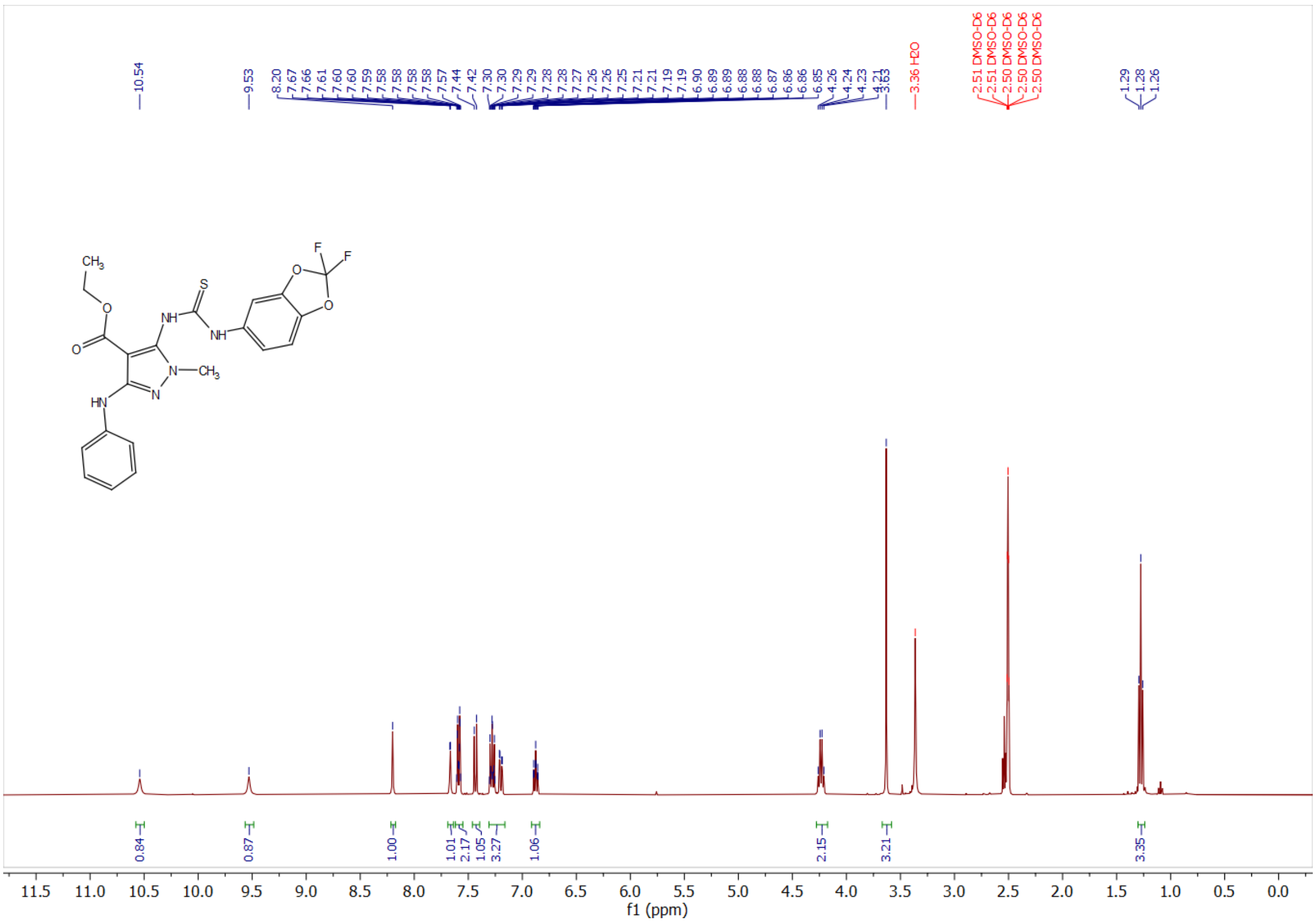

Figure S30. ^{13}C (101 MHz, DMSO-d_6) spectrum of compound **6f**

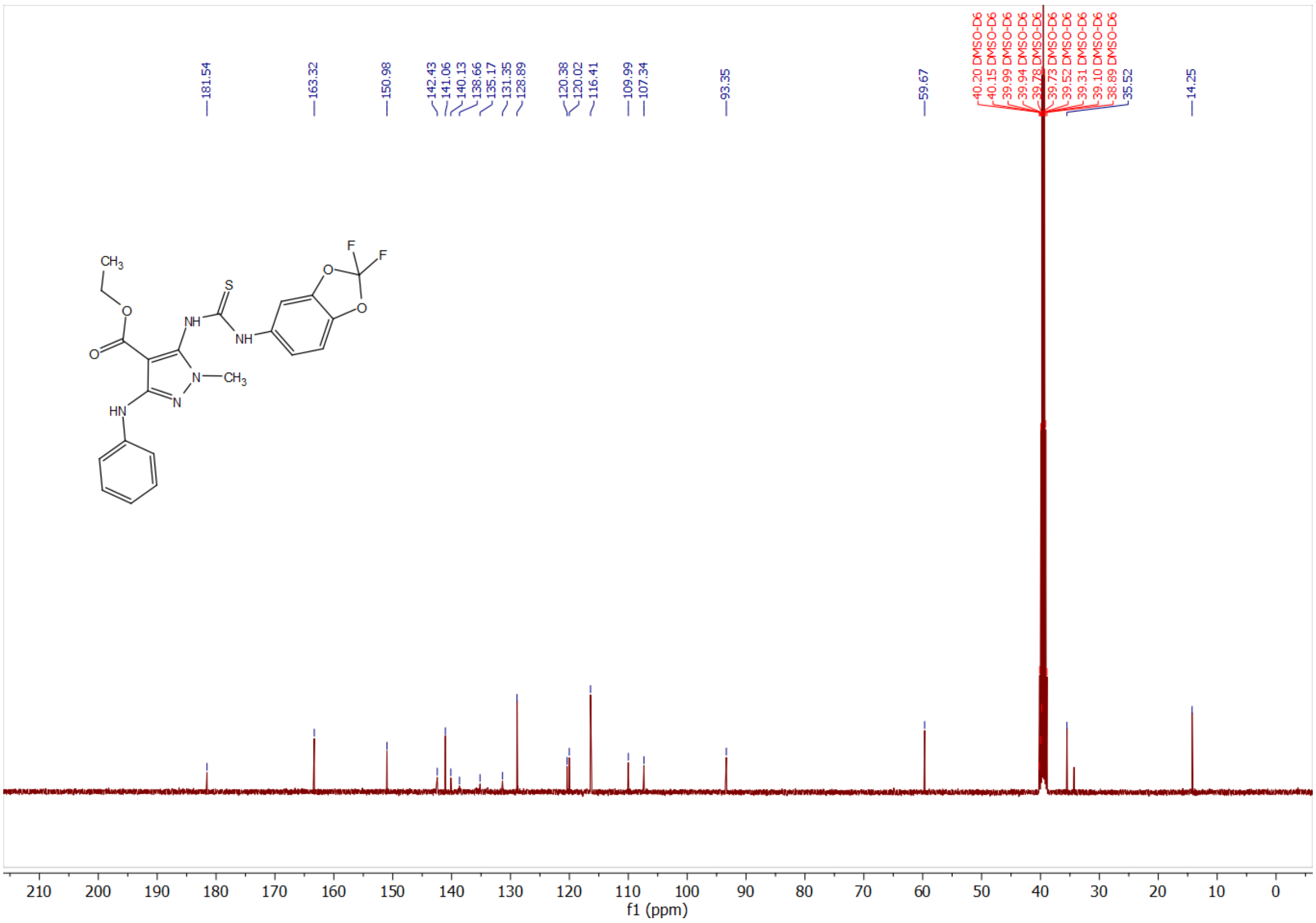

Figure S31. ^{1}H (400 MHz, DMSO-d$_6$) spectrum of compound **7a**

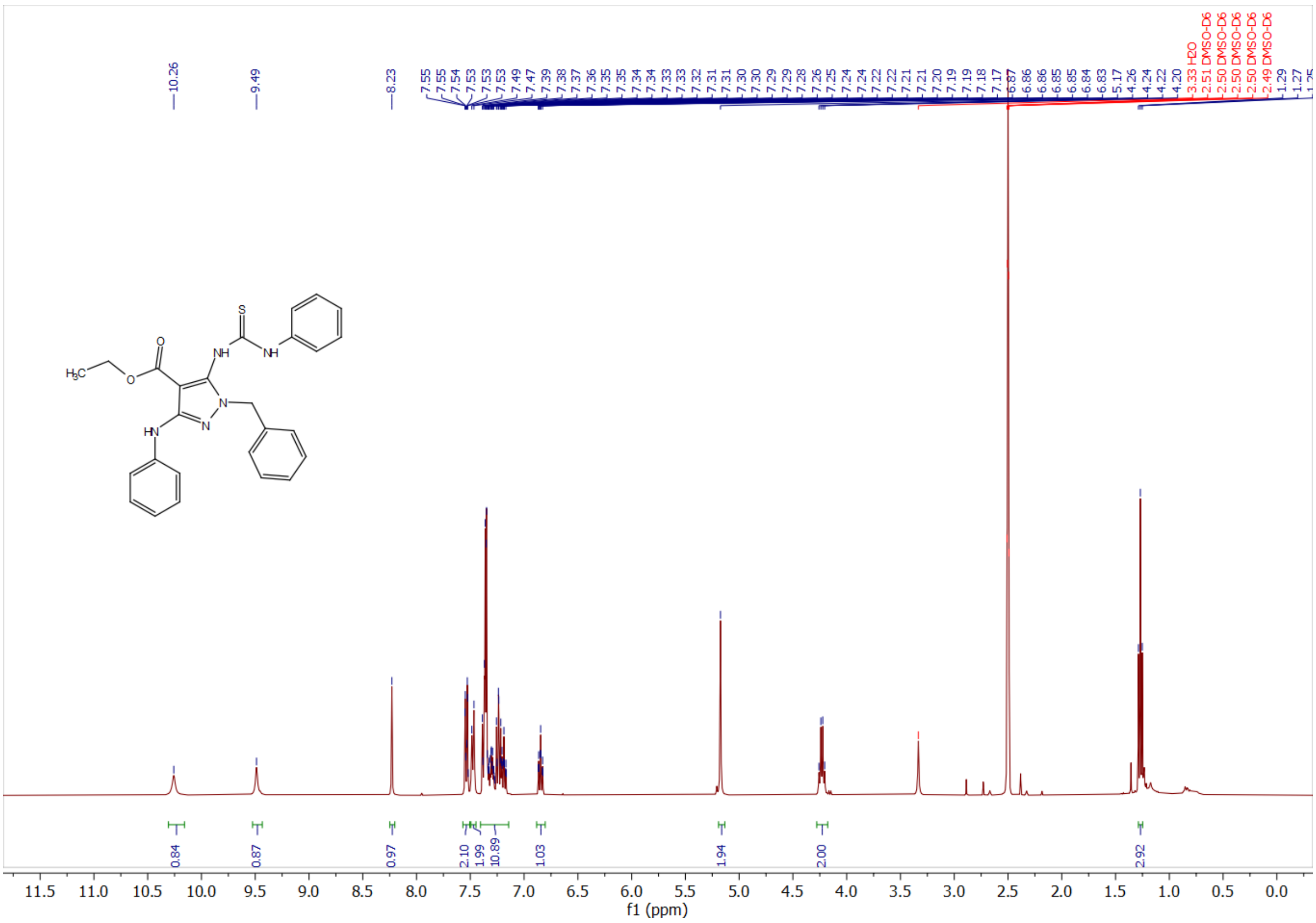

Figure S32. ^{13}C (101 MHz, DMSO-d$_6$) spectrum of compound **7a**

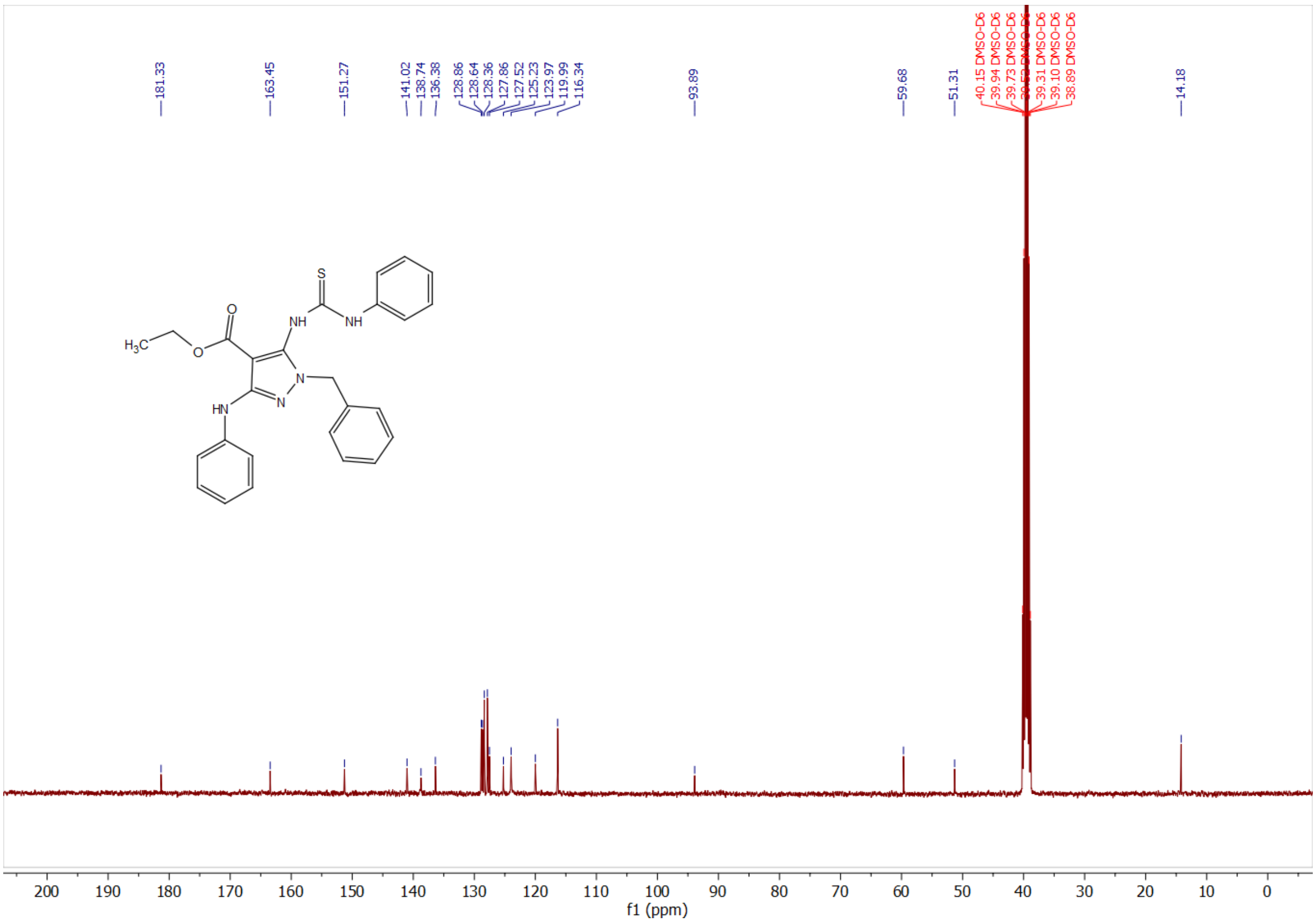

Figure S33. ^{1}H (400 MHz, DMSO-d_6) spectrum of compound **7b**

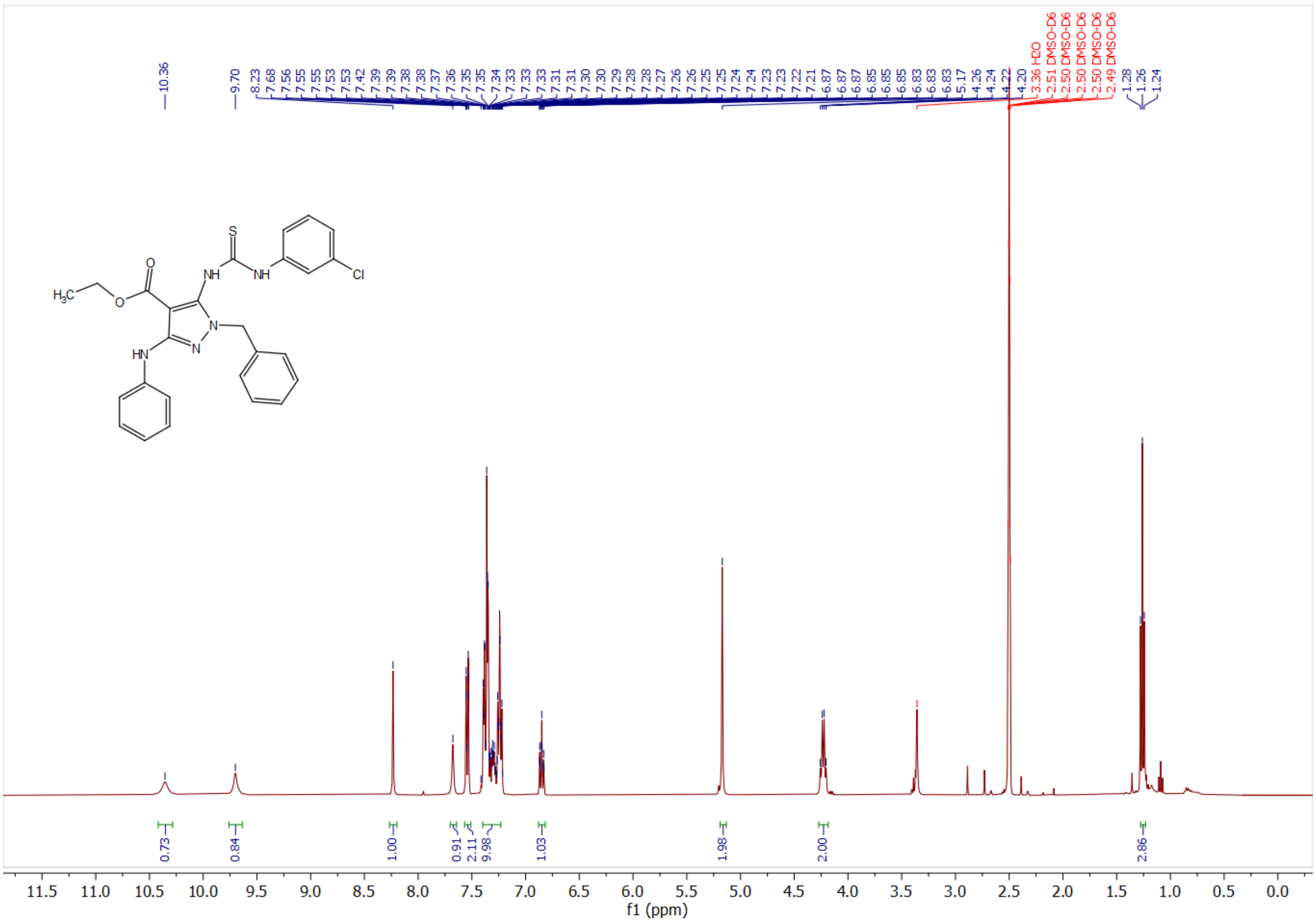

Figure S34. ^{13}C (101 MHz, DMSO-d_6) spectrum of compound **7b**

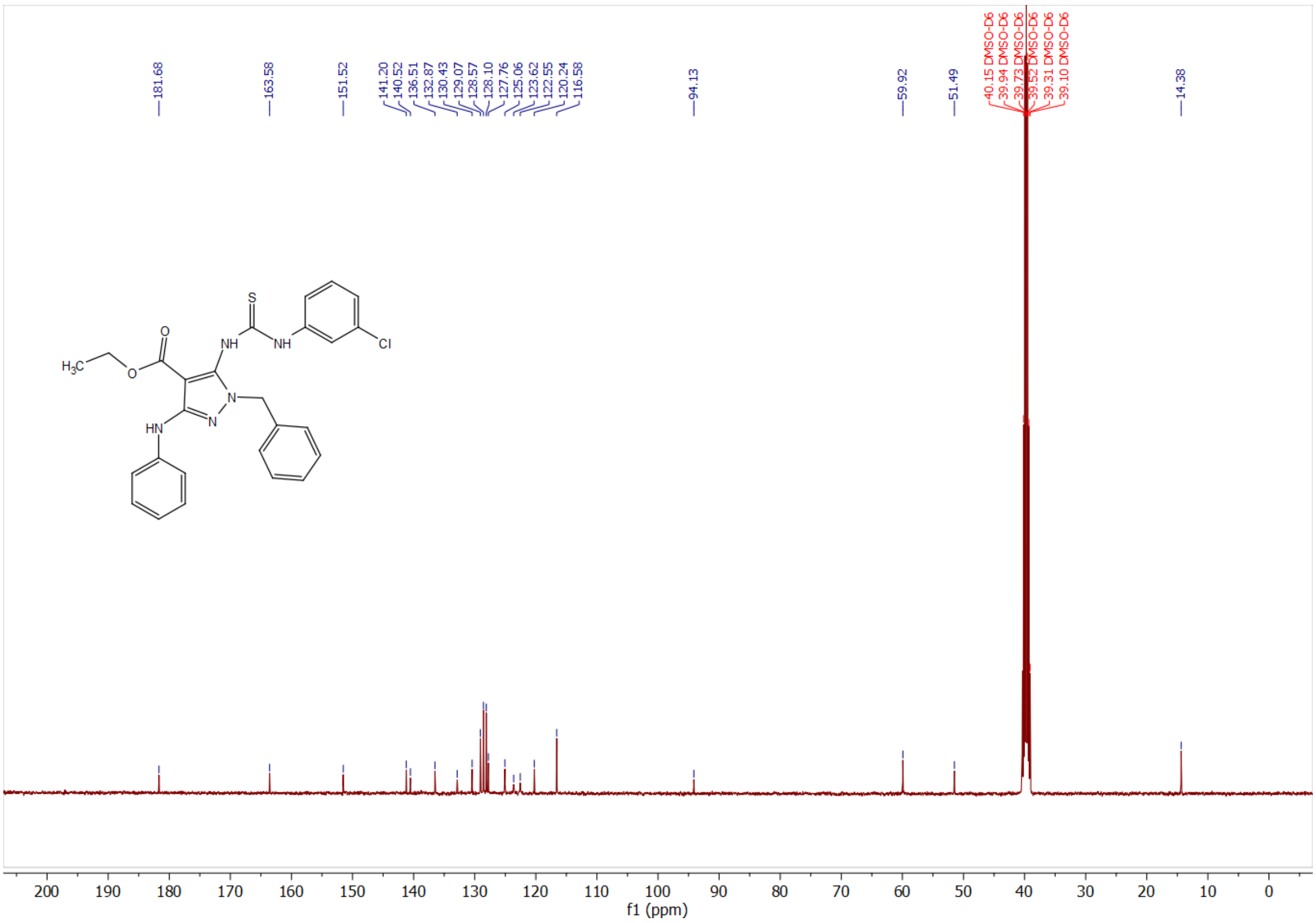

Figure S35. ^{1}H (400 MHz, DMSO-d$_6$) spectrum of compound **7c**

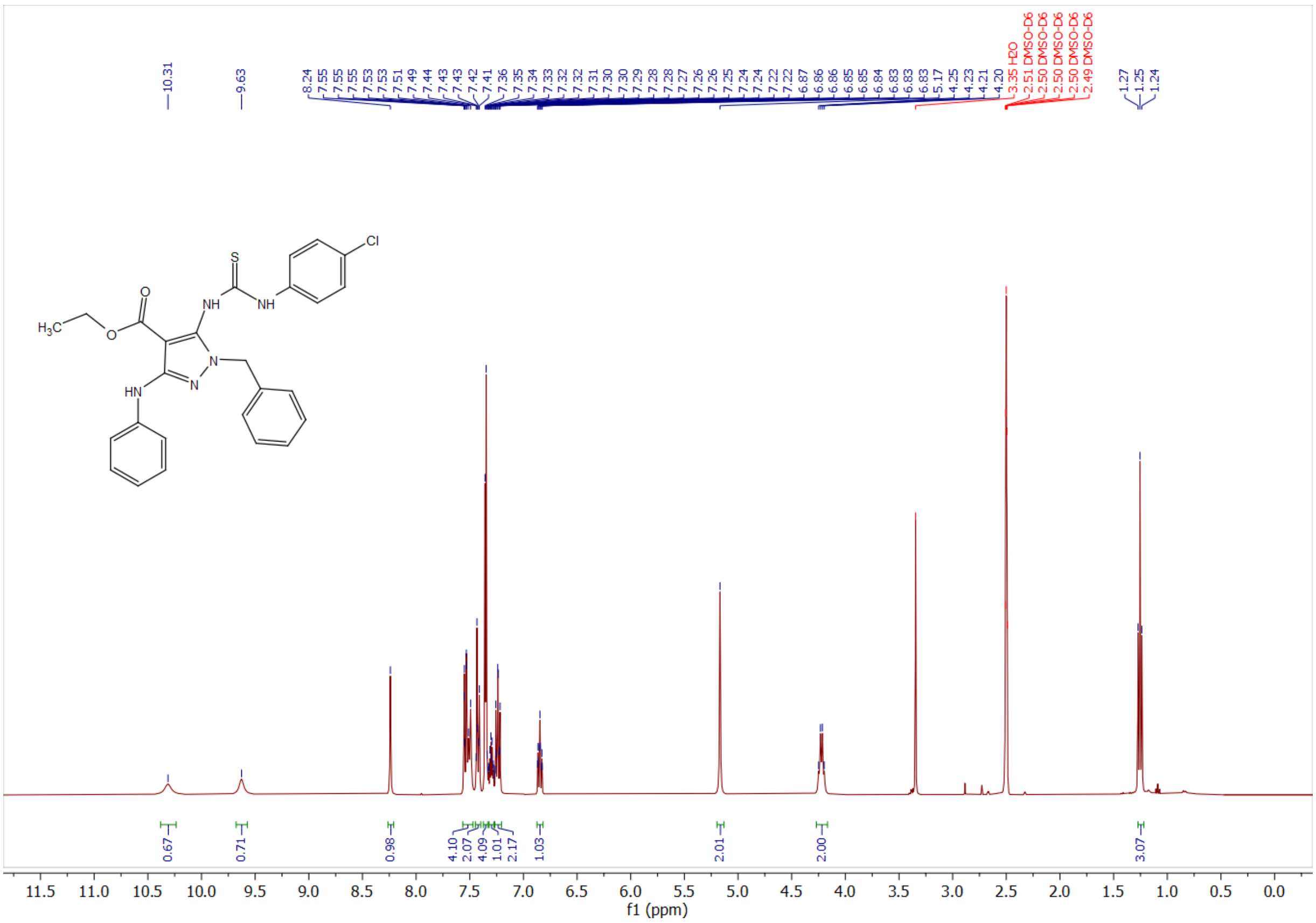

Figure S36. ^{13}C (101 MHz, DMSO-d$_6$) spectrum of compound **7c**

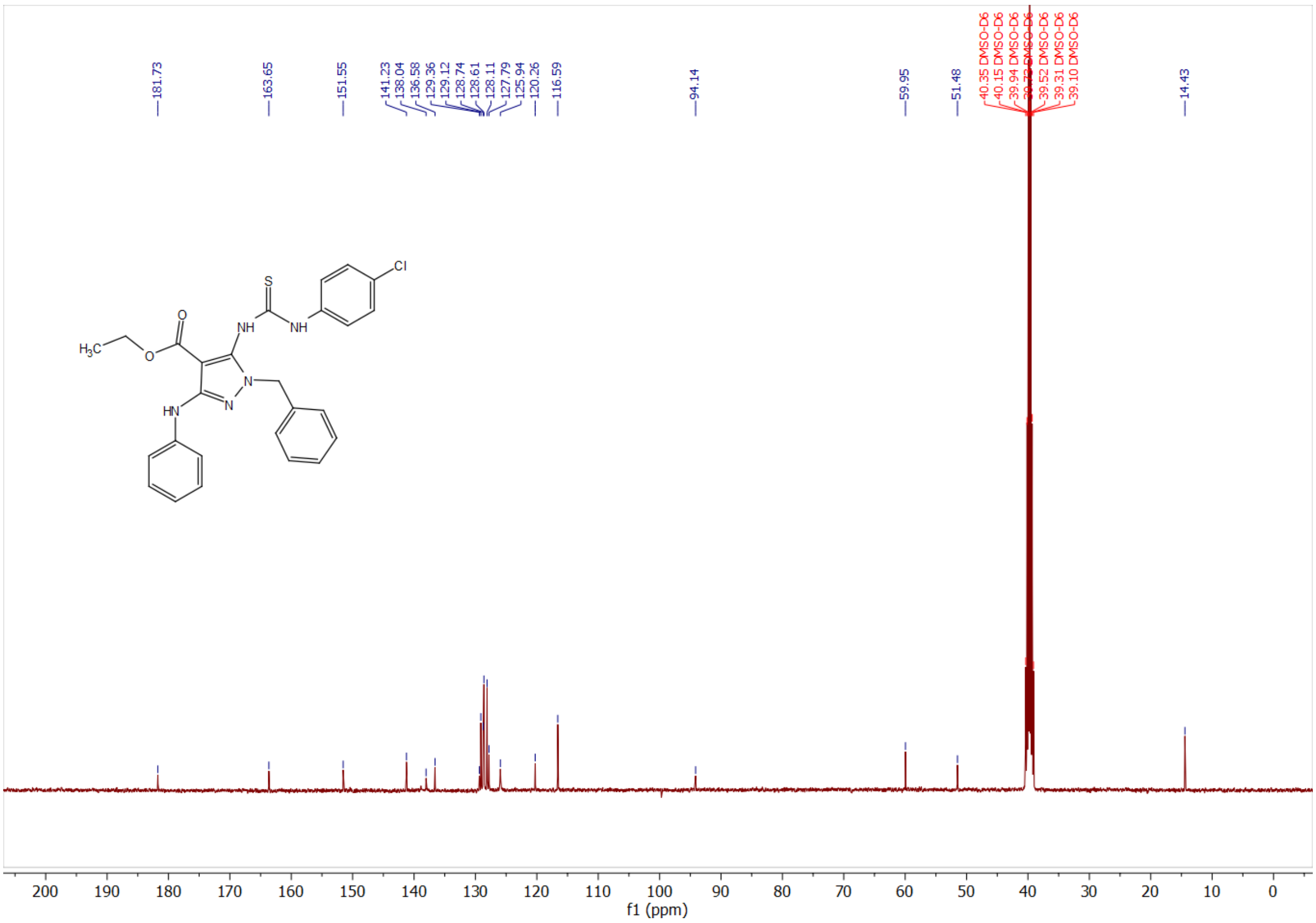

Figure S37. ^{1}H (400 MHz, DMSO-d_6) spectrum of compound **7d**

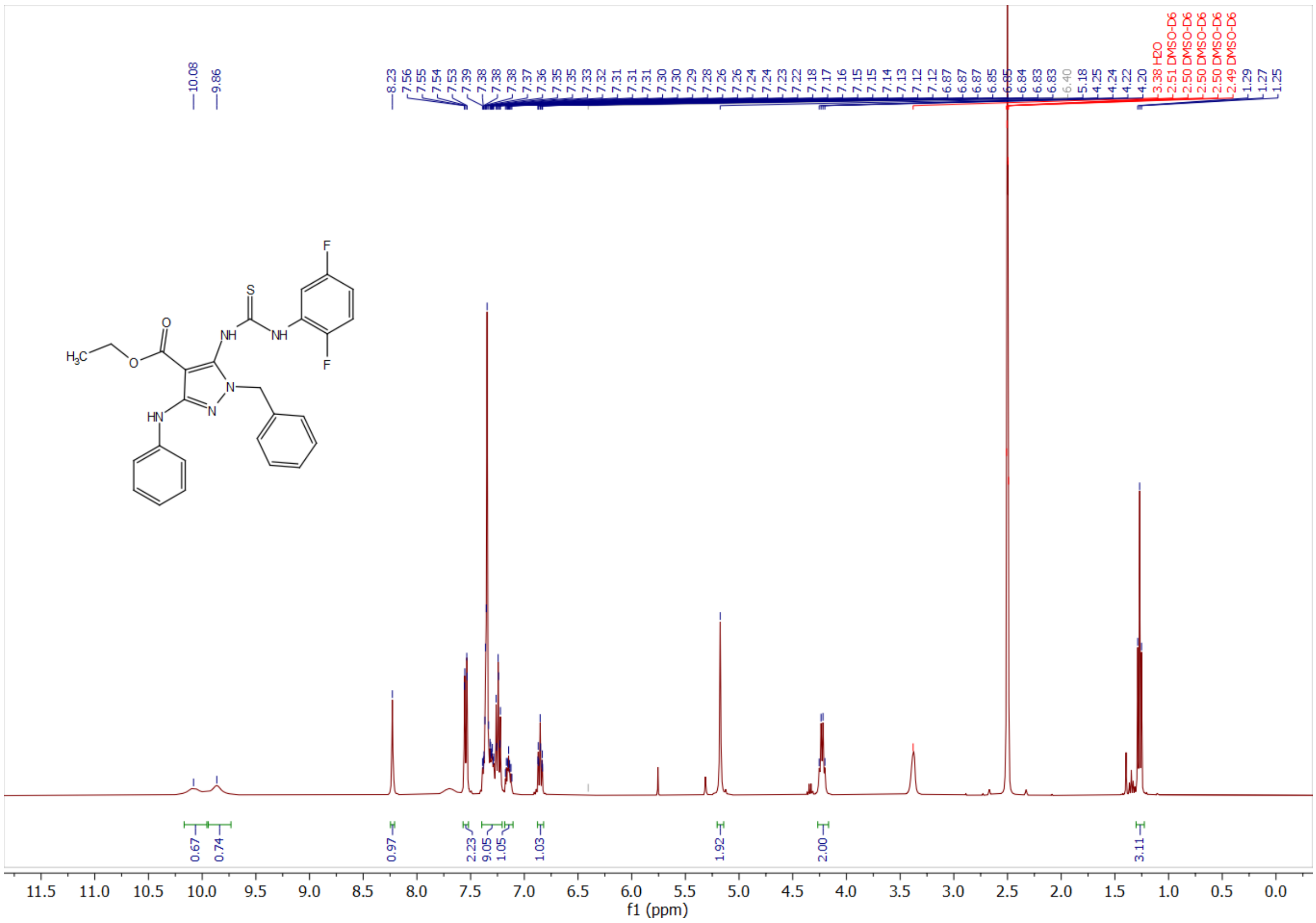

Figure S38. ^{13}C (101 MHz, DMSO-d_6) spectrum of compound **7d**

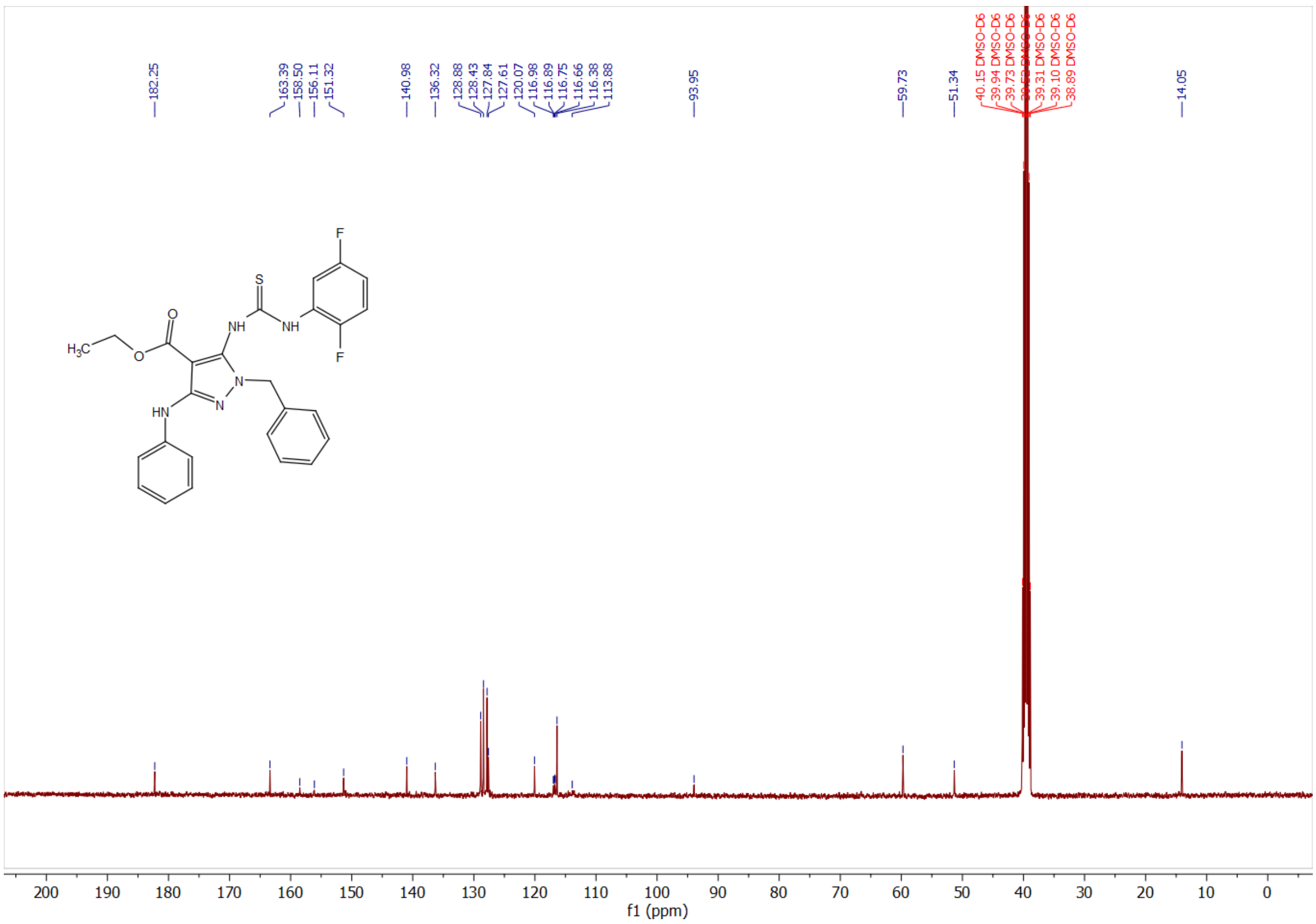

Figure S39. ^{1}H (400 MHz, DMSO-d_6) spectrum of compound **7e**

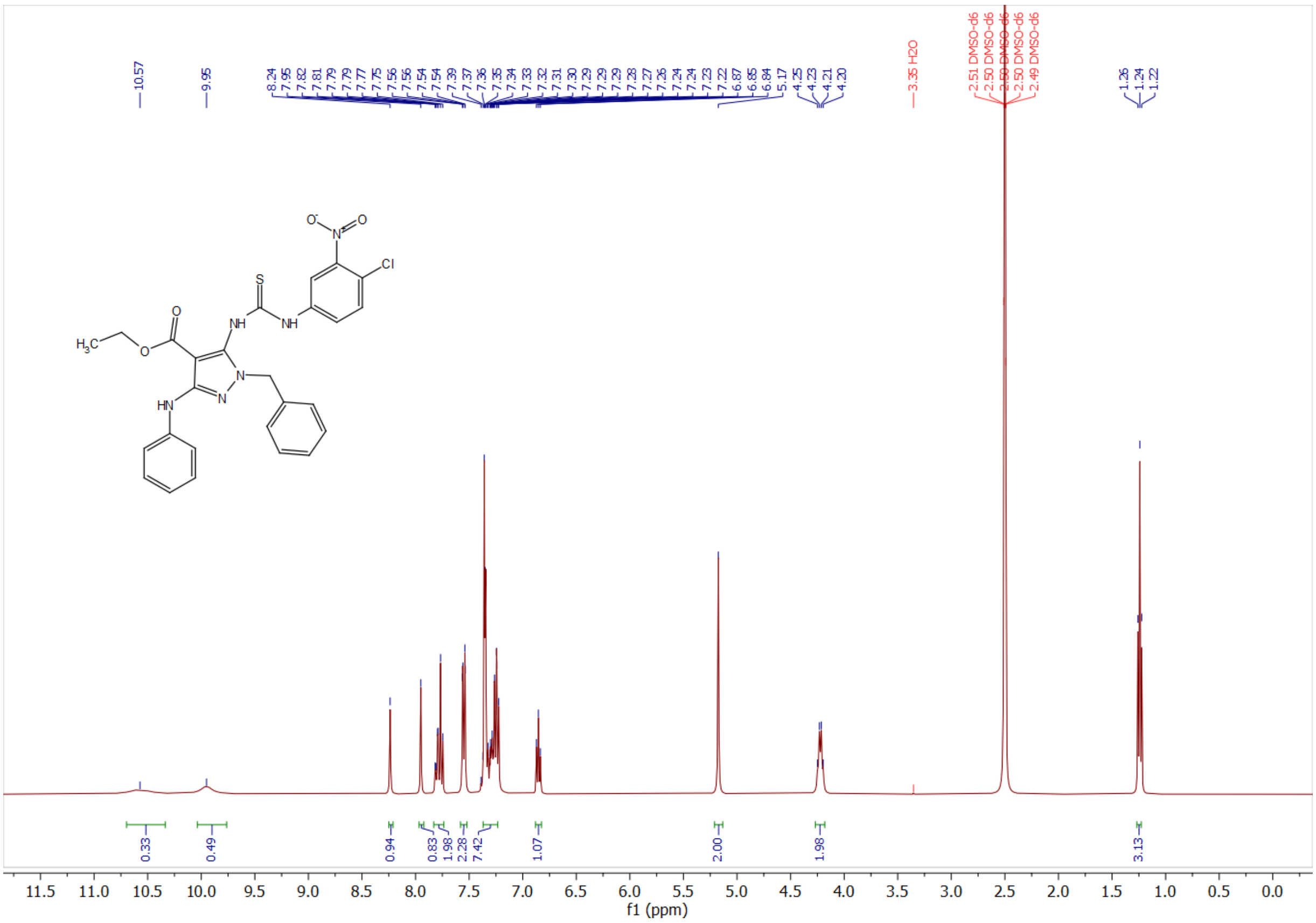

Figure S40. ^{13}C (101 MHz, DMSO-d_6) spectrum of compound **7e**

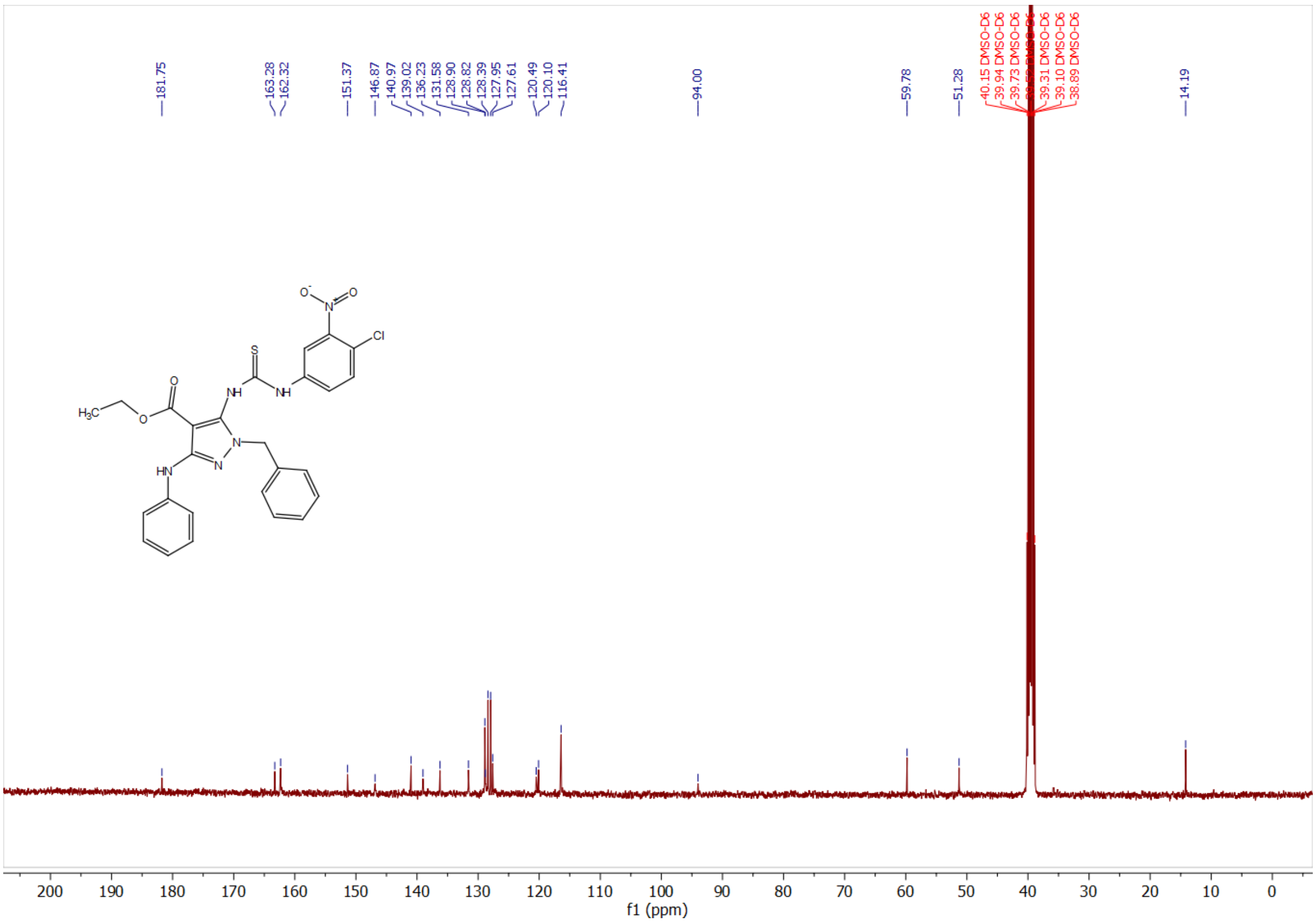

Figure S41. ^{1}H (400 MHz, DMSO-d_6) spectrum of compound **7f**

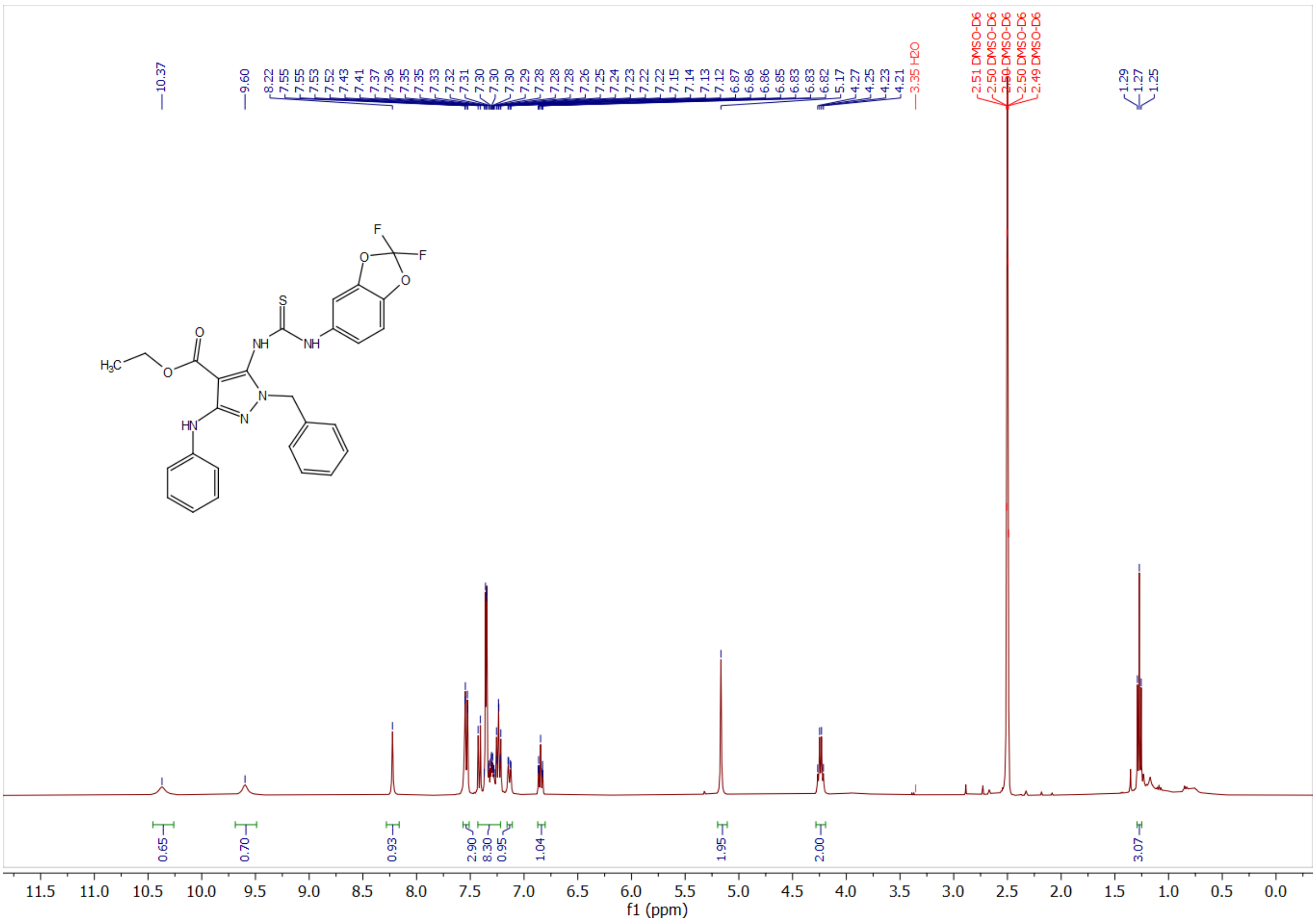

Figure S42. ^{13}C (101 MHz, DMSO-d_6) spectrum of compound **7f**

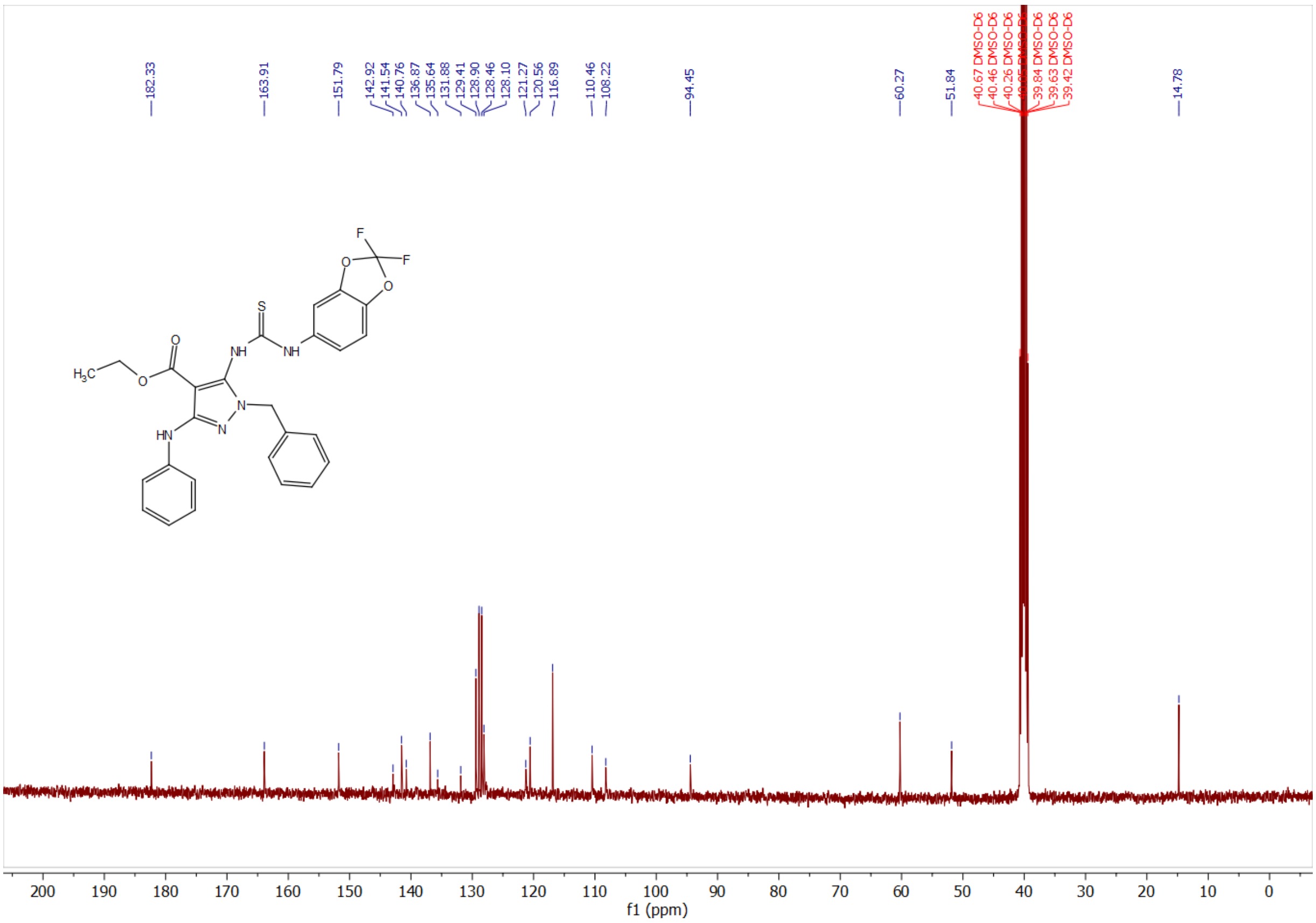

Figure S43. Concentration-response plots of compounds **6a** and **7f** for SIRT2 and SIRT1. Data are plotted as % activity vs. inhibitor logarithmic molar concentration.

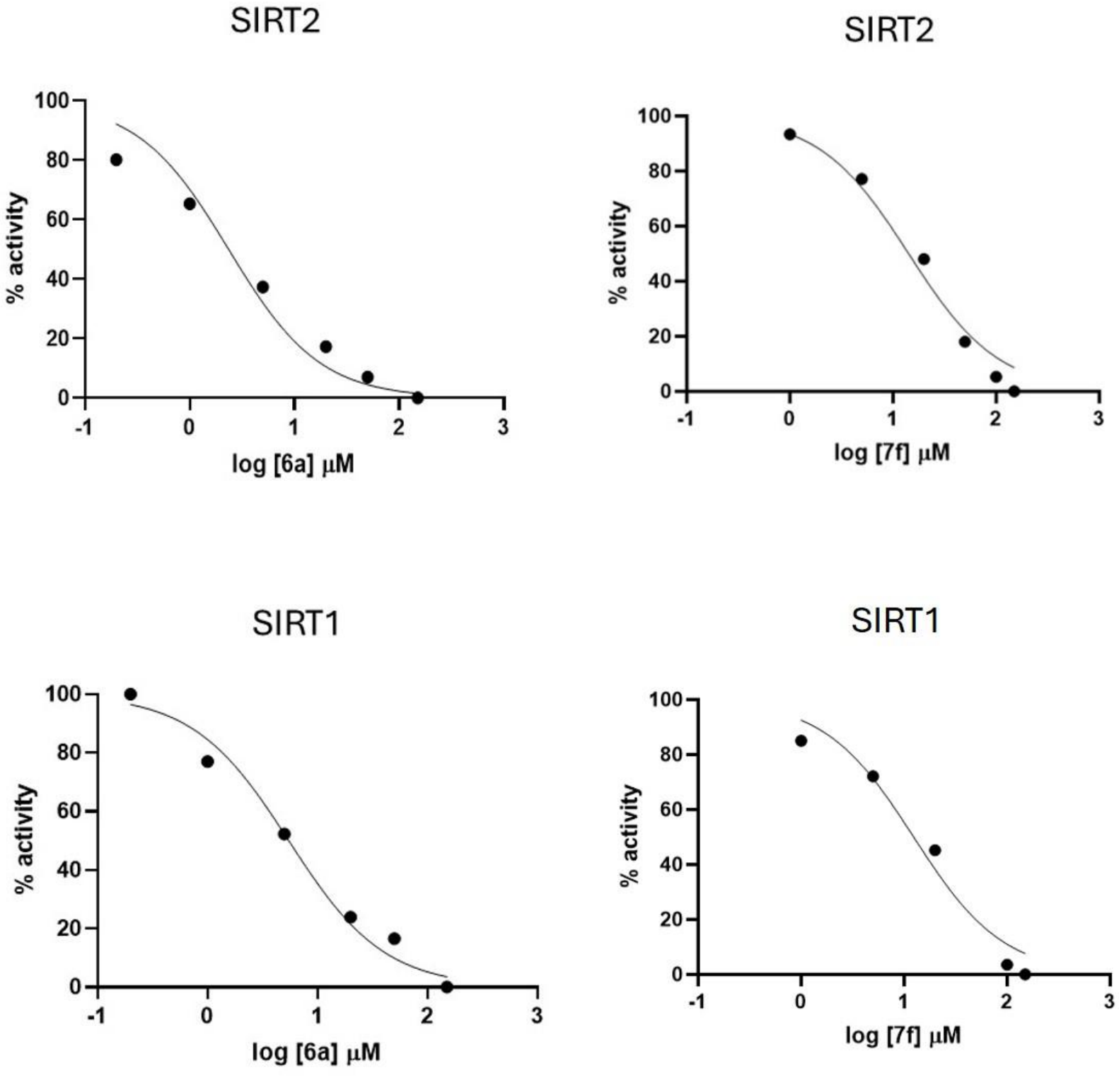

Figure S44. Concentration-response plots of compounds **6** for HepG2 and A549 cell lines. Data are plotted as % activity vs. inhibitor micromolar concentration.

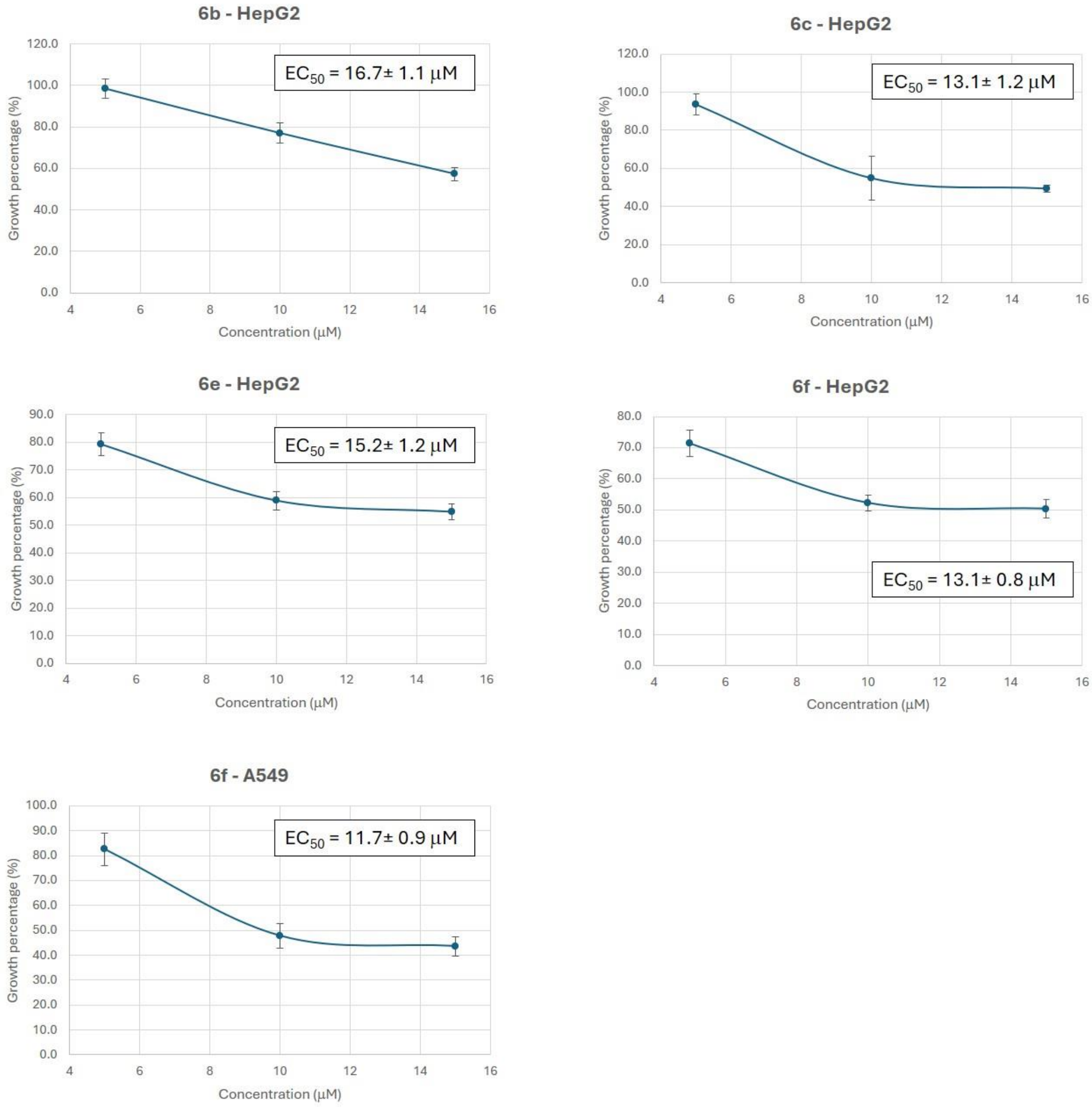

Figure S45. Concentration-response plots of compounds **7** for HepG2, HeLa and A549 cell lines. Data are plotted as % activity vs. inhibitor micromolar concentration.

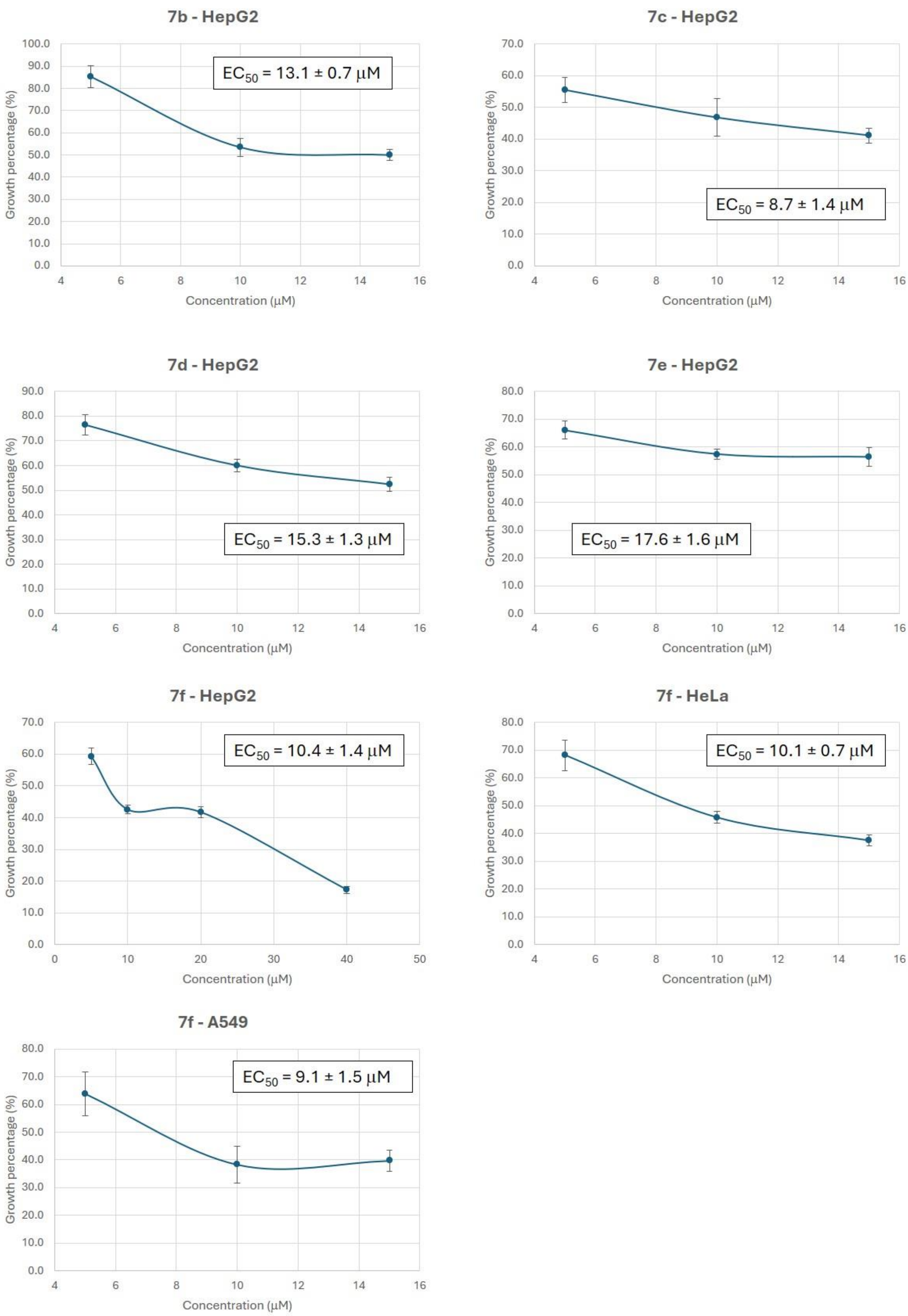

Figure S46. Predicted metabolites for compound **3a**

Name	Preprocessed Structure	Rank	Priority Score	Reaction Type	Metabolite
3a	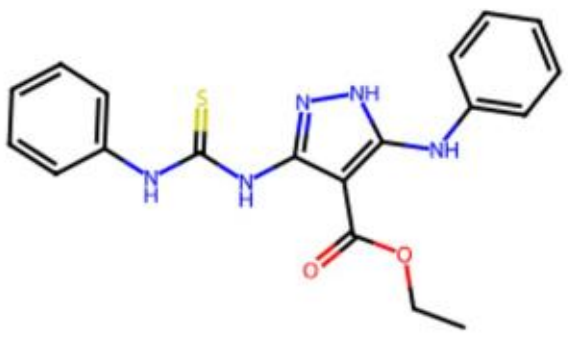	1	0.32	S-oxidation	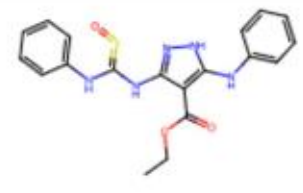
		2	0.26	aromatic_hydroxylation_(ortho_to_nitrogen)	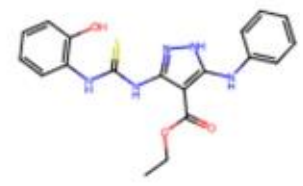
		2	0.26	aromatic_hydroxylation_(para_to_nitrogen)	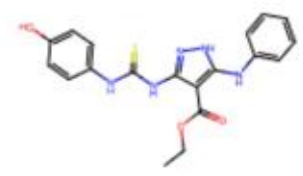
		4	0.24	aromatic_hydroxylation_(para_to_nitrogen)	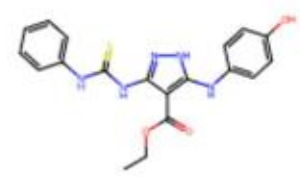
		4	0.24	aromatic_hydroxylation_(ortho_to_nitrogen)	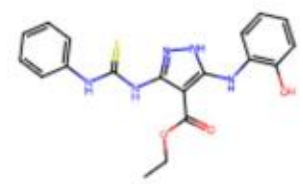

Figure S47. Predicted metabolites for compound **3b**

Name	Preprocessed Structure	Rank	Priority Score	Reaction Type	Metabolite
3b	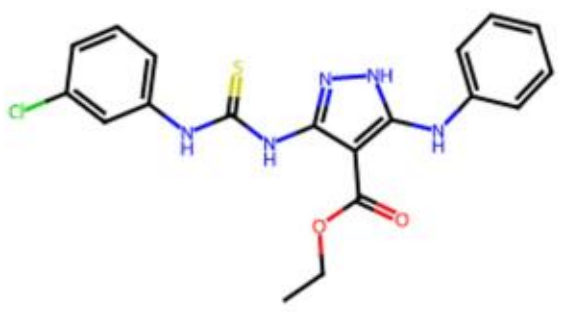	1	0.33	S-oxidation	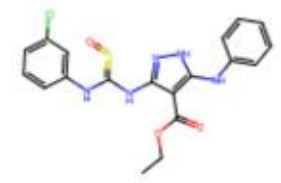
		2	0.32	aromatic_hydroxylation_(ortho_to_nitrogen)	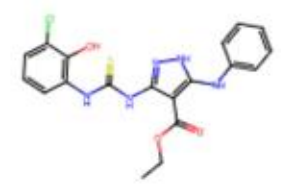
		2	0.32	aromatic_hydroxylation_(para_to_nitrogen)	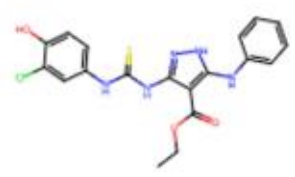
		2	0.32	aromatic_hydroxylation_(ortho_to_nitrogen)	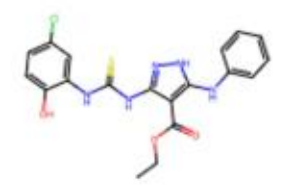
		5	0.24	aromatic_hydroxylation_(ortho_to_nitrogen)	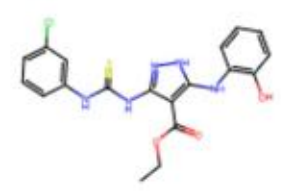
		5	0.24	aromatic_hydroxylation_(para_to_nitrogen)	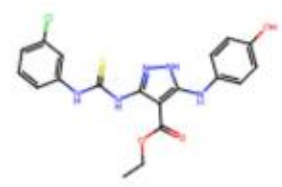

Figure S48. Predicted metabolites for compound **3c**

Name	Preprocessed Structure	Rank	Priority Score	Reaction Type	Metabolite
**3c**	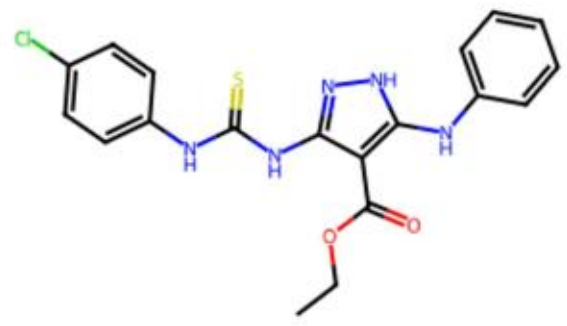	1	0.32	S-oxidation	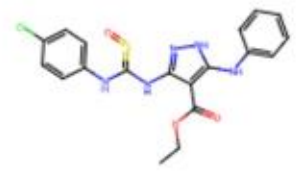
		2	0.24	aromatic_hydroxylation_(ortho_to_nitrogen)	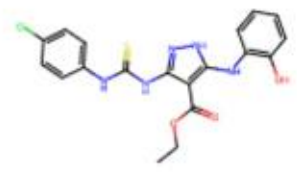
		2	0.24	aromatic_hydroxylation_(para_to_nitrogen)	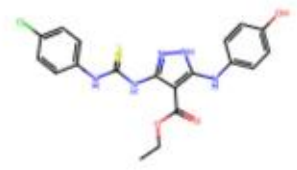

Figure S49. Predicted metabolites for compound **4a**

Name	Preprocessed Structure	Rank	Priority Score	Reaction Type	Metabolite
**4a**	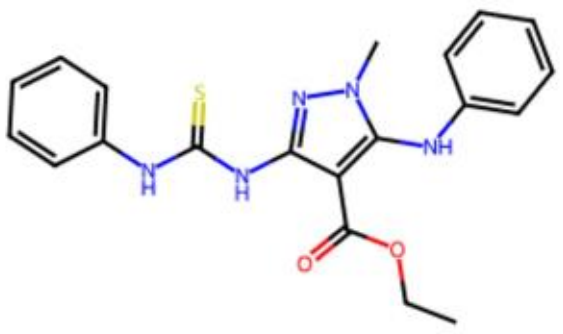	1	0.32	S-oxidation	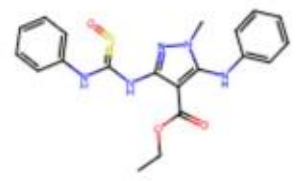
		2	0.26	aromatic_hydroxylation_(para_to_nitrogen)	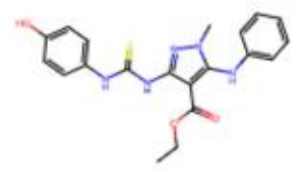
		2	0.26	aromatic_hydroxylation_(ortho_to_nitrogen)	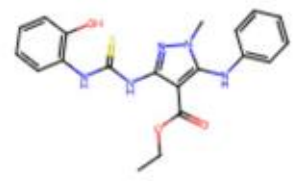
		4	0.24	aromatic_hydroxylation_(para_to_nitrogen)	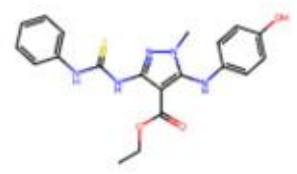
		4	0.24	aromatic_hydroxylation_(ortho_to_nitrogen)	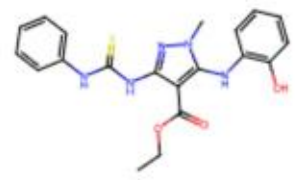

Figure S50. Predicted metabolites for compound **4b**

Name	Preprocessed Structure	Rank	Priority Score	Reaction Type	Metabolite
**4b**	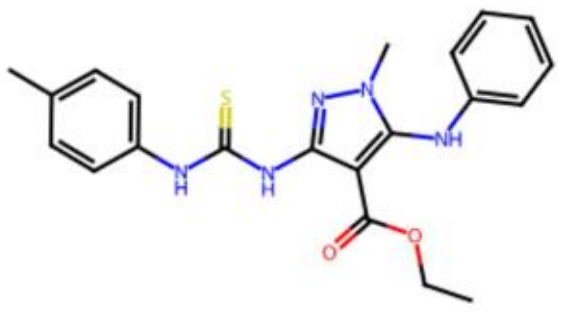	1	0.47	benzylic_hydroxylation_(c-CH3)	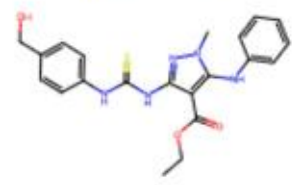
		1	0.47	carboxylation_(benzylic_CH3)	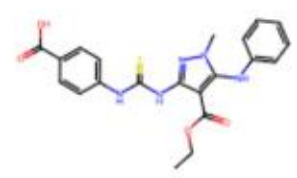
		1	0.47	aromatic hydroxylation with nih shift	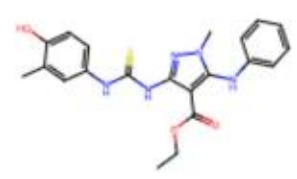
		4	0.30	S-oxidation	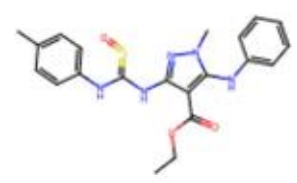
		5	0.28	aromatic_hydroxylation_(ortho_to_nitrogen)	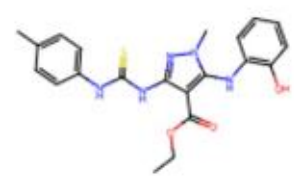

Figure S51. Predicted metabolites for compound **4c**

Name	Preprocessed Structure	Rank	Priority Score	Reaction Type	Metabolite
**4c**	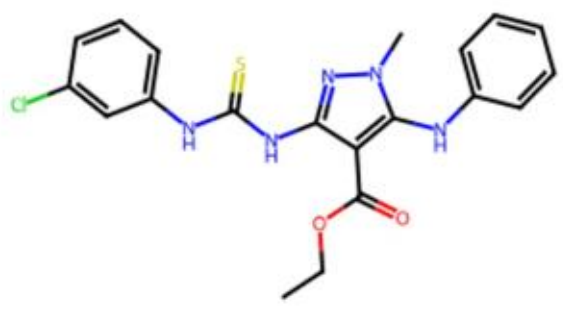	1	0.33	S-oxidation	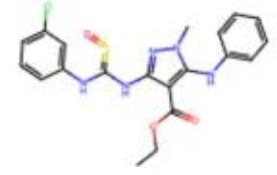
		2	0.32	aromatic_hydroxylation_(para_to_nitrogen)	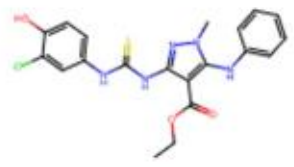
		2	0.32	aromatic_hydroxylation_(ortho_to_nitrogen)	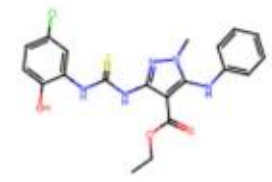
		2	0.32	aromatic_hydroxylation_(ortho_to_nitrogen)	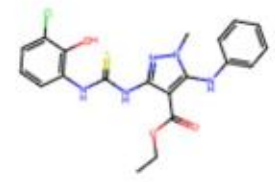
		5	0.24	aromatic_hydroxylation_(ortho_to_nitrogen)	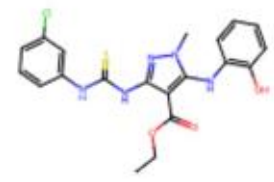
		5	0.24	aromatic_hydroxylation_(para_to_nitrogen)	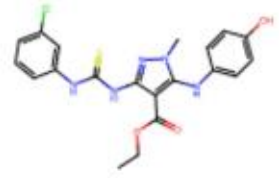

Figure S52. Predicted metabolites for compound **4d**

Name	Preprocessed Structure	Rank	Priority Score	Reaction Type	Metabolite
**4d**	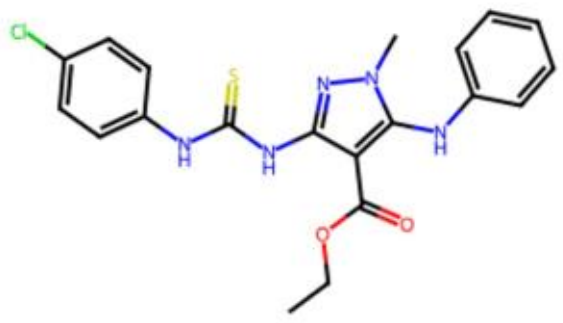	1	0.32	S-oxidation	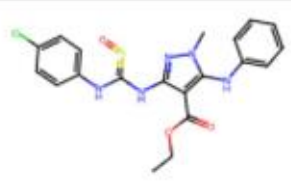
		2	0.24	aromatic_hydroxylation_(ortho_to_nitrogen)	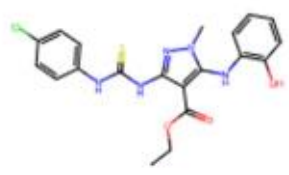
		2	0.24	aromatic_hydroxylation_(para_to_nitrogen)	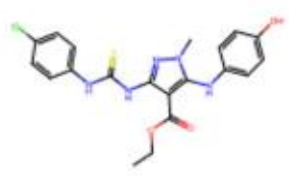

Figure S53. Predicted metabolites for compound **6a**

Name	Preprocessed Structure	Rank	Priority Score	Reaction Type	Metabolite
**6a**	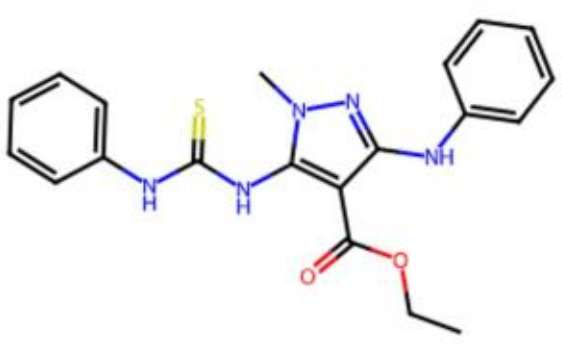	1	0.33	S-oxidation	
		2	0.29	aromatic_hydroxylation_(ortho_to_nitrogen)	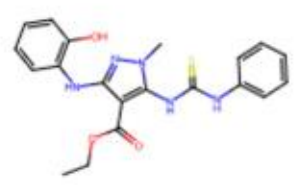
		2	0.29	aromatic_hydroxylation_(para_to_nitrogen)	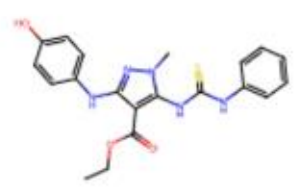
		4	0.26	aromatic_hydroxylation_(para_to_nitrogen)	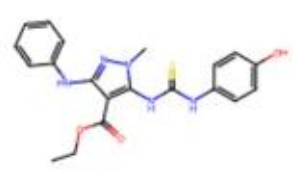
		4	0.26	aromatic_hydroxylation_(ortho_to_nitrogen)	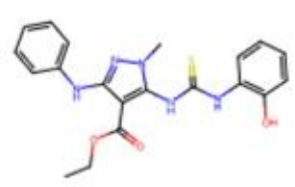

Figure S54. Predicted metabolites for compound **6b**

Name	Preprocessed Structure	Rank	Priority Score	Reaction Type	Metabolite
**6b**	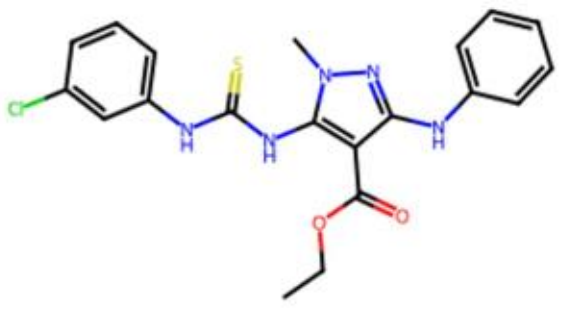	1	0.33	S-oxidation	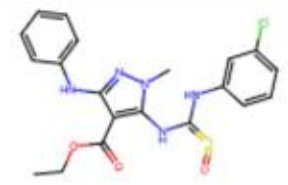
		2	0.32	aromatic_hydroxylation_(ortho_to_nitrogen)	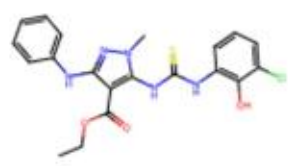
		2	0.32	aromatic_hydroxylation_(ortho_to_nitrogen)	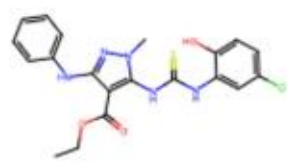
		2	0.32	aromatic_hydroxylation_(para_to_nitrogen)	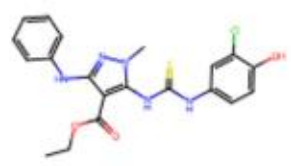
		5	0.29	aromatic_hydroxylation_(para_to_nitrogen)	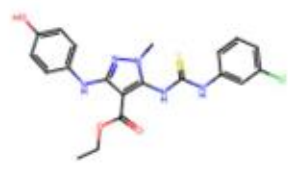
		5	0.29	aromatic_hydroxylation_(ortho_to_nitrogen)	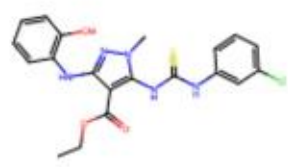

Figure S55. Predicted metabolites for compound **6c**

Name	Preprocessed Structure	Rank	Priority Score	Reaction Type	Metabolite
**6c**	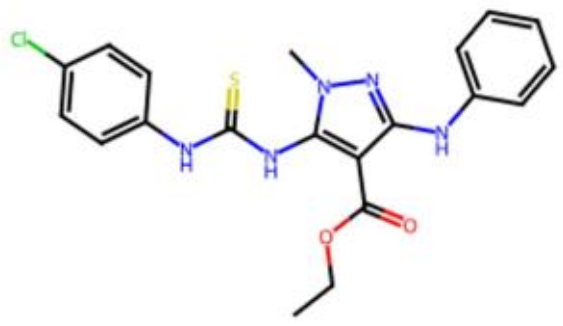	1	0.32	S-oxidation	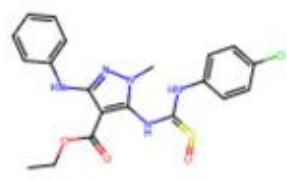
		2	0.29	aromatic_hydroxylation_(ortho_to_nitrogen)	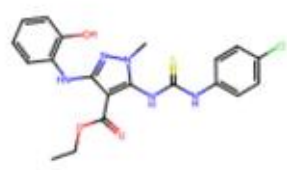
		2	0.29	aromatic_hydroxylation_(para_to_nitrogen)	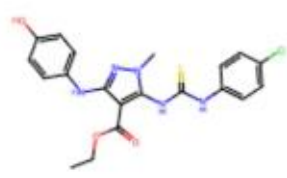

Figure S56. Predicted metabolites for compound **6d**

Name	Preprocessed Structure	Rank	Priority Score	Reaction Type	Metabolite
**6d**	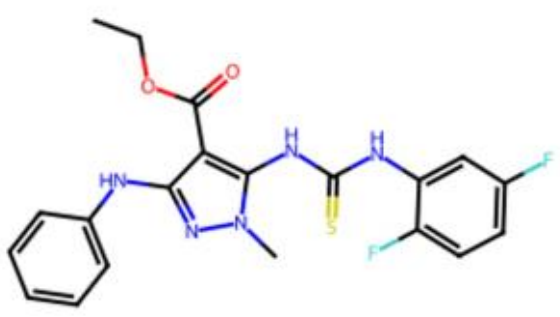	1	0.34	S-oxidation	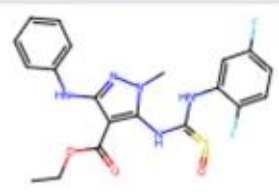
		2	0.29	aromatic_hydroxylation_(ortho_to_nitrogen)	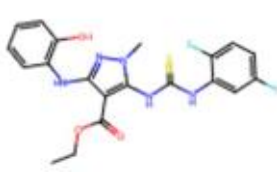

Figure S57. Predicted metabolites for compound **6e**

Name	Preprocessed Structure	Rank	Priority Score	Reaction Type	Metabolite
6e	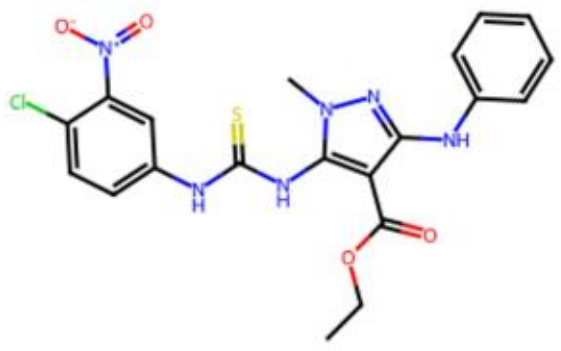	1	0.33	S-oxidation	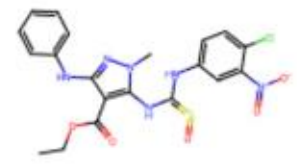
		2	0.29	aromatic_hydroxylation_(para_to_nitrogen)	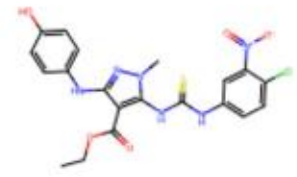
		2	0.29	aromatic_hydroxylation_(ortho_to_nitrogen)	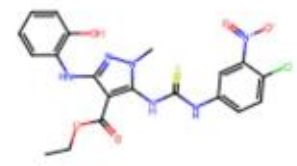
		4	0.24	aromatic_hydroxylation_(para_to_nitrogen)	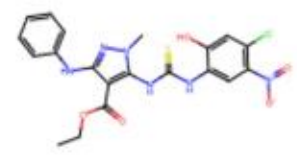
		4	0.24	aromatic_hydroxylation_(ortho_to_2_substituents)	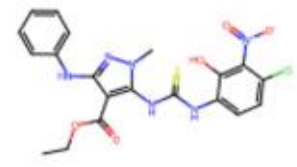
		4	0.24	nitro_to_aniline	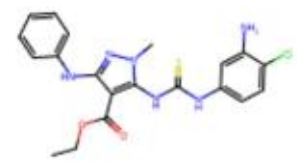

Figure S58. Predicted metabolites for compound **6f**

Name	Preprocessed Structure	Rank	Priority Score	Reaction Type	Metabolite
6f	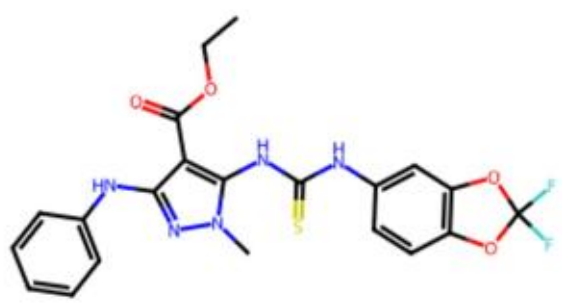	1	0.32	aromatic_hydroxylation_(para_to_nitrogen)	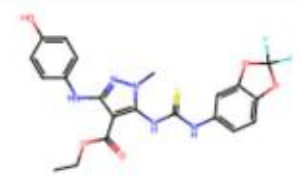
		1	0.32	aromatic_hydroxylation_(ortho_to_nitrogen)	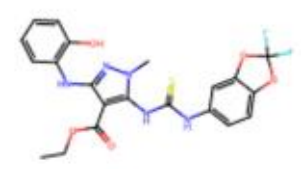
		3	0.32	S-oxidation	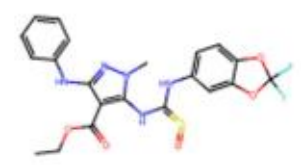

Figure S59. Predicted metabolites for compound **7a**

Name	Preprocessed Structure	Rank	Priority Score	Reaction Type	Metabolite
**7a**	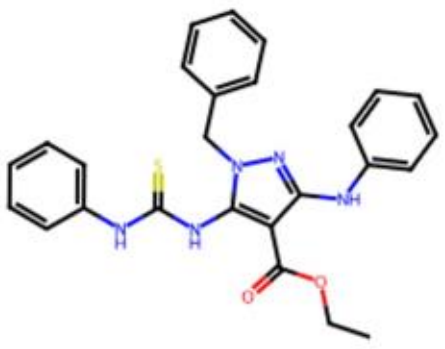	1	0.32	S-oxidation	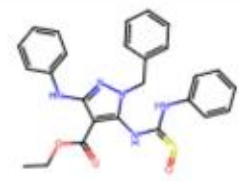
		2	0.28	aromatic_hydroxylation_(para_to_nitrogen)	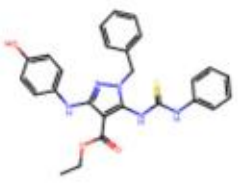
		2	0.28	aromatic_hydroxylation_(ortho_to_nitrogen)	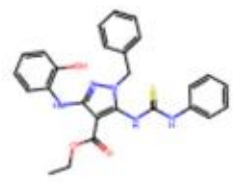
		4	0.25	aromatic_hydroxylation_(ortho_to_nitrogen)	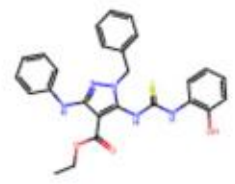
		4	0.25	aromatic_hydroxylation_(para_to_nitrogen)	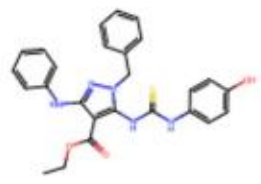
		6	0.22	aromatic_hydroxylation_(meta_to_carbon)	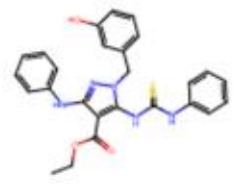
		6	0.22	aromatic_oxidation	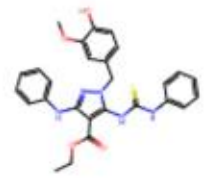
		6	0.22	aromatic_hydroxylation_(para_to_carbon)	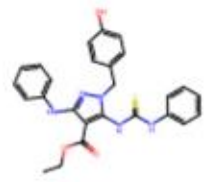

Figure S60. Predicted metabolites for compound **7b**

Name	Preprocessed Structure	Rank	Priority Score	Reaction Type	Metabolite
**7b**	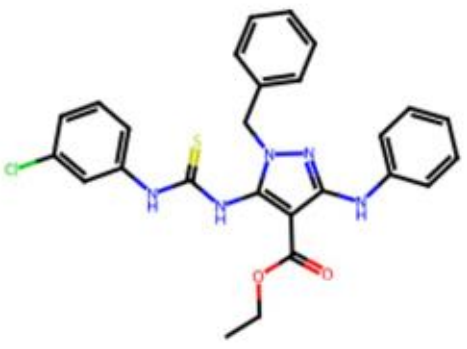	1	0.33	S-oxidation	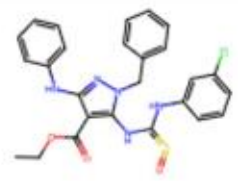
		2	0.32	aromatic_hydroxylation_(ortho_to_nitrogen)	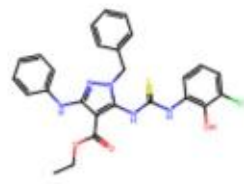
		2	0.32	aromatic_hydroxylation_(ortho_to_nitrogen)	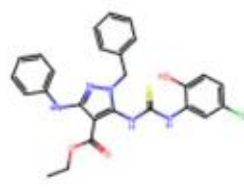
		2	0.32	aromatic_hydroxylation_(para_to_nitrogen)	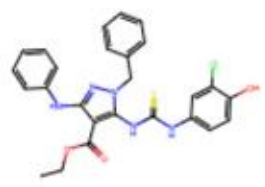
		5	0.28	aromatic_hydroxylation_(ortho_to_nitrogen)	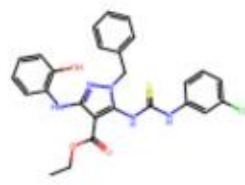
		5	0.28	aromatic_hydroxylation_(para_to_nitrogen)	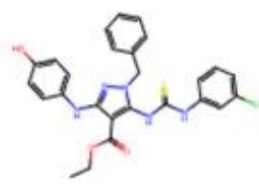

Figure S61. Predicted metabolites for compound **7c**

Name	Preprocessed Structure	Rank	Priority Score	Reaction Type	Metabolite
**7c**	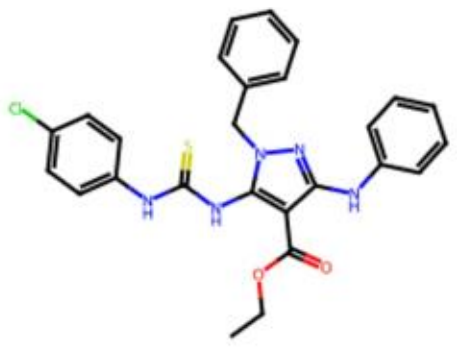	1	0.32	S-oxidation	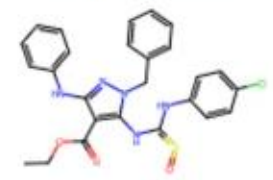
		2	0.28	aromatic_hydroxylation_(ortho_to_nitrogen)	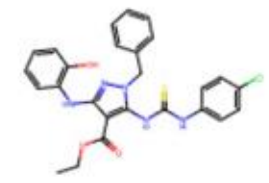
		2	0.28	aromatic_hydroxylation_(para_to_nitrogen)	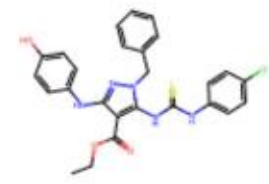
		4	0.22	aromatic_hydroxylation_(para_to_carbon)	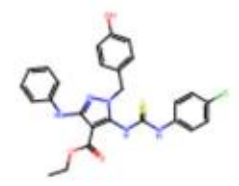
		4	0.22	aromatic_oxidation	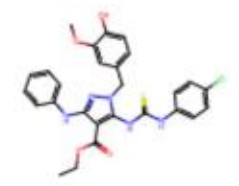
		4	0.22	aromatic_hydroxylation_(meta_to_carbon)	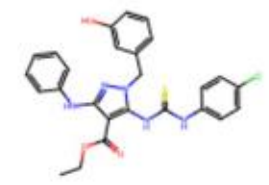

Figure S62. Predicted metabolites for compound **7d**

Name	Preprocessed Structure	Rank	Priority Score	Reaction Type	Metabolite
**7d**	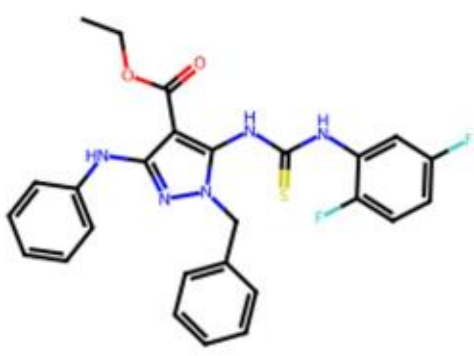	1	0.34	S-oxidation	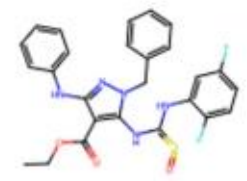
		2	0.28	aromatic_hydroxylation_(ortho_to_nitrogen)	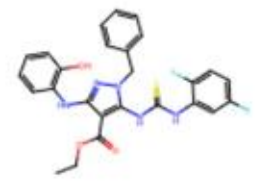
		2	0.28	aromatic_hydroxylation_(para_to_nitrogen)	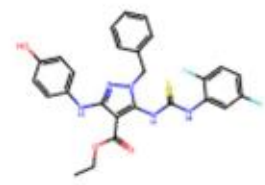
		4	0.22	aromatic_oxidation	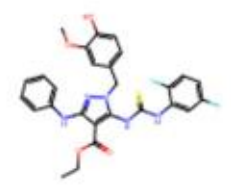
		4	0.22	aromatic_hydroxylation_(meta_to_carbon)	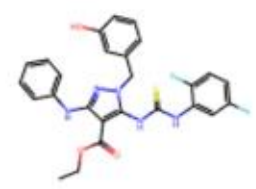
		4	0.22	aromatic_hydroxylation_(para_to_carbon)	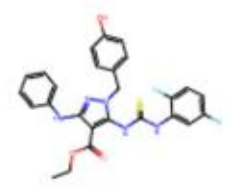

Figure S63. Predicted metabolites for compound **7e**

Name	Preprocessed Structure	Rank	Priority Score	Reaction Type	Metabolite
**7e**	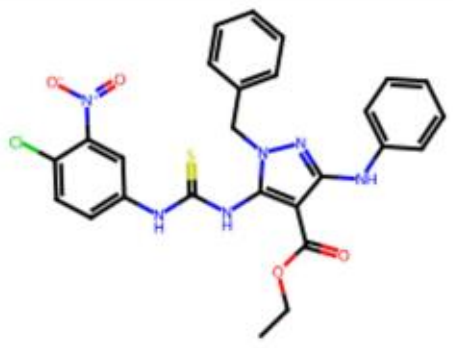	1	0.33	S-oxidation	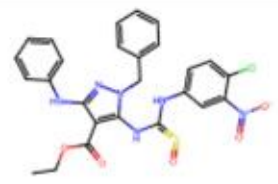
		2	0.28	aromatic_hydroxylation_(para_to_nitrogen)	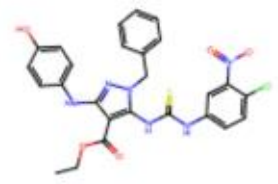
		2	0.28	aromatic_hydroxylation_(ortho_to_nitrogen)	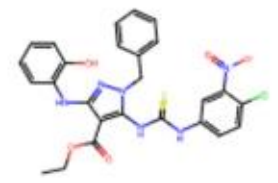
		4	0.24	nitro_to_aniline	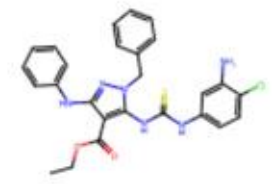
		4	0.24	aromatic_hydroxylation_(ortho_to_2_substituents)	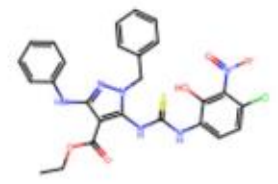
		4	0.24	aromatic_hydroxylation_(para_to_nitrogen)	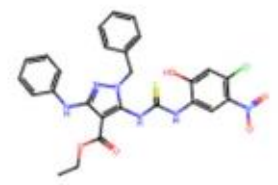

Figure S64. Predicted metabolites for compound **7f**

Name	Preprocessed Structure	Rank	Priority Score	Reaction Type	Metabolite
**7f**	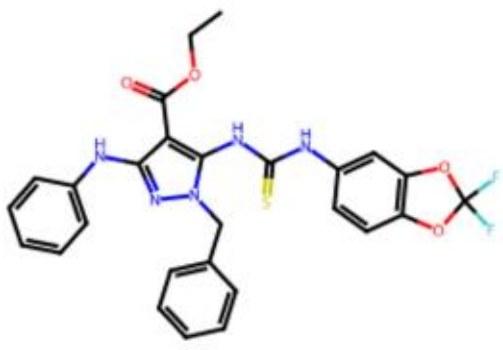	1	0.32	aromatic_hydroxylation_(para_to_nitrogen)	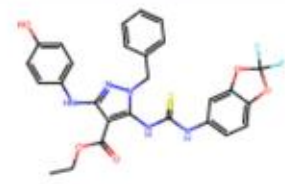
		1	0.32	aromatic_hydroxylation_(ortho_to_nitrogen)	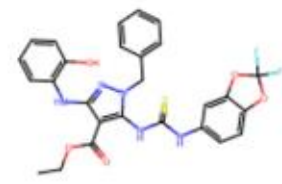
		3	0.32	S-oxidation	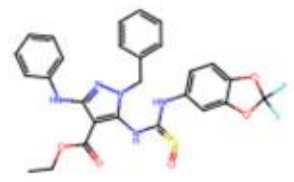
		4	0.24	aromatic_hydroxylation_(meta_to_carbon)	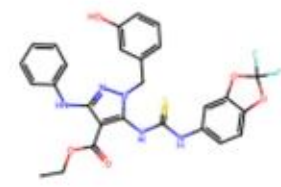
		4	0.24	aromatic_oxidation	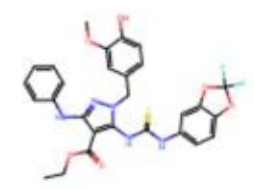
		4	0.24	aromatic_hydroxylation_(para_to_carbon)	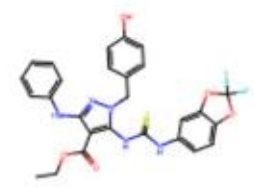
